# Metabolomic age (MileAge) predicts health and life span: A comparison of multiple machine learning algorithms

**DOI:** 10.1126/sciadv.adp3743

**Published:** 2024-12-18

**Authors:** Julian Mutz, Raquel Iniesta, Cathryn M. Lewis

**Affiliations:** ^1^ Department of Biostatistics and Health Informatics, King’s College London, London, United Kingdom.; ^2^ Social, Genetic and Developmental Psychiatry Centre, Institute of Psychiatry, Psychology & Neuroscience, King’s College London, London, United Kingdom.; ^3^ Department of Medical and Molecular Genetics, Faculty of Life Sciences and Medicine, King’s College London, London, United Kingdom.

## Abstract

Biological aging clocks produce age estimates that can track with age-related health outcomes. This study aimed to benchmark machine learning algorithms, including regularized regression, kernel-based methods, and ensembles, for developing metabolomic aging clocks from nuclear magnetic resonance spectroscopy data. The UK Biobank data, including 168 plasma metabolites from up to *N* = 225,212 middle-aged and older adults (mean age, 56.97 years), were used to train and internally validate 17 algorithms. Metabolomic age (MileAge) delta, the difference between metabolite-predicted and chronological age, from a Cubist rule–based regression model showed the strongest associations with health and aging markers. Individuals with an older MileAge were frailer, had shorter telomeres, were more likely to suffer from chronic illness, rated their health worse, and had a higher all-cause mortality hazard (HR = 1.51; 95% CI, 1.43 to 1.59; *P* < 0.001). This metabolomic aging clock (MileAge) can be applied in research and may find use in health assessments, risk stratification, and proactive health tracking.

## Teaser

Machine learning–based metabolomic aging clocks predict health and death, with nonlinear models better capturing aging signals.

## INTRODUCTION

Chronological age, the time since birth, is a powerful predictor of health and disease (*1*). However, individuals of the same age show considerable heterogeneity in health status, lifestyle, and the physical signs of aging. This variation partly reflects differences in biological aging, the process of accumulating molecular and cellular damage that impairs physiological functioning (*2*). While chronological age cannot be altered, biological aging trajectories may be modifiable. Therefore, developing reliable measures of biological age is a priority in biomedical research and population health.

Although no single marker defines biological aging, several hallmarks, such as telomere attrition, have been identified (*3*). Clinical and population studies of age-related biological changes in humans have examined numerous potential measures, including grip strength and cardiovascular function (*4*–*6*), blood-based biomarkers (*7*), inflammatory markers (*8*), telomere length (*9*), and frailty (*10*).

The increasing availability of high-dimensional molecular omics and neuroimaging data, for example, DNA methylation (DNAm) (*11*–*13*) and magnetic resonance imaging (*14*), has enabled the development of biological aging clocks (*15*, *16*). These clocks are typically developed using statistical or machine learning algorithms that identify relationships between chronological age and molecular data. The difference between predicted age and chronological age can track with health outcomes (*17*). Aging clocks provide a more holistic view of a person’s health and are more readily interpretable than many individual molecular markers, as they are expressed in units of years.

Metabolomics, the study of small molecules within cells, tissues, or organisms, is increasingly incorporated into biological aging research (*18*). Metabolites are the end products of metabolism, such as when food is converted to energy. Early metabolomics studies were limited to a few metabolites and small samples, but technological advancements have enabled the population-scale profiling of multiple molecular pathways (*19*). Quantifying hundreds or thousands of metabolites can provide detailed snapshots of an individual’s physiological state. Metabolomic profiles can predict many common incident diseases (*20*) and mortality risk (*21*, *22*).

Over the past decade, several studies have explored the links between chronological age and metabolites (*23*–*25*). The first study to develop a metabolite-derived age variable showed that a panel of 22 metabolites explained 59% of the variance in chronological age. A linear combination of these metabolites was correlated with age-related clinical measures, independent of chronological age (*24*). The first study to develop an aging clock from metabolomics data showed that the difference between metabolite-predicted and chronological age was associated with a higher disease burden and mortality (*26*). Subsequent studies, for example, those in the Airwave Health Monitoring Study in the UK (*27*) and the Dutch BBMRI-NL consortium (*28*), have replicated some of these findings. Metabolomics is recognized as one of the most powerful omics data for age estimation (*16*) and disease prediction (*17*).

The aim of this study was to compare multiple machine learning algorithms for developing metabolomic aging clocks using nuclear magnetic resonance (NMR) spectroscopy data in the UK Biobank (*29*). To assess the ability of these aging clocks to predict age-related morbidity and life span and to capture biological signals beyond those approximated by chronological age (*30*), we examined their associations with multiple health and aging markers (e.g., telomere length and frailty) and all-cause mortality. This study presents a comprehensive comparison of machine learning algorithms for developing metabolomic aging clocks, benchmarking a wide range of models under consistent conditions in one of the largest metabolomics datasets available globally. Selecting a broad array of algorithms enabled us to identify those that best used the data, enhancing the predictive performance, robustness, and interpretability of metabolomic aging clocks. We hypothesized that nonlinear models may better capture the complex relationships between metabolites and age, an area that remains underexplored (*18*). However, aging clocks derived from models with lower accuracy at predicting age, such as linear algorithms, may not necessarily perform worse at predicting age-related health outcomes (*31*). As such, we remained agnostic as to whether aging clocks derived from more complex algorithms would result in stronger associations with health and mortality.

## RESULTS

### Sample characteristics

Of the 118,019 participants in the first release of metabolomics data, 110,730 had complete information on all metabolites (fig. S1). After removing individuals with outlier metabolite values, inconsistencies between self-reported sex and genetic sex or possible pregnancies, the analytical sample included 101,359 participants (Table 1 and Fig. 1A). The mean chronological age was 56.44 years (SD, 8.12), with the most common age being 61 years (Fig. 1B). Most metabolite levels varied by chronological age (Fig. 1C), showing considerable evidence of nonlinear relationships (figs. S2 to S33).

**Table 1.** Sample characteristics. Note: GCSE, general certificate of secondary education; CSE, certificate of secondary education; NVQ, national vocational qualification; HND, higher national diploma; HNC, higher national certificate. MileAge delta (adj.) derived from the Cubist rule–based regression model (trained on *N* = 101,359).

		MileAge delta (adj.)		
	Analytical sample(*N* = 101,359)	≤ 1SD below the mean(*N* = 16,204)	Middle(*N* = 69,166)	≥ 1SD above the mean(*N* = 15,989)
Age; mean (SD)	56.44 (8.12)	55.85 (8.33)	56.75 (8.08)	55.69 (8.00)
Sex				
Female	54,484 (53.8%)	8,062 (49.8%)	37,002 (53.5%)	9,420 (58.9%)
Male	46,875 (46.2%)	8,142 (50.2%)	32,164 (46.5%)	6,569 (41.1%)
Ethnicity				
White	95,672 (94.4%)	15,163 (93.6%)	65,486 (94.7%)	15,023 (94.0%)
Mixed-race	574 (0.6%)	103 (0.6%)	386 (0.6%)	85 (0.5%)
Black	1,529 (1.5%)	233 (1.4%)	975 (1.4%)	321 (2.0%)
Asian	1,953 (1.9%)	375 (2.3%)	1,265 (1.8%)	313 (2.0%)
Chinese	291 (0.3%)	77 (0.5%)	184 (0.3%)	30 (0.2%)
Other	880 (0.9%)	173 (1.1%)	564 (0.8%)	143 (0.9%)
Missing*	460 (0.5%)	80 (0.5%)	306 (0.4%)	74 (0.5%)
Highest qualification				
None	17,084 (16.9%)	2,551 (15.7%)	11,868 (17.2%)	2,665 (16.7%)
O levels/GCSEs/CSEs	27,008 (26.6%)	4,237 (26.1%)	18,435 (26.7%)	4,336 (27.1%)
A levels/NVQ/HND/HNC†	23,235 (22.9%)	3,637 (22.4%)	15,961 (23.1%)	3,637 (22.7%)
Degree	32,826 (32.4%)	5,577 (34.4%)	22,091 (31.9%)	5,158 (32.3%)
Missing*	1,206 (1.2%)	202 (1.2%)	811 (1.2%)	193 (1.2%)
Household income^‡^				
Very low	19,422 (19.2%)	2,863 (17.7%)	13,265 (19.2%)	3,294 (20.6%)
Low	22,159 (21.9%)	3,487 (21.5%)	15,137 (21.9%)	3,535 (22.1%)
Medium	22,634 (22.3%)	3,831 (23.6%)	15,417 (22.3%)	3,386 (21.2%)
High	17,767 (17.5%)	3,095 (19.1%)	12,027 (17.4%)	2,645 (16.5%)
Very high	4,725 (4.7%)	819 (5.1%)	3,188 (4.6%)	718 (4.5%)
Missing^*^	14,652 (14.5%)	2,109 (13.0%)	10,132 (14.6%)	2,411 (15.1%)
Cohabitation				
With partner	73,896 (72.9%)	12,054 (74.4%)	50,748 (73.4%)	11,094 (69.4%)
Single	8,341 (8.2%)	1,418 (8.8%)	5,467 (7.9%)	1,456 (9.1%)
Missing*	19,122 (18.9%)	2,732 (16.9%)	12,951 (18.7%)	3,439 (21.5%)
Smoking status				
Never	55,643 (54.9%)	9,149 (56.5%)	37,876 (54.8%)	8,618 (53.9%)
Former	34,805 (34.3%)	5,342 (33.0%)	23,838 (34.5%)	5,625 (35.2%)
Current	10,416 (10.3%)	1,634 (10.1%)	7,119 (10.3%)	1,663 (10.4%)
Missing*	495 (0.5%)	79 (0.5%)	333 (0.5%)	83 (0.5%)
Menopause^§^				
No	12,986 (12.8%)	2,887 (17.8%)	8,096 (11.7%)	2,003 (12.5%)
Yes	32,745 (32.3%)	4,046 (25.0%)	23,034 (33.3%)	5,665 (35.4%)
Missing*	55,628 (54.9%)	9,271 (57.2%)	38,036 (55.0%)	8,321 (52.0%)
Morbidity count				
None	23,788 (23.5%)	4,459 (27.5%)	16,311 (23.6%)	3,018 (18.9%)
One	26,923 (26.6%)	4,581 (28.3%)	18,615 (26.9%)	3,727 (23.3%)
Two	20,535 (20.3%)	3,195 (19.7%)	14,011 (20.3%)	3,329 (20.8%)
Three	13,157 (13.0%)	1,857 (11.5%)	9,001 (13.0%)	2,299 (14.4%)
Four	7,553 (7.5%)	989 (6.1%)	5,077 (7.3%)	1,487 (9.3%)
Five+	9,387 (9.3%)	1,119 (6.9%)	6,141 (8.9%)	2,127 (13.3%)
Missing*	16 (0.0%)	4 (0.0%)	10 (0.0%)	2 (0.0%)
Fasting time¶; mean (SD)	3.75 (2.40)	3.74 (2.47)	3.74 (2.39)	3.79 (2.38)
Body mass index#; mean (SD)	27.43 (4.73)	26.17 (4.07)	27.41 (4.57)	28.80 (5.59)

*Missing data may also include “do not know,” “prefer not to answer,” “not applicable,” or related responses.

†Also includes “other professional qualifications.”

‡Annual household income groups: very low (<£18,000), low (£18,000 to £30,999), middle (£31,000 to £51,999), high (£52,000 to £100,000), and very high (>£100,000).

§*N* = 59,519 females.

¶*N* = 3 missing.

#*N* = 345 missing.

**Fig. 1.**
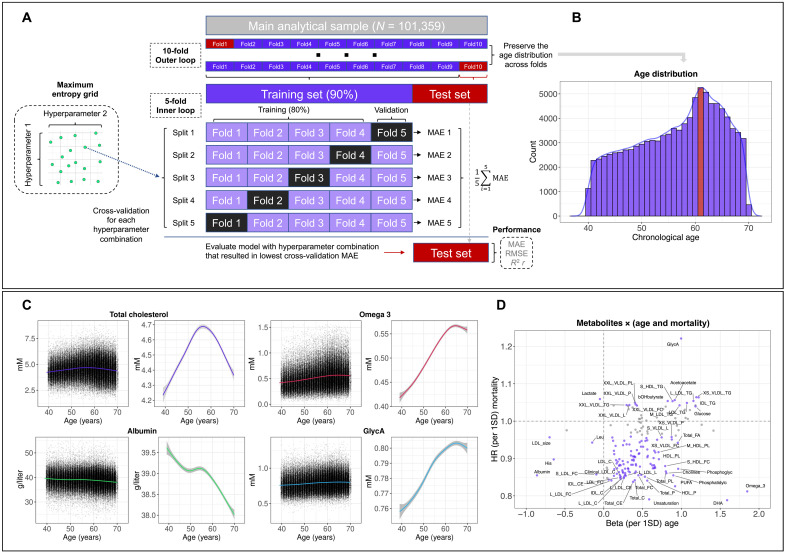
Study design and overview. (**A**) Overview of the nested cross-validation approach. MAE, mean absolute error; RMSE, root mean square error. (**B**) Histogram of the chronological age distribution of the analytical sample. The statistical mode (age, 61 years) is shown in red. (**C**) Distribution of metabolite levels by chronological age, showing scatter plots of all observations and smooth curves (note the difference in the *y*-axis scale). The smooth curves were estimated using generalized additive models, with shaded areas corresponding to 95% confidence intervals (CIs). GlycA, glycoprotein acetyls. (**D**) Scatter plot showing the hazard ratio (HR) for all-cause mortality and the beta for chronological age associated with a one SD difference in metabolite levels. Metabolites that had statistically significant associations with both chronological age and all-cause mortality are shown in purple. See the Supplementary Materials for full metabolite names. [(A) and (D)] *N* = 101,359, except for mortality (*N* = 101,274).

### Metabolite-wide associations

One hundred sixty-five of the 168 metabolites were associated with chronological age (*P* < 0.05/168). While most metabolite levels were elevated in older individuals (e.g., omega-3, citrate, and glucose), seven, including albumin, glycine, and histidine, were negatively associated with chronological age (fig. S34 and data S1). Among the 119 metabolites associated with all-cause mortality, glycoprotein acetyls (GlycA) were most strongly associated with a higher mortality hazard [HR = 1.22; 95% confidence interval (CI), 1.20 to 1.25; *P* < 0.001], whereas the degree of unsaturation, docosahexaenoic acid (DHA), and omega-3 were strongly associated with a lower mortality hazard (fig. S35 and data S2). Notably, 116 metabolites associated with chronological age also predicted mortality, with GlycA, omega-3, and DHA among the most strongly associated metabolites of both age and mortality (Fig. 1D). Between 87 and 95% of the metabolites that were statistically significantly associated with other health and aging markers (e.g., frailty and health status) were associated with chronological age (fig. S36). The exception was telomere length for which only 53% of statistically significant metabolites were shared with chronological age.

### Predictive model performance

**Fig. 2.**
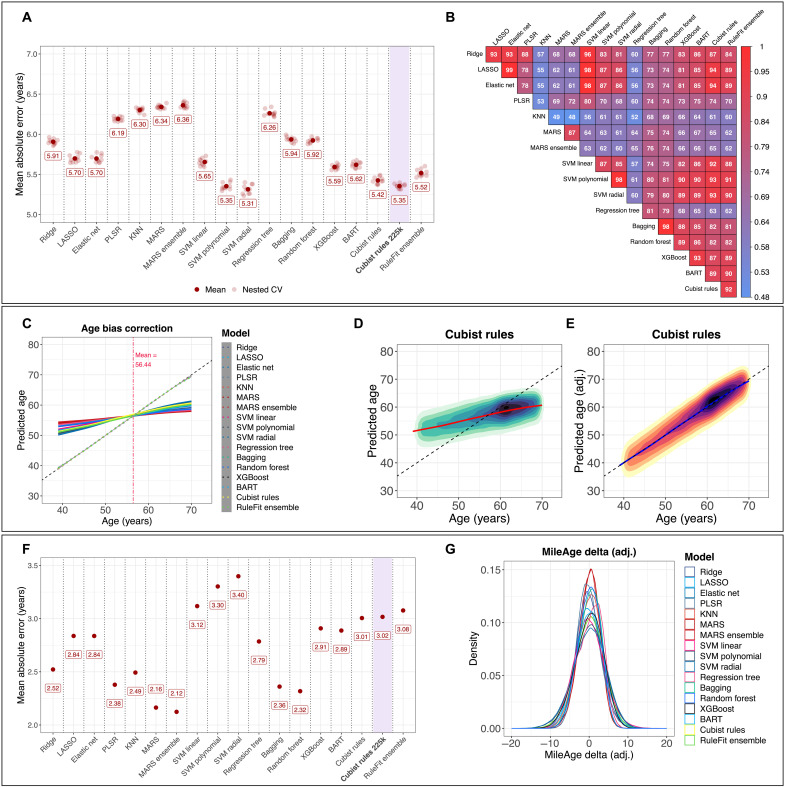
Predictive model performance. (**A**) Nested cross-validation MAE for all models with tuned hyperparameter values in the 10% test sets. CV, cross-validation. (**B**) Heatmap of Pearson’s correlation coefficient (*r*) between the predicted age values for all models. Estimates shown were multiplied by 100. (**C**) Line plot showing the correlation between metabolite-predicted age and chronological age for all models before (solid lines) and after (dotted lines) applying a statistical correction to the metabolite-predicted age to remove the age bias (i.e., the systematic overestimation of age in young individuals and underestimation of age in older individuals). (**D** and **E**) Two-dimensional density plots showing the correlation between metabolite-predicted age derived from the Cubist rule–based regression model and chronological age before and after age-bias correction. Observations beyond the *y* axis limits of 30 to 80 not shown. (**F**) MAE for all models with tuned hyperparameter values calculated in the analytical sample after age-bias correction. (**G**) Density plot showing the distribution of MileAge delta (adj.) for all models. [(A) to (G)] See Box 1 for model abbreviations. *N* = 101,359, except for Cubist rules 225k (*N* = 225,212).

Predictive performance estimates in the 90% training (*N* = 91,222 to 91,226) and 10% test sets are shown in table S3. The nested cross-validation mean absolute error (MAE) in the test sets (*N* = 10,133 to 10,137) ranged from 5.31 to 6.36 years, with the support vector regression with a radial basis function (SVM radial) performing best and the multivariate adaptive regression splines (MARS) ensemble performing worst. The root mean square error (RMSE) ranged from 6.60 to 7.58 years. Correlation coefficients between metabolite-predicted and chronological age ranged from 0.36 to 0.59, with coefficient of determination (*R*^2^) values between 0.13 and 0.35. The difference in predictive performance between the training and test sets, i.e., the model’s optimism that may indicate poor generalization to unseen data, was generally low (e.g., MAE_difference_ < 0.15 for 10 of the 17 models). However, certain tree–based models [bagging, random forest, and extreme gradient boosting (XGBoost)] and the *k*-nearest neighbor model overfit the training data (MAE_difference_ = −1.51 to −5.83), with correlation coefficients between metabolite-predicted and chronological age of *r* > 0.8 in the training sets (fig. S40). Figure 2A shows the nested cross-validation MAEs for all models. For comparison, drawing random samples from a uniform distribution between the sample’s minimum and maximum chronological age, i.e., a random prediction model, resulted in a MAE = 9.79. Predicting the sample mean, i.e., a null model, resulted in a MAE = 6.96. Additional plots showing performance measures (MAE, RMSE, *r*, and *R*^2^) in the training and test sets are presented in the Supplementary Materials (figs. S37 to S43). There were moderate to high correlations between the metabolite-predicted age values of the various models (*r* = 0.48 to >0.99) (Fig. 2B). High correlations (*r* > 0.87) among the most accurate models suggest that they capture similar patterns in the data. Omega-3, albumin, and citrate were among the most important contributors to predictive accuracy (fig. S44).

### Age-bias correction

All models overestimated the age of younger individuals and underestimated the age of older individuals (fig. S44). Applying a statistical correction (see Materials and Methods) to the metabolite-predicted age values removed this bias (Fig. 2, C to E, and fig. S45). The predicted age values were within the chronological age range (39 to 70 years) of the sample for most individuals. Across all models, 0.23% (*n* = 238) and 0.96% (*n* = 976) of predictions were below or above the minimum and maximum age, respectively (table S4). More predicted age values were outside the chronological age range (up to 8.27%, *n* = 8382 for a single model) after the age-bias correction. Recalculating the predictive performance estimates after the age-bias correction suggested higher accuracy (MAE = 2.12 to 3.40; Fig. 2F). The overall performance rankings across the models inverted, with the models that originally predicted chronological age best showing reduced accuracy (figs. S46 to S48). The age bias–adjusted metabolomic age (MileAge) delta ranged from −18.94 years younger to 16.05 years older for the Cubist rule–based regression model, with 15.99% (*n* = 16,204) and 15.77% (*n* = 15,989) of the sample having a MileAge delta (adj.) of at least 3.75 years below or above the mean (Fig. 2G and table S5).

### Pseudo-validation of predictive performance and age-bias correction

We further evaluated the predictive model performance of MileAge derived from the Cubist rule–based regression model (trained on *N* = 101,359): (i) in an additional *N* = 129,877 participants at the baseline assessment and (ii) in a subset of *N* = 10,618 participants with repeat assessment data (mean age, 61.97 years; SD, 7.36). The predictive performance was comparable in the additional baseline and follow-up samples (MAE = 5.64 and 5.68, respectively, compared to MAE = 5.42 in the original analytical sample). Applying the same statistical correction to the metabolite-predicted age values that were used for the original sample (i.e., using the same intercept and slope) removed the age bias (fig. S49).

### Associations with health and aging markers

Descriptive statistics for the health and aging markers are shown in table S6. Having an older metabolite-predicted than chronological age was associated with higher frailty index scores across all models (table S7). This extended to the frailty phenotype for all models when comparing individuals with a MileAge delta (adj.) more than one SD above and below the mean and for 12 of the 17 models when comparing the middle of the distribution to individuals with MileAge delta (adj.) values more than one SD below the mean (table S8). For telomere length, we observed a statistically significant group difference for 12 of the 17 models when comparing the tails of the distribution and for 9 of the 17 models when comparing the middle of the distribution to the reference group (table S9). Having an older metabolite-predicted than chronological age was generally associated with worse overall health (tables S10 to S12). An exception to this pattern were the MARS models, for which an older metabolite-predicted than chronological age was associated with longer telomeres and for which there was little evidence of statistically significant differences in health between individuals with MileAge delta (adj.) values in the middle of the distribution and the reference group.

**Fig. 3.**
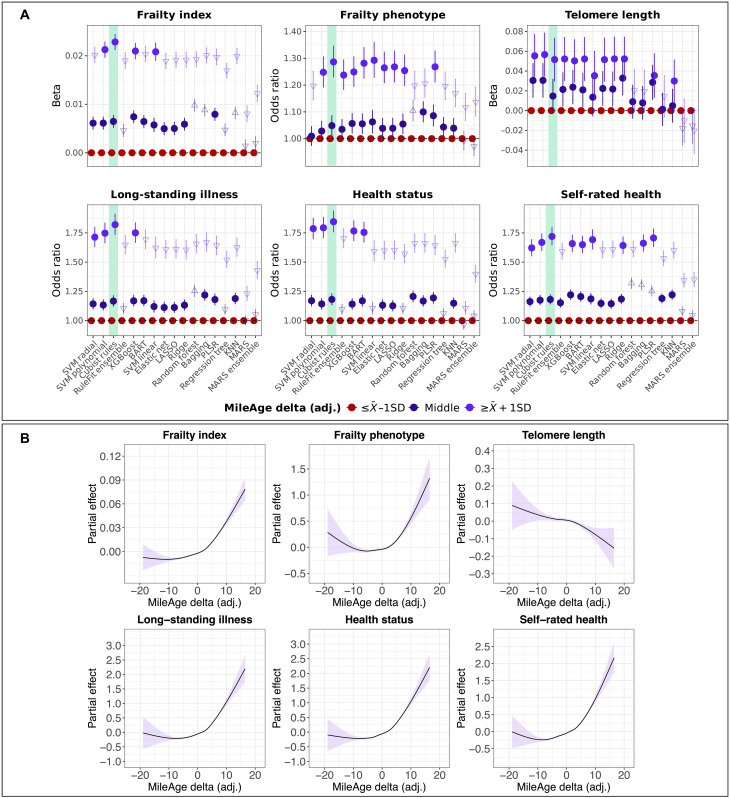
Associations with health and aging markers. (**A**) Associations between MileAge delta (adj.) and health and aging markers for all models, adjusted for chronological age and sex. Reference group: individuals with a MileAge delta (adj.) smaller than one SD below the mean. Upward triangles indicate estimates larger than the estimate for the Cubist rule–based regression model (at the same level), while downward triangles indicate smaller estimates, based on Wald *P* values of <0.05. See Box 1 for model abbreviations. (**B**) Partial effect plots of generalized additive models showing associations between MileAge delta (adj.) derived from the Cubist rule–based regression model and health and aging markers, adjusted for chronological age and sex. The shaded areas correspond to 95% CIs. [(A) and (B)] *N* = 101,029 (frailty index); *N* = 95,061 (frailty phenotype); *N* = 97,964 (telomere length); *N* = 98,750 (long-standing illness); *N* = 98,820 (health status); *N* = 100,789 (self-rated health).

MileAge delta (adj.) derived from the Cubist rule–based regression model was most strongly associated with most health and aging markers (Wald *P* values of <0.05 for up to 13 of the 16 comparisons) (Fig. 3A). Individuals with a MileAge delta (adj.) greater than one SD above the mean had higher frailty index scores than individuals with a MileAge delta (adj.) smaller than one SD below the mean (β = 0.023; 95% CI, 0.021 to 0.024; *P* < 0.001). This group difference was approximately equivalent to an 18.3-year chronological age difference in frailty index scores (β = 0.023 divided by β = 0.001255 derived from a linear model, *y*_frailty index_ ~ *x*_chronological age_). Individuals with an older metabolite-predicted than chronological age were also more likely to be physically frail [odds ratio (OR) = 1.29; 95% CI, 1.23 to 1.35; *P* < 0.001] and had shorter telomeres (β = 0.052; 95% CI, 0.030 to 0.073; *P* < 0.001) equivalent to a 2.2-year chronological age difference in telomere length. Such individuals were also more likely to have a long-standing illness (OR = 1.82; 95% CI, 1.73 to 1.91; *P* < 0.001), poorer health status (OR = 1.85; 95% CI, 1.76 to 1.94; *P* < 0.001), and worse self-rated health (OR = 1.72; 95% CI, 1.65 to 1.80; *P* < 0.001). Generalized additive models showed that positive MileAge delta (adj.) values, indicating accelerated aging, were robustly associated with unfavorable health (Fig. 3B), whereas negative MileAge delta (adj.) values were weakly associated with favorable health. This pattern was consistent across most models (figs. S51 to S56).

### Predicting mortality

**Fig. 4.**
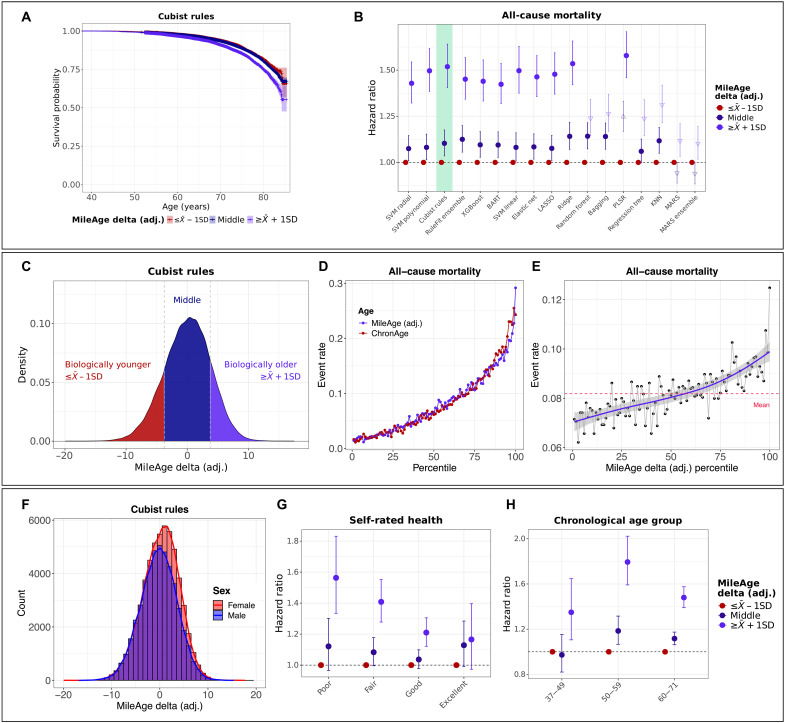
All-cause mortality prediction. (**A**) Kaplan-Meier plot showing survival probabilities for all-cause mortality by MileAge delta (adj.) derived from the Cubist rule–based regression model. Log-rank test *P* value of <0.001. (**B**) HRs and 95% CIs from Cox proportional hazards models by MileAge delta (adj.) for all models, adjusted for chronological age and sex. Age (in years) was used as the underlying time axis. Reference group: individuals with a MileAge delta (adj.) smaller than one SD below the mean. Upward triangles indicate HRs larger than the HR for the Cubist rule–based regression model (at the same level), while downward triangles indicate smaller HRs, based on Wald *P* values of <0.05. See Box 1 for model abbreviations. (**C**) Density plot showing the distribution of MileAge delta (adj.) derived from the Cubist rule–based regression model. (**D** and **E**) All-cause mortality rate by percentile of chronological age, MileAge (adj.) and MileAge delta (adj.) derived from the Cubist rule–based regression model. (**F**) Histogram showing the distribution of MileAge delta (adj.) derived from the Cubist rule–based regression model, stratified by sex. (**G** and **H**) HRs and 95% CIs from Cox proportional hazards models for all-cause mortality by MileAge delta (adj.) derived from the Cubist rule–based regression model, stratified by self-rated health and chronological age group. [(A) and (B)] *N* = 101,274; [(C) and (F)] *N* = 101,359; [(D), (E), and (H)] *N* = 224,996; (G) *N* = 209,554.

In the main analytical sample, the median duration of follow-up of censored individuals was 13.87 years [interquartile range (IQR), 1.37 years], with 1,361,970 person-years of follow-up. There were *N* = 8113 deaths among *N* = 101,274 participants (*N* = 85 missing). MileAge (adj.) was strongly associated with all-cause mortality, comparable to chronological age (Fig. 4, A to E). In the prospective analyses, we examined the age bias–adjusted MileAge delta, and models were adjusted for chronological age and sex, with age (in years) as the underlying time axis (fig. S57 and table S13).

For the Cubist rule–based regression model, a 1-year difference in MileAge delta (adj.) was associated with a hazard ratio (HR) of 1.04 (95% CI, 1.03 to 1.04; *P* < 0.001). Comparing individuals with a MileAge delta (adj.) greater than one SD above and below the mean, the HR was 1.52 (95% CI, 1.41 to 1.64; *P* < 0.001). Individuals with a MileAge delta (adj.) between one SD above and below the mean had a statistically significantly higher mortality risk for 14 of the 17 models (*P* between 0.03 and <0.001) (table S14). Comparing the bottom and top 10% of the MileAge delta (adj.) distribution, instead of one SD above and below the mean, resulted in greater differences (e.g., HR = 1.63; 95% CI, 1.48 to 1.79; *P* < 0.001, for the Cubist model). Individuals in between the tails of the distribution had a higher mortality risk compared to the bottom 10% for 14 of the 17 models (fig. S58 and table S15). When comparing individuals with a positive and negative MileAge delta (adj.), we found that individuals with an older metabolite-predicted than chronological age had a higher mortality risk for all models except the MARS models (fig. S59 and table S16). Modeling the mortality hazard as a spline function of MileAge delta (adj.) suggested that positive values were strongly associated with a higher mortality hazard, while there was little evidence that negative values were associated with a lower mortality hazard (fig. S60).

Females had slightly higher MileAge delta (adj.) values than males (Fig. 4F), a pattern that we observed across all models (fig. S61). The mortality hazard of individuals with a MileAge delta (adj.) (trained on *N* = 225,212 participants) greater than one SD above the mean was elevated in both females (HR = 1.31; 95% CI, 1.21 to 1.42; *P* < 0.001) and males (HR = 1.66; 95% CI, 1.55 to 1.77; *P* < 0.001) (figs. S62 and S63 and table S17). The difference in mortality between individuals with a MileAge delta (adj.) in the middle of the distribution and the reference group was only statistically significant in males (HR = 1.15; 95% CI, 1.09 to 1.21; *P* < 0.001). Stratifying the sample by self-rated health, the mortality hazard of individuals with a MileAge delta (adj.) greater than one SD above the mean was higher in all strata (e.g., HR = 1.56; 95% CI, 1.33 to 1.83; *P* < 0.001, for poor self-rated health) except for individuals with excellent health (Fig. 4G and table S17). The mortality hazard of individuals with a MileAge delta (adj.) greater than one SD above the mean was higher in all age groups (fig. S64, Fig. 4H, and table S17).

### Comparison with other aging markers

**Fig. 5.**
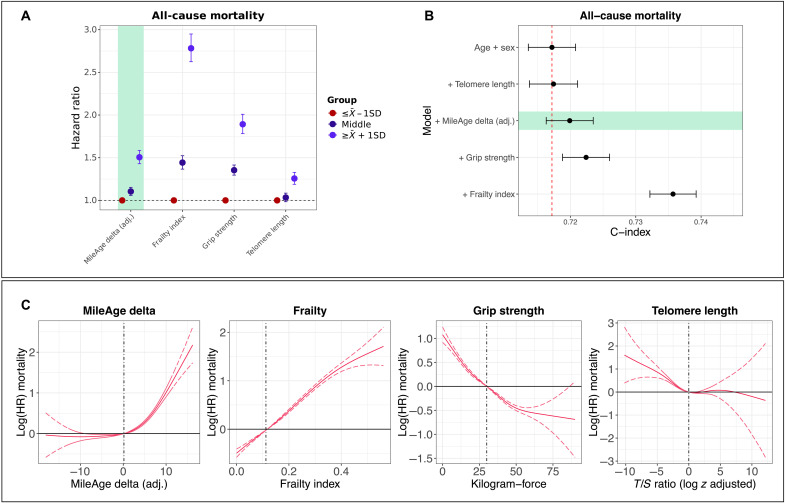
Comparison with other aging markers. (**A**) HRs and 95% CIs from Cox proportional hazards models for all-cause mortality by MileAge delta (adj.), the frailty index, grip strength, and telomere length, adjusted for chronological age and sex. Age (in years) was used as the underlying time axis. Reference group: individuals with a score smaller than one SD below the mean. (**B**) C-index and 95% CIs from Cox proportional hazards models for all-cause mortality for chronological age + sex (the base model) and for each aging marker added separately to the base model. Time (in days) was used as the underlying time axis. (**C**) Log(HRs) and 95% CIs from Cox proportional hazards models for all-cause mortality, adjusted for chronological age and sex. Age (in years) was used as the underlying time axis. A penalized spline function with four degrees of freedom was used. Vertical lines indicate the median of the distribution which represents the reference for interpreting the estimates shown. [(A) to (C)] *N* = 224,996 (MileAge delta); *N* = 224,278 (frailty index); *N* = 224,117 (grip strength); *N* = 217,030 (telomere length).

A comparison of the all-cause mortality hazard associated with MileAge delta (adj.) and other non-metabolomic aging marker subgroups defined by the SD from the mean showed that the largest HR (= 2.78) was observed for the frailty index (Fig. 5A). The smallest HR was observed for telomere length (HR = 1.26) (table S18), with MileAge delta (adj.) (HR = 1.51) and grip strength (HR = 1.89) in between. Adding each aging marker individually as a continuous variable to a base model that included chronological age and sex improved prediction of all-cause mortality, with the best prediction observed for the frailty index [C-index of 0.736 (95% CI, 0.732 to 0.739) versus C-index of 0.717 (95% CI, 0.714 to 0.721) for the base model] (Fig. 5B and table S19). Modeling the mortality hazard as a spline function of the aging markers, to identify potential nonlinear effects, suggested that the all-cause mortality hazard was considerably higher in individuals with an older metabolite-predicted than chronological age. For example, compared to the sample median MileAge delta (adj.), which was equivalent to no difference between metabolite-predicted and chronological age, a MileAge delta (adj.) of 10 was associated with an HR of about 2.7, i.e., a 170% higher morality hazard (Fig. 5C).

MileAge delta was more predictive of all-cause mortality than MetaboAge 2.0 delta (HRs = 1.10 to 1.51 and 0.98 to 1.21, respectively) but less predictive of mortality than MetaboHealth (HRs = 1.60 to 3.34) (fig. S65 and table S20). MileAge delta was more strongly associated with most health and aging markers than MetaboAge 2.0 delta (except for the frailty phenotype and self-rated health), but the magnitude of associations was smaller than those for MetaboHealth (fig. S66 and table S21).

## DISCUSSION

We observed that most metabolite levels varied with chronological age, with considerable evidence of nonlinear associations. Across the machine learning algorithms used to develop aging clocks from plasma metabolites, the nested cross-validation MAE between metabolite-predicted age (MileAge) and chronological age ranged from 5.31 to 6.36 years (*R*^2^ between 0.13 and 0.35). All models overestimated age in younger individuals and underestimated it in older individuals. After applying a statistical correction to remove this age bias, 31.76% of participants had adjusted MileAge delta values of at least 3.75 years, highlighting the potential of metabolomic aging clocks to differentiate between individuals of the same chronological age (*30*). While there was a high degree of consistency across the top performing models, such as support vector regression and certain tree–based ensembles, the aging clock derived from a Cubist rule–based regression model was most strongly associated with most health and aging markers (Wald *P* value of <0.05 for up to 13 of the 16 model comparisons). Across most models, individuals with an older metabolite-predicted than chronological age, indicating accelerated aging, were frailer, had shorter telomeres, were more likely to have a chronic illness, rated their health worse, and had a higher mortality risk.

Several previous studies have developed aging clocks trained on chronological age from metabolomics data. The seminal study by Menni *et al.* (*24*) derived a metabolite age score as a linear combination of 22 plasma metabolites in 6055 twins, achieving an *R*^2^ of 59% and an HR for all-cause mortality of HR = 1.08 per year. Hertel *et al.* (*26*) tested a multivariable linear regression and a fractional polynomial model in 3611 participants, with the latter achieving a correlation between metabolite-predicted and chronological age of *r* = 0.53 (RMSE = 11.19) in men and *r* = 0.61 (RMSE = 10.37) in women. Their metabolomic aging clock included 59 urinary metabolites and was predictive of all-cause mortality (HR = 1.24 per SD). Robinson *et al.* (*27*) developed aging clocks using elastic net models in 2238 participants, with correlations between metabolite-predicted and chronological age of *r* = 0.45 (MAE = 4.17) to *r* = 0.83 (MAE = 6.49). van den Akker *et al.* (*28*) developed an aging clock, MetaboAge, from 56 metabolites in 18,716 participants from 24 community and hospital-based cohorts using a linear regression model, achieving a correlation of *r* = 0.65 and a median absolute error of 7.3. An independent external validation of this clock observed a notably lower correlation of *r* = 0.21 (*17*). Macdonald-Dunlop *et al.* (*17*) also developed an aging clock from NMR metabolomics data (81 of the 86 metabolites selected) in 1643 individuals, which achieved a correlation of *r* = 0.74 but failed to replicate in a validation cohort, and another clock from mass spectrometry metabolomics (181 of the 682 metabolites selected) in 861 individuals, which achieved a correlation of *r* = 0.81. An updated aging clock, MetaboAge 2.0, in the BBMRI-NL data that included 65 metabolites achieved *R*^2^ values of 0.451 and 0.449 for a linear regression and elastic net model, respectively (*32*). While previous studies have primarily used individual algorithms or explored a small selection of algorithms for developing aging clocks (*33*, *34*), our comprehensive study benchmarked a wide range of models under consistent conditions in one of the largest metabolomics datasets available globally.

Metabolomic aging clocks trained on chronological age are generally less accurate than aging clocks derived from other types of omics data (*15*). Nevertheless, our most predictive model (MAE = 5.31 years) had a similar accuracy as a deep learning aging clock (MAE = 5.68) derived from blood markers, biometrics, and sex in the China Health and Retirement Longitudinal Study. An elastic net model in the same data achieved a MAE = 6.19 years (*35*), while the elastic net model in our study had a lower MAE = 5.70, likely due to our larger sample size. While a discussion of how well different types of aging clocks predict health outcomes is beyond the scope of this study, the most widely tested epigenetic aging clocks that were trained on chronological age are weak predictors of mortality (*36*, *37*). Second-generation aging clocks are more predictive of mortality. For example, a 1-year increase in PhenoAge, which was trained on physiological dysregulation, was associated with a 9% increase in mortality risk. Its epigenetic derivation, DNAm PhenoAge, was associated with a 4.5% increase in all-cause mortality (*38*). GrimAge, another popular mortality risk predictor that incorporates chronological age, sex, and eight DNAm surrogate markers (seven for plasma proteins and one for smoking pack-years), strongly predicts mortality and age-related diseases (*12*). Its second iteration, GrimAge2, which integrates two additional surrogate markers of C-reactive protein and hemoglobin A1C, outperforms GrimAge at predicting morbidity and mortality (*39*). Another epigenetic mortality predictor, bAge, that includes the original GrimAge DNAm surrogate markers, chronological age, sex, and an additional 109 DNAm surrogate markers of proteins also outperforms GrimAge at mortality prediction (*40*). A recent clock developed from circulating biomarkers and trained to predict mortality in the UK Biobank (*N* = 307,000) using an elastic net Cox model (*41*) yielded a 9.2% relative increase in prediction compared to the PhenoAge model. These findings suggest that certain omics clocks capture physiologically relevant signals more, while first-generation epigenetic clocks remain the best predictors of chronological time (*15*). A direct comparison in our study suggested that MileAge delta was more strongly associated with mortality and most health and aging markers than MetaboAge 2.0 delta, another clock trained on chronological age. It was, however, not as strongly associated with mortality and health as MetaboHealth, a metabolomic profile of mortality risk, aligning with previous findings (*34*) and other second-generation aging clocks.

Although chronological age prediction in itself is used in fields such as forensics (*42*), it is less useful in population health and geroscience, given that chronological age is non-modifiable (*43*). A perfect prediction model would merely tell us about chronological, not biological, age (*7*). It is the difference between predicted and chronological age (e.g., MileAge delta) that serves as an indicator of accelerated or decelerated aging. A less accurate chronological age prediction does not necessarily indicate a worse model for predicting age-related health outcomes (*30*); hence, we also tested algorithms that were expected to perform less well at predicting chronological age (e.g., linear models). Prior studies suggested that age estimates derived from physical activity (*44*) or epigenetic data (*31*) with higher predictive accuracy of chronological age were less predictive of all-cause mortality. Our study showed that the models that were most predictive of chronological age were generally also more strongly associated with health and mortality, although the pattern of predictive accuracy largely inverted after correcting for the age bias.

Although previous studies have partially demonstrated the utility of both linear and more complex nonlinear models (*33*, *34*), our study advances the field by systematically comparing a broader array of algorithms within the same metabolomics dataset, emphasizing the importance of model selection for age estimation. While we have used this approach to train models on chronological age, future research could apply a similar approach to developing aging clocks trained on other health outcomes.

We report several additional analyses, including variable importance scores and benchmarking against other aging markers, for MileAge derived from the Cubist rule–based regression model, which was most strongly associated with most health and aging markers. However, several conclusions can be drawn across most of the machine learning algorithms tested. First, the wide range of the MileAge delta values quantifies the latent patterns in the metabolomics data not captured by chronological age and suggests that our aging clocks track (past) rate of biological aging for people of the same chronological age. Second, the associations between most aging clocks, health and aging markers, and mortality demonstrate that these clocks capture biologically relevant information, which may find applications in health tracking and nutrition or in clinical trials (e.g., sample stratification). A key finding across most aging clock models tested here was that associations with health and mortality were stronger in individuals with an older metabolite-predicted than chronological age and less so in individuals with a younger predicted than chronological age. This nonlinear relationship between MileAge delta and the health and aging markers and mortality was observed across algorithms. The lack of (robust) association between negative MileAge delta scores, i.e., having a lower metabolite-predicted than chronological age, aligns with findings for other metabolomics-based risk scores (*20*) and highlights the need for careful interpretation in risk stratification. Although accelerated metabolomic aging correlates with poor health and higher mortality risk, decelerated aging does not equivalently translate to better health outcomes, suggesting that certain metabolomics-based risk scores should (for now) primarily be used to identify high-risk individuals.

Our study presents a comprehensive comparison of machine learning algorithms for developing aging clocks from metabolomics data. Several of the algorithms included in our comparison, including those that were most predictive of chronological age, mortality, and health, allowed for nonlinear relationships in the data, which were often not considered in previous studies (*18*). A literature review found that most molecular aging clocks were developed using linear models with regularization, whereas few used nonlinear models (*45*). Although it has been asserted that age-related physiological markers are linearly associated with age (*44*), we have shown here and in previous studies (*4*–*6*) that this does not apply to many biological markers. Although algorithms that allowed for nonlinear patterns in the data generally achieved superior performance at predicting age, regularized linear regression models such as elastic net remained competitive, especially given their lower computational demands. To enable comparability between studies, we provide a comprehensive set of statistical estimates and developed our aging clock using metabolites measured in absolute concentrations instead of relative to other measures. Although it may be argued that quantification of a smaller number of metabolites would, in principle, be more feasible and convenient in clinical practice, all metabolites included in our model can be quantified from a single assay with minimal sample preparation required (*46*).

Our study has certain limitations. Some algorithms that have previously been used to develop biological aging clocks, for example, deep learning (*47*), or approaches combining Hilbert–Schmidt independence criterion (HSIC) least absolute shrinkage and selection operator (LASSO) feature selection with nonlinear support vector regression (*48*) were not tested and could be explored in future studies. Our aging clock provides a “systems level” indicator of age-related changes in metabolites; future clocks may be developed at the tissue or cellular level. Plasma samples may differ from other body fluids, e.g., serum, urine, or cerebrospinal fluid. The metabolite coverage of the Nightingale Health platform is lipid focused and mostly covers larger molecules, while there are potentially over 217,000 endogenous and exogenous molecules (*49*). Nevertheless, this platform enables robust assessment of these metabolites in a single experiment (*46*). Although more complex aging clocks could be developed using technologies with wider metabolite coverage, prior analyses suggested that a majority of metabolites associated with age were related to lipid and amino acid pathways, both of which were included here (*24*). As in previous studies, we observed a systematic overestimation of age in younger individuals and underestimation of age in older individuals (*7*). This bias is neither data nor method specific and may be explained by regression to the mean (*50*) of the training data (*51*). To account for this bias, we have adjusted the metabolite-predicted age and included chronological age as a covariate in the health association analyses (*45*). Longitudinal aging metrics may be more robustly associated with certain health outcomes than cross-sectional aging metrics, e.g., with physical and cognitive decline but not, for example, multimorbidity (*52*). Future research should develop metabolomic aging clocks from longitudinal data. Pseudo-validation of MileAge in *N* = 129,877 additional participants at the baseline assessment and *N* = 10,618 participants with repeat assessment data (mean age, 61.97 years; SD, 7.36) suggested similar predictive performance. Nevertheless, the lack of independent data for external validation is a limitation. Future research should focus on validating MileAge in independent cohorts to establish the generalizability of MileAge and the age-bias correction beyond the UK Biobank and to ensure robustness across different populations.

Metabolomic aging clocks derived from multiple machine learning algorithms were robustly associated with health and aging markers as well as mortality. The aging clock derived from the Cubist rule–based regression model was overall most strongly associated with health and aging markers. Individuals with metabolite-predicted age (MileAge) values greater than their chronological age, indicating accelerated aging, were frailer, had shorter telomeres, were more likely to have a chronic illness, rated their health worse, and had a higher mortality risk. Aging clocks hold substantial promise for research on life span and health span extension, as they provide an aging biomarker that is potentially modifiable. These clocks may also help identify health risks before clinical symptoms emerge. As such, biological aging clocks may contribute to health risk assessments, complementing clinical biomarkers. However, the utility of aging clocks is not limited to risk stratification, but it also provides an intuitive, year-based metric for health tracking that may help individuals proactively engage with their health.

## MATERIALS AND METHODS

### Study population

The UK Biobank is a prospective health study of over 500,000 UK residents aged 37 to 73, recruited between 2006 and 2010. Individuals registered with the UK National Health Service (NHS) and living within a 25-mile (~40-km) radius of 1 of the 22 assessment centers were invited to participate (*29*). Participants provided data on their sociodemographic characteristics, health behaviors, and medical history; underwent physical examinations; and had blood and urine samples taken. A subset of participants (*N* = 20,344) living within a 35-km radius of the assessment center at Stockport, England, completed a repeat assessment between 2012 and 2013. There is extensive record linkage, for example, with national death registries, hospital inpatient records, and primary care data. Ethical approval for the UK Biobank study has been granted by the National Information Governance Board for Health and Social Care and the NHS North West Multicentre Research Ethics Committee (11/NW/0382). No project-specific ethical approval is needed.

### Metabolomic biomarker quantification

NMR spectroscopy was used to quantify metabolomic biomarkers in non-fasting blood plasma samples collected at the baseline assessment. The Nightingale Health platform ascertains 168 circulating metabolites using a high-throughput standardized protocol for sample quality control, preparation, data storage, and automated analyses (*46*). These metabolites span multiple pathways, including lipoprotein lipids, circulating fatty acids, and fatty acid compositions, as well as low–molecular weight metabolites, such as amino acids, ketone bodies, and glycolysis metabolites. Most measures are highly correlated (*r* > 0.9) with routine clinical chemistry assays (*46*). For further details on sample preparation and quality control procedures, see https://biobank.ndph.ox.ac.uk/ukb/ukb/docs/nmrm_companion_doc.pdf. We used the first release of metabolomics data (March 2021) on a random subset of *N* = 118,019 participants for the main analysis. We subsequently obtained the second release of metabolomics data (July 2023) that included *N* = 274,315 participants and performed removal of technical variation in these data using the “ukbnmr” R package (algorithm v2) (*53*).

### Chronological age

We estimated participants’ chronological age at baseline and the repeat assessment using their birth year (UK Biobank data field 34) and birth month (field 52), along with the dates of attendance at the assessment centers (fields 53.0.0 and 53.1.0). To derive more precise values, we assigned each participant a random day within their birth month, considering the correct number of days per month and adjusting for leap years when necessary. Chronological age was calculated by computing the number of days between the assigned date of birth and the assessment date, divided by 365.25, to convert it into decimal years. A similar approach was implemented by Argentieri *et al.* (*54*).

### Machine learning

We evaluated 17 machine learning algorithms, including regularized linear regression, latent variable modeling, instance-based learning, nonparametric regression, kernel-based methods, tree-based models, rule-based models, and ensemble methods (Box 1). To internally validate each algorithm in predicting chronological age from plasma metabolites, we implemented 10 × 5 nested cross-validation (Fig. 1A). Nested cross-validation is preferred for internal validation as it provides more accurate error estimation than other approaches (*55*). We split the data into 10 folds of equal size, to which individuals were randomly allocated while preserving the chronological age distribution of the analytical sample (Fig. 1B).

For each iteration of the outer loop of the nested cross-validation, 9 of the 10 folds served as the training set, and the 10th fold served as the test set. The 90% training sets were further divided into five equal size sets, and we performed fivefold cross validation to empirically identify, for each algorithm, the hyperparameter combination that resulted in the lowest cross-validation MAE. Tuning grids were designed using a maximum entropy space-filling design. The size of each tuning grid was determined by the number of available hyperparameters, type of hyperparameter (continuous, discrete, or categorical), and computational constraints. We tested up to 10 values for each hyperparameter, resulting in tuning grid sizes between 10 (for Ridge regression) and 3125 (for XGBoost). Further details, including preprocessing requirements, are available in table S1. The model specifications with the lowest fivefold cross-validation MAE were subsequently fit in the 90% training sets, and performance was assessed by calculating the MAE, RMSE, Pearson *r*, and the *R*^2^ in the 10% test sets. We also examined the discrepancy in predictive performance between the training and test sets, extrapolation beyond the chronological age range in the data, and the computing hours required for hyperparameter tuning for each model.

Box 1 Overview of the machine learning algorithms used in this study.

Ridge regression: Linear regression model with a penalty term (L_2_ regularization) to shrink the magnitude of coefficient estimates toward zero (*63*).

Least absolute shrinkage and selection operator (LASSO): Linear regression model with a penalty term (L_1_ regularization) to shrink the magnitude of coefficient estimates toward zero. This technique can result in sparse models as variable selection is performed by reducing some coefficient estimates exactly equal to zero (*64*).

Elastic net: Linear regression model that combines the L_1_ and L_2_ penalty terms of LASSO and Ridge regression. This technique reduces the magnitude of some coefficient estimates toward zero and can perform variable selection by reducing some coefficient estimates exactly equal to zero (*65*).

Partial least squares regression (PLSR): Latent variable model that extracts a set of latent factors that best explain the covariance between the predictors and outcome. These factors are then used as predictors in a linear regression model (*66*).

K-nearest neighbors (KNN): Instance-based learning model that uses the weighted average outcome of the *k* nearest data points to make predictions. In this context, “nearest” is determined by a distance metric such as the Minkowski distance (*67*).

Multivariate adaptive regression splines (MARS): Nonparametric regression model that creates piecewise linear approximations of the relationship between the predictors and outcome. This technique can model nonlinear associations and interactions between the predictors (*68*).

MARS ensemble: Ensemble method that combines the predictions of multiple MARS models to improve the predictive accuracy and stability of the model (*69*).

Support vector regression (SVR): Kernel-based method that seeks to identify a hyperplane that best models the relationships between the predictors and outcome. This variation of the support vector machine algorithm (*70*) was adapted for regression and can use a range of kernels to transform the data to a higher-dimensional space, allowing for complex nonlinear associations (*71*). We tested linear, polynomial, and radial basis function kernels.

Regression tree: Technique that models the relationship between the predictors and outcome by creating a tree-like structure of decision rules based on values of the predictors. Decision trees are interpretable and can incorporate nonlinear relationships and higher-order interactions in the data (*72*).

Bagging: Ensemble method that combines the predictions of multiple regression trees. Bootstrapped aggregating (“bagging”) involves training regression trees on multiple, randomly selected (“bootstrapped”) samples of the data. Bagging can improve the stability and accuracy of decision tree models (*73*).

Random forest: Ensemble method that combines predictions of multiple regression trees. Each tree is trained on a random subset of the data and, at each split, a subset of predictors, reducing the correlation between decision trees in the ensemble (*74*).

Extreme gradient boosting (XGBoost): Ensemble method that builds decision trees sequentially by implementing a gradient descent algorithm that seeks to minimize errors from previous models while increasing the influence (“boosting”) of highly predictive models. More complex models are penalized through L_1_ and L_2_ regularization to avoid overfitting (*75*).

Bayesian additive regression trees (BART): Ensemble method that uses Bayesian techniques to iteratively construct and update multiple decision trees. Regularization priors force each tree to explain only a subset of the relationship between the predictors and outcome, thereby preventing overfitting (*76*).

Cubist rule–based regression: Ensemble model that derives rules from decision trees and fits linear regression models for the subset of the data defined by each rule. The model incorporates boosting techniques and may adjust predictions based on *k*-nearest neighbors (*77*, *78*).

RuleFit ensemble: Ensemble method that uses a tree-based model (XGBoost) to predict an outcome and subsequently derives rules. LASSO is then used to select the most predictive rules, resulting in a sparse linear model (*79*).

### Metabolomic aging clocks

Individual-level age predictions were obtained by aggregating the predictions from the 10 test sets of the outer loop. MileAge delta was defined as the difference between metabolite-predicted and chronological age. Positive values indicated an older predicted than chronological age, and negative values indicated a younger predicted age. Given that aging clocks overestimate age in younger individuals and underestimate age in older individuals, we regressed metabolite-predicted age (MileAge) on chronological age and used the resulting intercept (β) and slope coefficient (α) estimates to apply a statistical correction to the age prediction: MileAge (age bias adjusted)=MileAge+[Age−(α×Age+β)] (*56*).

### Health, aging markers, and mortality

We assessed associations between MileAge delta (adj.) and various health indicators: long-standing illness, disability or infirmity (yes/no), self-rated health (poor, fair, good, or excellent), and overall health status (unhealthy/healthy) derived from 81 cancer and 443 non-cancer illnesses (*1*, *57*). Next, we examined associations with the frailty phenotype and frailty index (*58*). The frailty phenotype summarizes data on weight loss, exhaustion, physical activity, walking speed, and grip strength. The frailty index was derived from 49 variables obtained at the baseline assessment, including cardiometabolic, cranial, immunological, musculoskeletal, respiratory and sensory traits, well-being, infirmity, cancer, and pain. We also tested associations between MileAge delta (adj.) and telomere length, measured using a validated quantitative polymerase chain reaction assay that expresses telomere length as the ratio of the telomere repeat copy number (*T*) relative to a single-copy gene (*S*) that encodes hemoglobin subunit beta. *T*/*S* ratio is proportional to an individual’s average telomere length (*59*). Last, we examined prospective associations with all-cause mortality. The date of death was obtained through linkage with national death registries, NHS Digital (England and Wales) and the NHS Central Register (Scotland). The censoring date was 30 November 2022.

### Exclusion criteria

Women who were pregnant or unsure that they were pregnant were excluded from the analysis given that their metabolite profiles likely changed during pregnancy. Participants for whom their genetic and self-reported sex did not match were also excluded as this may indicate poor data quality. We also excluded individuals with missing metabolite data and those with outlier metabolite values, defined as values 4× the IQR above or below the median (*60*), to minimize the impact of potentially anomalous observations.

### Statistical analyses

Data processing, analysis and visualization were performed in R (version 4.3.0). Sample characteristics were summarized using means and SDs or counts and percentages. Generalized additive models were used to explore the relationship between chronological age and metabolite levels. We further conducted metabolome-wide association analyses of chronological age and all-cause mortality to identify metabolites that were statistically significantly associated with chronological age and mortality (at *P* < 0.05/168). Correlations between the metabolite-predicted age derived from each machine learning model were estimated using Pearson’s *r*.

Cross-sectional associations between MileAge delta (adj.) and the frailty index and telomere length were estimated using ordinary least squares regression. Associations between MileAge delta and long-standing illness and overall health status were estimated using logistic regression. Association between MileAge delta and the frailty phenotype and self-rated health were estimated using ordinal logistic regression. For each health indicator, higher values corresponded to worse health. For the health association analyses, we fitted minimally adjusted models that included chronological age and sex as covariates. For the prospective analyses of all-cause mortality, we calculated person-years of follow-up and the median duration of follow-up of censored individuals. Survival probabilities by MileAge delta were estimated using the Kaplan-Meier method (*61*), and we calculated log-rank *P* values. HRs and 95% CIs were estimated using Cox proportional hazards models (*62*). Age in years was used as the underlying time axis, with age 40 as the start of follow-up. Across both cross-sectional and prospective analyses, we defined MileAge delta subgroups by their SD from the mean. Individuals with a MileAge delta equal to or smaller than one SD below the mean were the reference group. To discern how this analytical decision impacted results and to enable comparability with other studies, we also report associations with all-cause mortality for all models with subgroups defined by the bottom and top 10% of the distribution as well as negative and positive values. Statistically significant differences in associations estimates, such as HRs, between the aging clocks derived from different algorithms were identified using Wald test *P* values of <0.05. Last, we used generalized additive models and penalized spline functions to explore the relationship between MileAge delta as a continuous variable and health and aging markers and mortality, respectively.

For the Cubist rule–based regression model, which across most analyses was most strongly associated with health and aging markers, we performed pseudo-validation of its predictive performance and the age-bias correction procedure (i) in *N* = 129,877 participants from the second release of baseline metabolomics data and (ii) in a subset of *N* = 10,618 participants with repeat assessment data, none of whom were included in the original analytical sample used for model training. We subsequently retrained MileAge using the combined metabolomics data from the first and second release (*N* = 225,212 after quality control and exclusions described above) and performed the following analyses: We calculated variable importance scores to identify metabolites that strongly contributed to MileAge. We explored associations between MileAge delta and all-cause mortality stratified by sex, self-rated health, and chronological age group (37 to 49, 50 to 59, and 60 to 71 years). We also assessed the performance of MileAge delta by benchmarking it against other non-metabolomic aging markers: (i) We estimated the HR for all-cause mortality by MileAge delta and other aging marker (grip strength, telomere length, and the frailty index) subgroups defined by SD from the mean, adjusted for chronological age and sex; (ii) we calculated the C-index and 95% CIs for chronological age + sex (as the base model) and for each aging marker added separately to the model, with time (in days) since the baseline assessment as the underlying time axis. Last, we compared the performance of MileAge delta at predicting mortality and its associations with the health and aging markers against two existing metabolomic scores: MetaboAge 2.0 (*32*), a metabolomic aging clock trained on chronological age, and MetaboHealth (*21*), a metabolomic profile of mortality risk.
