# Supplementary material for: Metabolomic age (MileAge) predicts health and life span: A comparison of multiple machine learning algorithms
Source: Sci Adv. 2024 Dec 18;10(51):eadp3743. doi: 10.1126/sciadv.adp3743 (PMC11654675; doi:10.1126/sciadv.adp3743)
Supplement: Supplementary file 1 — Tables S1 to S21 Figs. S1 to S66 Legends for data S1 and S2 [file sciadv.adp3743_sm.pdf]

Science Advances
AAAS

Supplementary Materials for

Metabolomic age (MileAge) predicts health and life span: A comparison of multiple machine learning algorithms

Julian Mutz *et al.*

Corresponding author: Julian Mutz, julian.mutz@gmail.com

Sci. Adv. **10**, eadp3743 (2024)
DOI: 10.1126/sciadv.adp3743

The PDF file includes:

Tables S1 to S21
Figs. S1 to S66
Legends for data S1 and S2

Other Supplementary Material for this manuscript includes the following:

Data S1 and S2

1. Hyperparameter tuning

Table S1. Details hyperparameter tuning

		Pre-processing			Hyperparameters			
Algorithm	Engine	Normalize	Zero variance	None	Fixed	Tuned	Grid size	Racing
Ridge	glmnet	×			1	1	10	
LASSO	glmnet	×			1	1	10	
Elastic net	glmnet	×			2		100	
PLSR	mixOmics	×	×		2		40	
KNN	kknn	×			3		125	×
MARS	earth			×	3		48	×
MARS ensemble	earth			×	3	1	48	
SVM linear	kernlab	×			2		100	
SVM polynomial	kernlab	×			4		375	×
SVM radial	kernlab	×			3		125	×
Regression tree	rpart			×	3		1000	
Bagging	rpart			×	3	1	125	×
Random forest	ranger			×	3		125	×
XGBoost	xgboost			×	5	3	3125	×
BART	dbarts			×	3	1	125	×
Cubist rules	Cubist			×	3		125	×
RuleFit ensemble	xrf			×	5	3	3125	×

Note: See Box 1 for model abbreviations.

2. UK Biobank data fields

Table S2. UK Biobank data

Data field IDs	Variable name
Sample characteristics	
31	Sex
53	Date of attending assessment centre
54	UK Biobank assessment centre
74	Fasting time
738	Average total household income before tax
2724	Had menopause
3140	Pregnant
6138[1]	Qualifications
6141[1]	How are people in household related to participant
21000[1]	Ethnic background
21003	Age when attended assessment centre
22001	Genetic sex
20116	Smoking status
23104	Body mass index (BMI)
Health indicators	
134[1]	Number of self-reported cancers
135[1]	Number of self-reported non-cancer illnesses
2178	Overall health rating
2188	Long-standing illness, disability of infirmity
20001[1]	Cancer code, self-reported
20002[1]	Non-cancer illness code, self-reported
40000	Date of death
22192	Z-adjusted T/S log

Note: [1]These variables were further processed and categories used in the present study are shown in the main body of the text.

Details on the data fields used to derive the frailty index and frailty phenotype can be found in (*58*).

Details on the data fields for the Nightingale Health nuclear magnetic resonance (NMR) spectroscopy metabolomic biomarkers can be found in the UK Biobank showcase:

https://biobank.ndph.ox.ac.uk/showcase/label.cgi?id=220

3. Sample flowchart

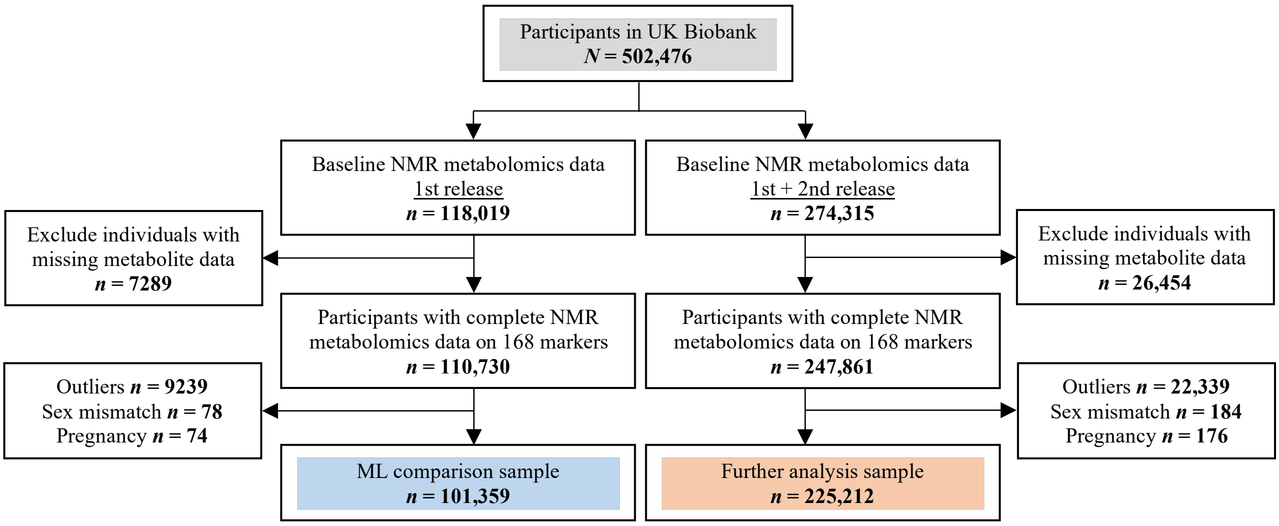

Figure S1. Study sample flowchart. Outliers were defined as metabolite values 4×IQR above or below the median. NMR = nuclear magnetic resonance; IQR = interquartile range.

4. Distributions of metabolite levels by chronological age

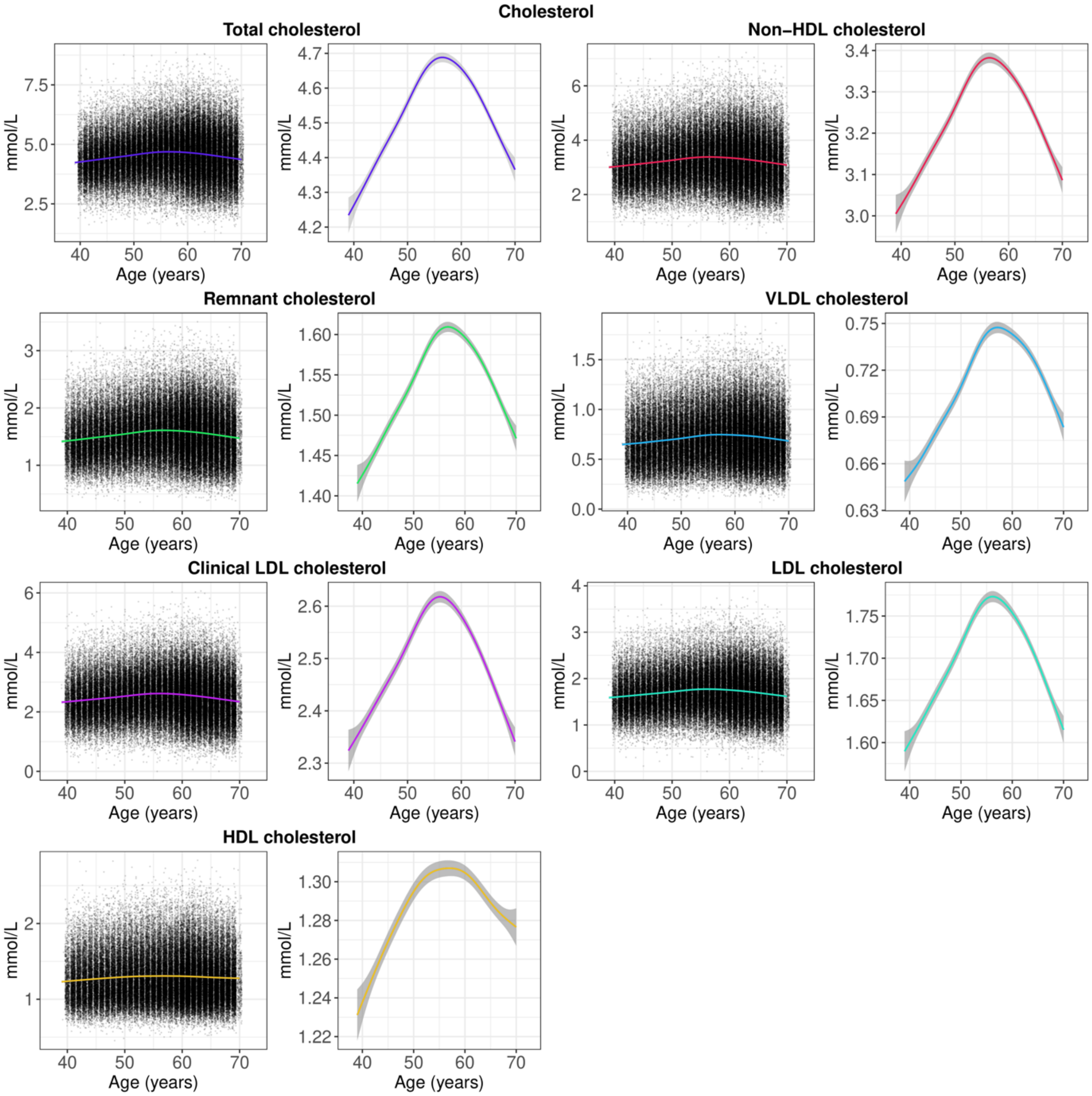

Figure S2. Distribution of cholesterol levels by chronological age, showing scatter plots for all observations and smooth curves (note the difference in y-axis scale). The smooth curves were estimated using generalised additive models, with shaded areas corresponding to 95% confidence intervals. HDL = high-density lipoprotein; VLDL = very low-density lipoprotein; LDL = low-density lipoprotein. *N* = 101,359.

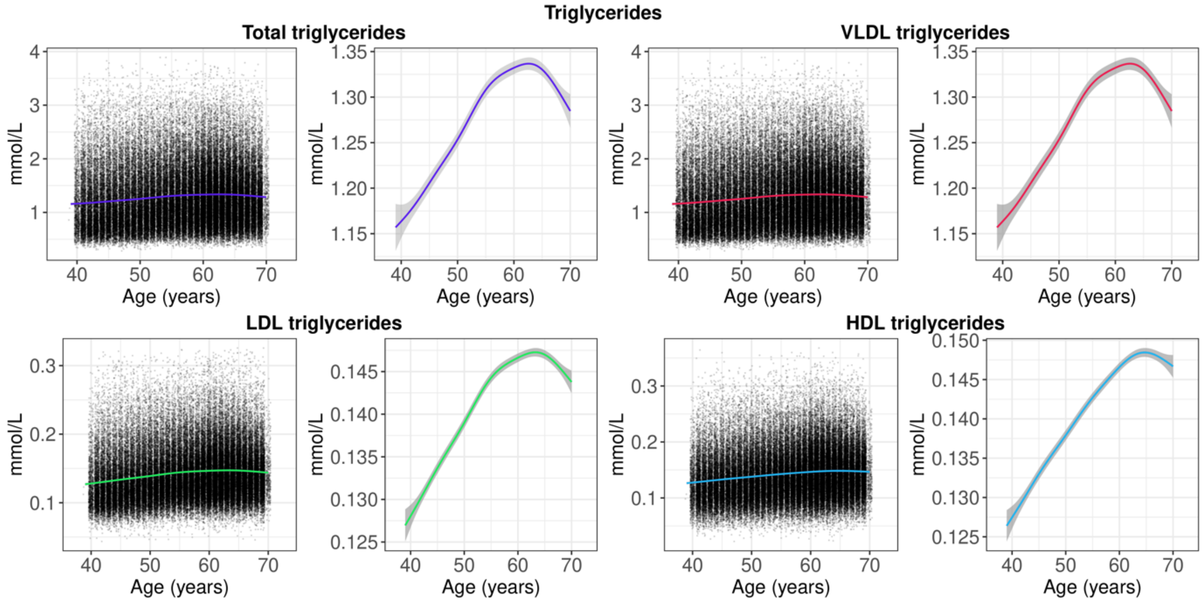

Figure S3. Distribution of triglyceride levels by chronological age, showing scatter plots for all observations and smooth curves (note the difference in y-axis scale). The smooth curves were estimated using generalised additive models, with shaded areas corresponding to 95% confidence intervals. VLDL = very low-density lipoprotein; LDL = low-density lipoprotein; HDL = high-density lipoprotein. *N* = 101,359.

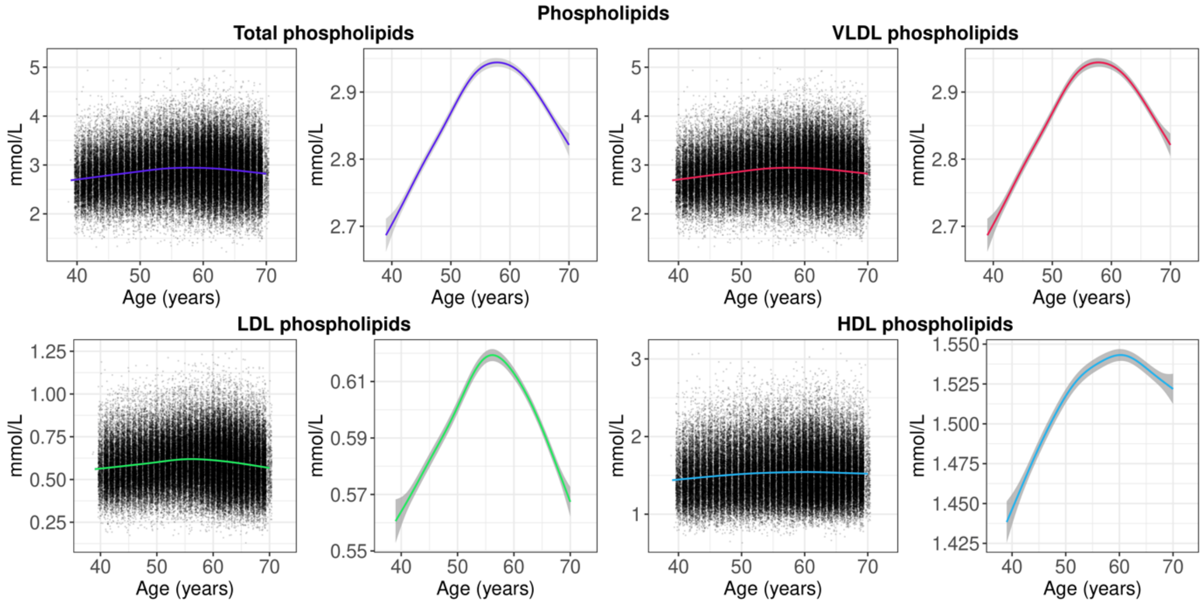

Figure S4. Distribution of phospholipid levels by chronological age, showing scatter plots for all observations and smooth curves (note the difference in y-axis scale). The smooth curves were estimated using generalised additive models, with shaded areas corresponding to 95% confidence intervals. VLDL = very low-density lipoprotein; LDL = low-density lipoprotein; HDL = high-density lipoprotein. N = 101,359.

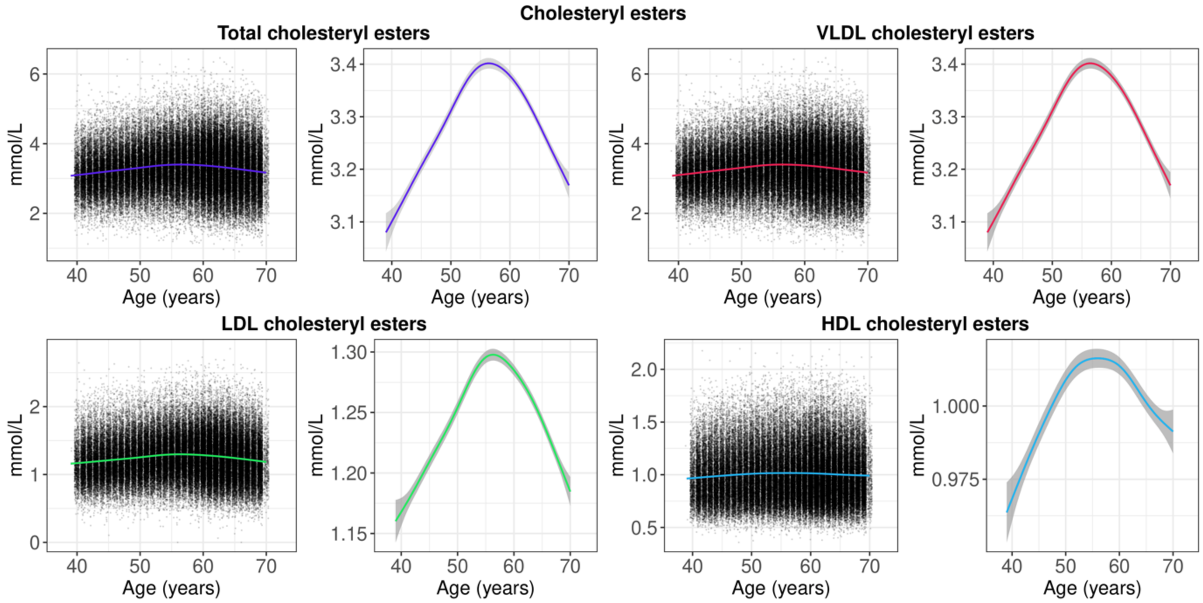

Figure S5. Distribution of cholesteryl ester levels by chronological age, showing scatter plots for all observations and smooth curves (note the difference in y-axis scale). The smooth curves were estimated using generalised additive models, with shaded areas corresponding to 95% confidence intervals. VLDL = very low-density lipoprotein; LDL = low-density lipoprotein; HDL = high-density lipoprotein. *N* = 101,359.

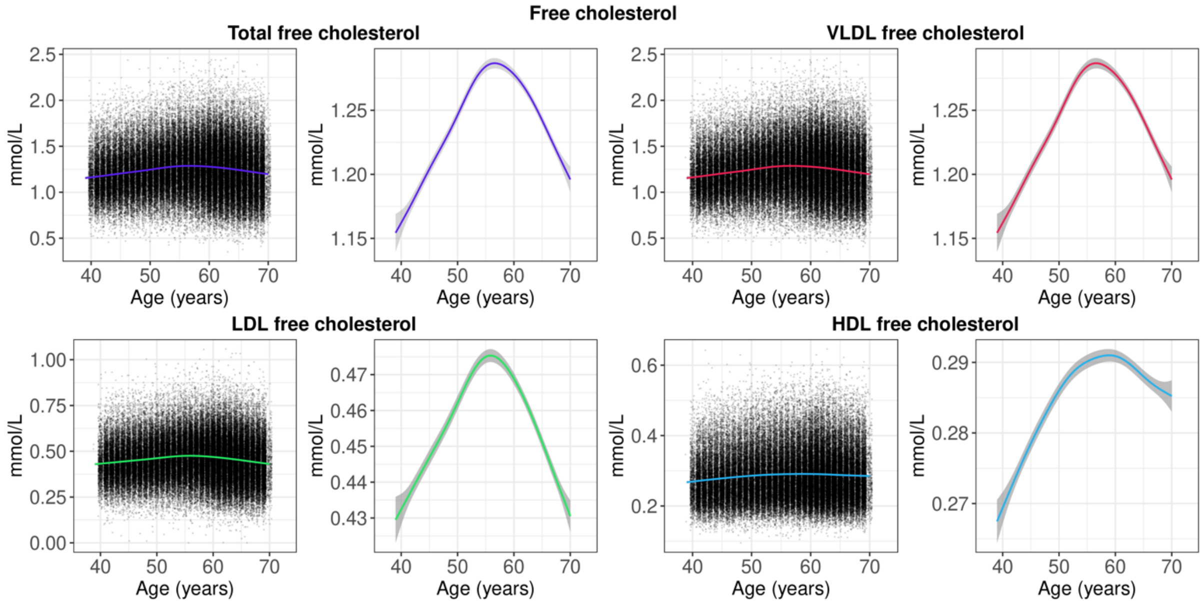

Figure S6. Distribution of free cholesterol levels by chronological age, showing scatter plots for all observations and smooth curves (note the difference in y-axis scale). The smooth curves were estimated using generalised additive models, with shaded areas corresponding to 95% confidence intervals. VLDL = very low-density lipoprotein; LDL = low-density lipoprotein; HDL = high-density lipoprotein. *N* = 101,359.

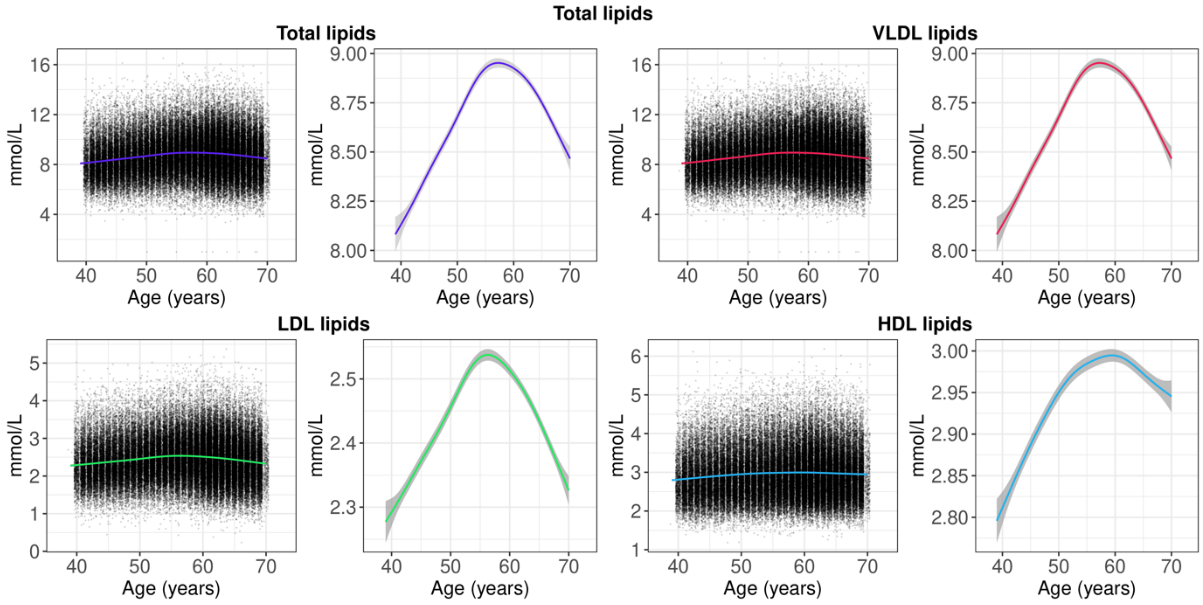

Figure S7. Distribution of total lipid levels by chronological age, showing scatter plots for all observations and smooth curves (note the difference in y-axis scale). The smooth curves were estimated using generalised additive models, with shaded areas corresponding to 95% confidence intervals. VLDL = very low-density lipoprotein; LDL = low-density lipoprotein; HDL = high-density lipoprotein. *N* = 101,359.

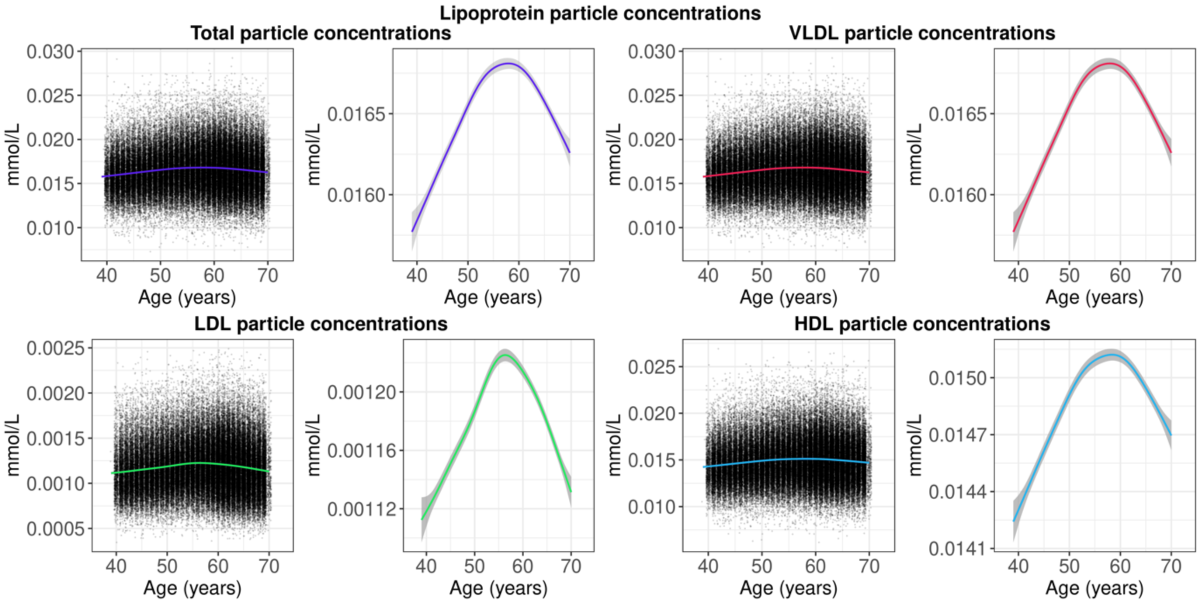

Figure S8. Distribution of lipoprotein particle concentrations by chronological age, showing scatter plots for all observations and smooth curves (note the difference in y-axis scale). The smooth curves were estimated using generalised additive models, with shaded areas corresponding to 95% confidence intervals. VLDL = very low-density lipoprotein; LDL = low-density lipoprotein; HDL = high-density lipoprotein. N = 101,359.

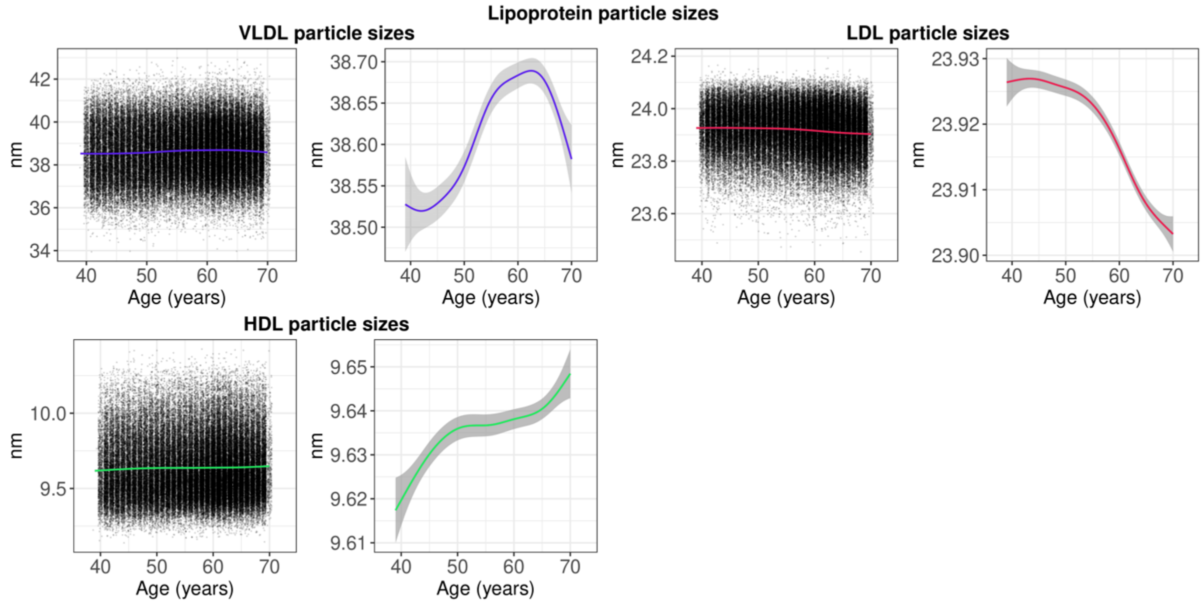

Figure S9. Distribution of lipoprotein particle sizes by chronological age, showing scatter plots for all observations and smooth curves (note the difference in y-axis scale). The smooth curves were estimated using generalised additive models, with shaded areas corresponding to 95% confidence intervals. VLDL = very low-density lipoprotein; LDL = low-density lipoprotein; HDL = high-density lipoprotein. *N* = 101,359.

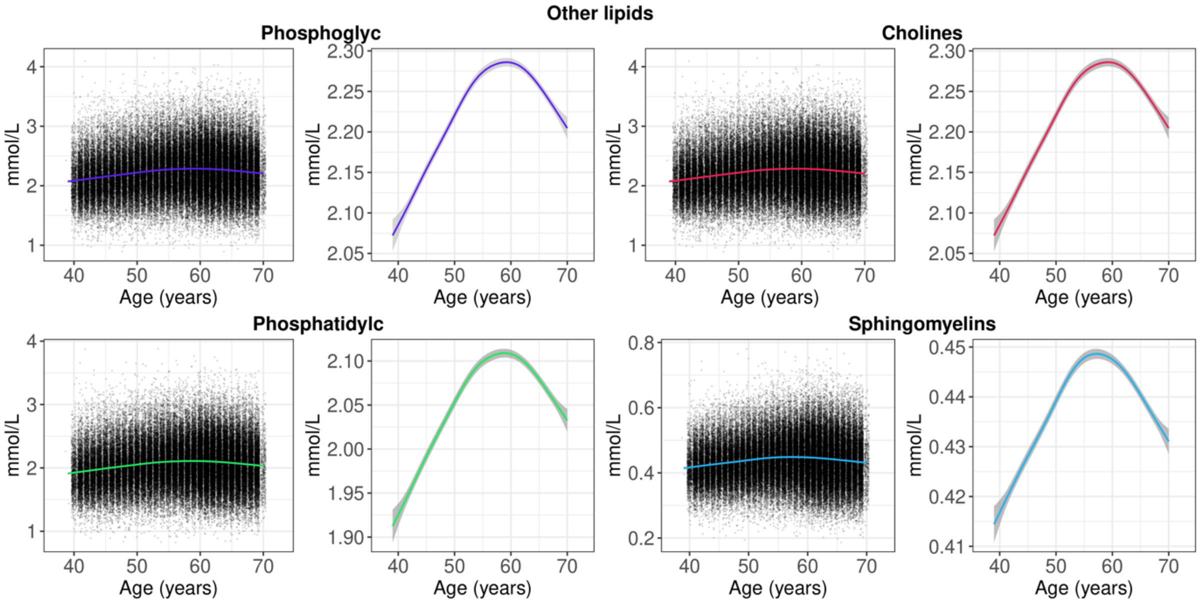

Figure S10. Distribution of other lipid levels by chronological age, showing scatter plots for all observations and smooth curves (note the difference in y-axis scale). The smooth curves were estimated using generalised additive models, with shaded areas corresponding to 95% confidence intervals. $N = 101{,}359$.

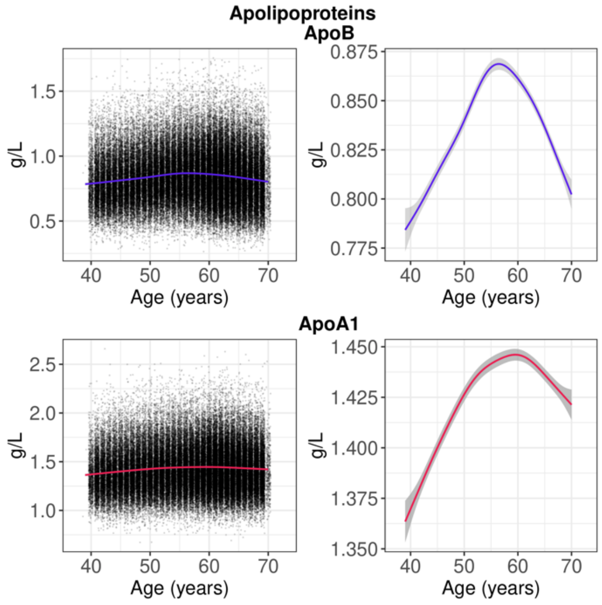

Figure S11. Distribution of apolipoprotein levels by chronological age, showing scatter plots for all observations and smooth curves (note the difference in y-axis scale). The smooth curves were estimated using generalised additive models, with shaded areas corresponding to 95% confidence intervals. ApoB = apolipoprotein B; ApoA1 = apolipoprotein A1. N = 101,359.

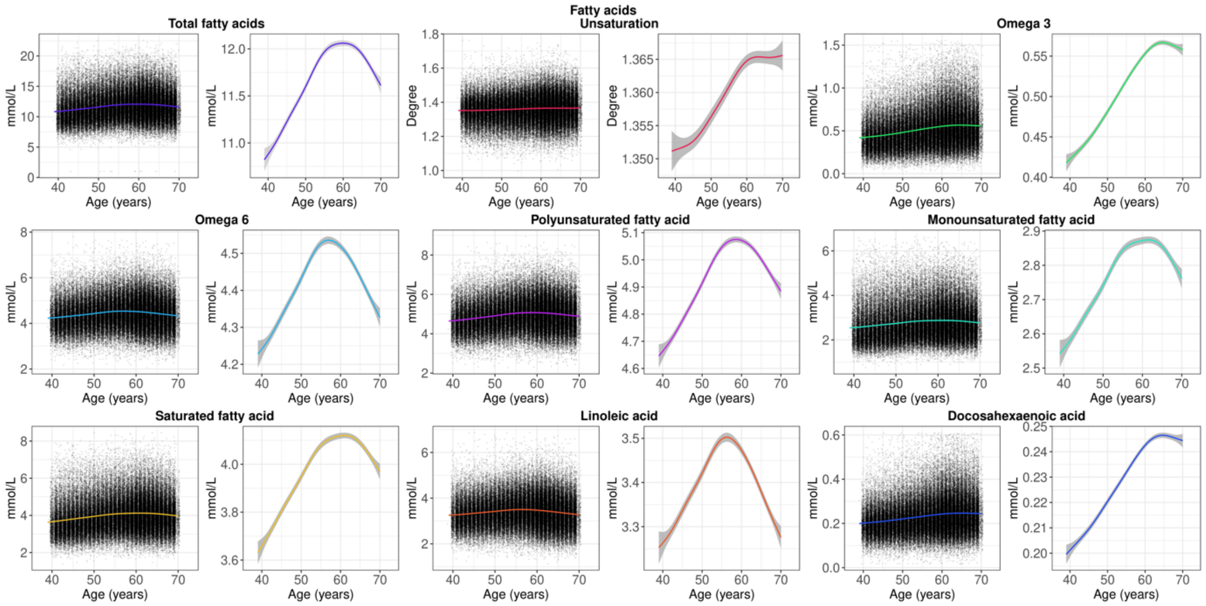

Figure S12. Distribution of fatty acid levels by chronological age, showing scatter plots for all observations and smooth curves (note the difference in y-axis scale). The smooth curves were estimated using generalised additive models, with shaded areas corresponding to 95% confidence intervals. *N* = 101,359.

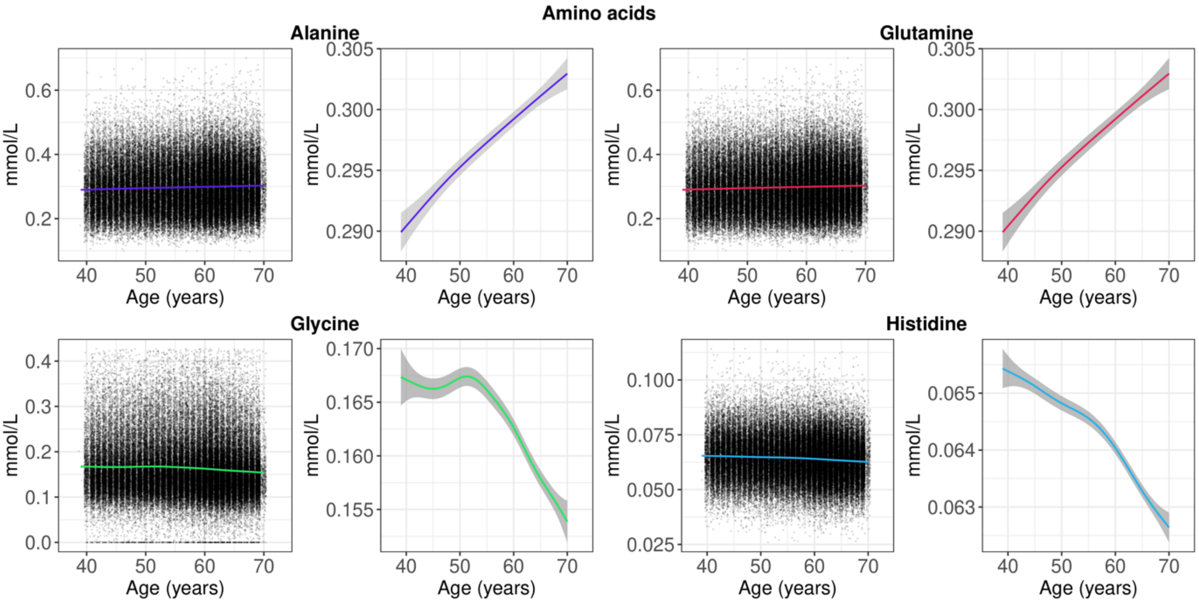

Figure S13. Distribution of amino acid levels by chronological age, showing scatter plots for all observations and smooth curves (note the difference in y-axis scale). The smooth curves were estimated using generalised additive models, with shaded areas corresponding to 95% confidence intervals. N = 101,359.

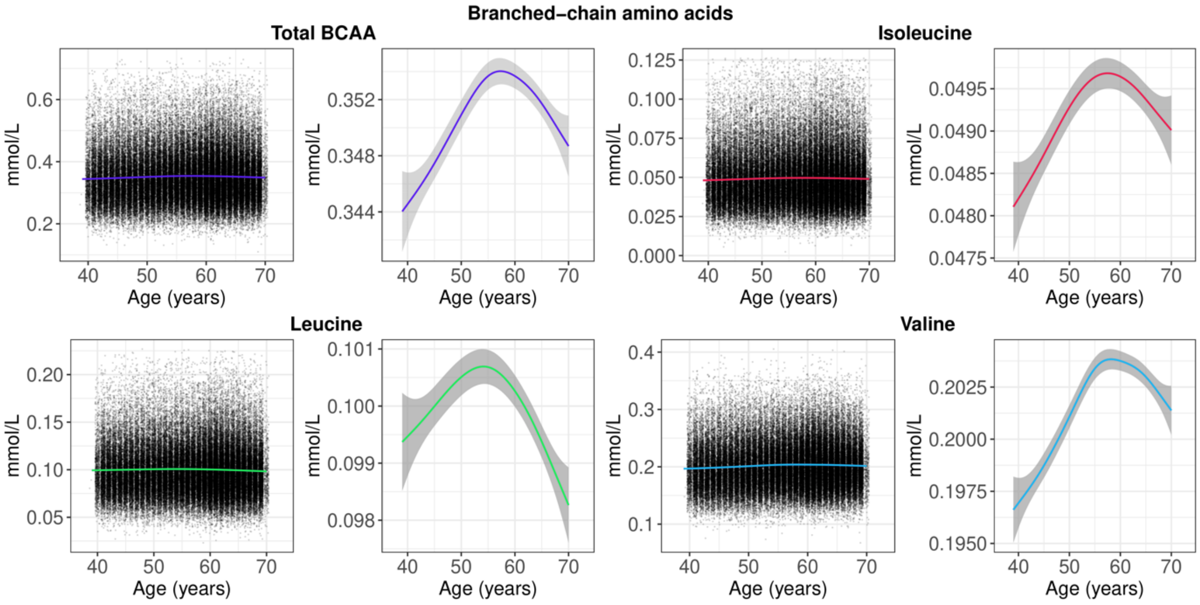

Figure S14. Distribution of branched-chain amino acid levels by chronological age, showing scatter plots for all observations and smooth curves (note the difference in y-axis scale). The smooth curves were estimated using generalised additive models, with shaded areas corresponding to 95% confidence intervals. BCAA = branched-chain amino acids. N = 101,359.

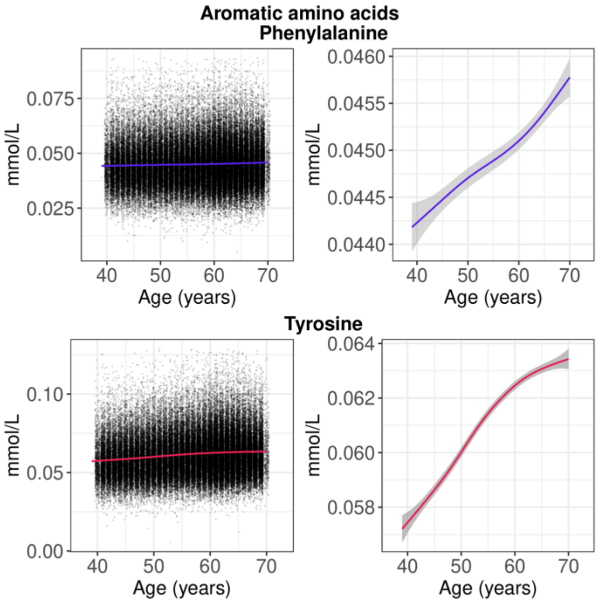

Figure S15. Distribution of aromatic amino acid levels by chronological age, showing scatter plots for all observations and smooth curves (note the difference in y-axis scale). The smooth curves were estimated using generalised additive models, with shaded areas corresponding to 95% confidence intervals. $N = 101,359$.

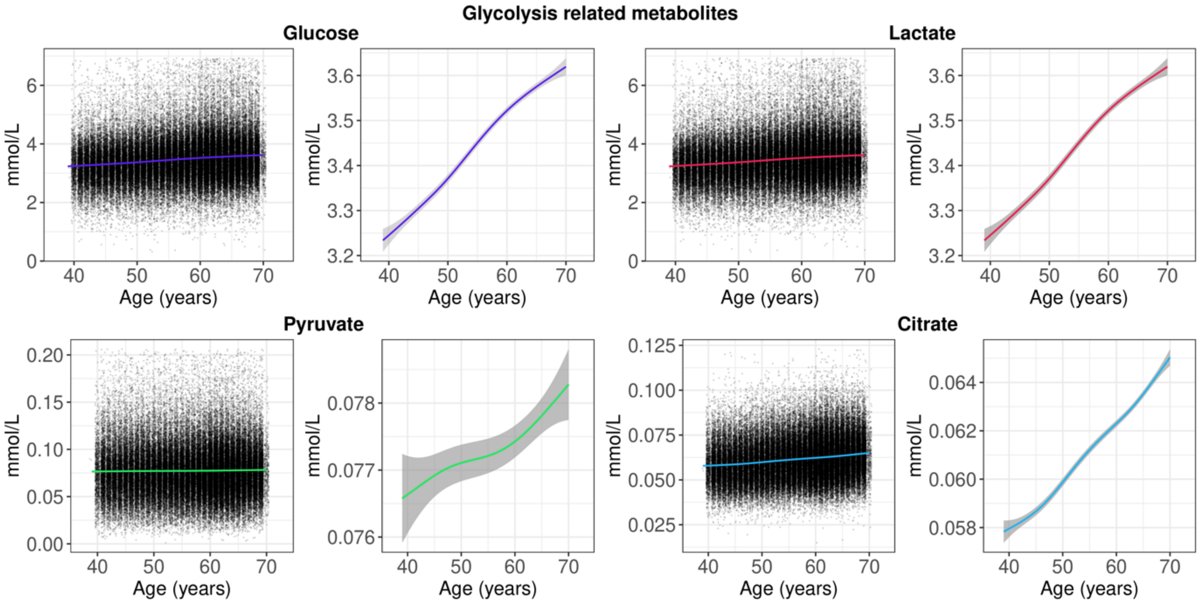

Figure S16. Distribution of glycolysis related metabolite levels by chronological age, showing scatter plots for all observations and smooth curves (note the difference in y-axis scale). The smooth curves were estimated using generalised additive models, with shaded areas corresponding to 95% confidence intervals. N = 101,359.

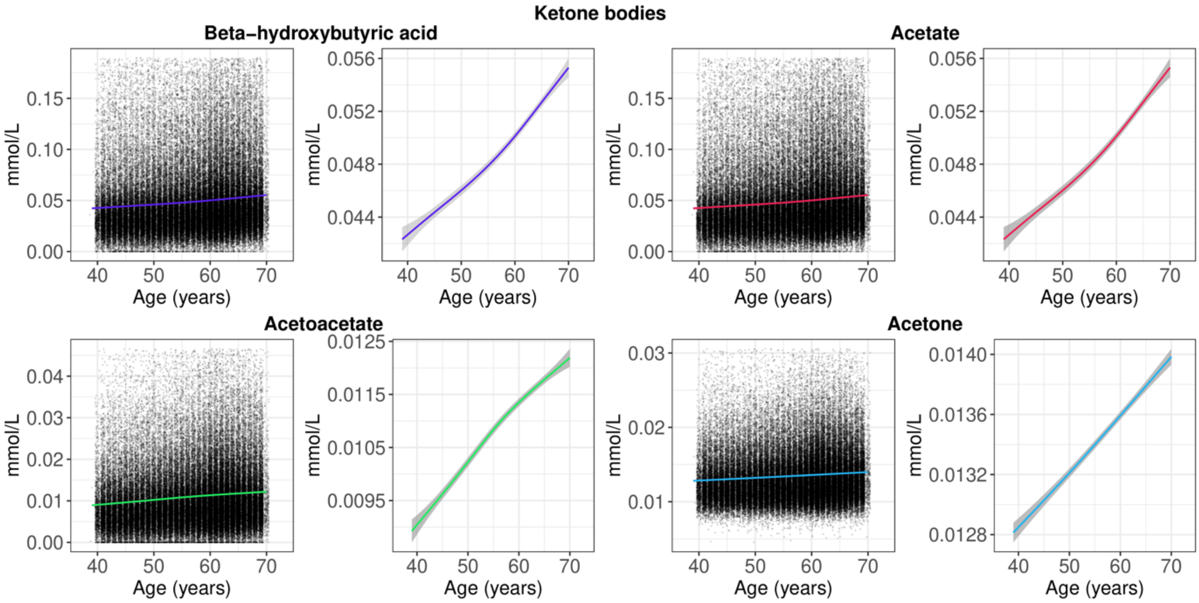

Figure S17. Distribution of ketone body levels by chronological age, showing scatter plots for all observations and smooth curves (note the difference in y-axis scale). The smooth curves were estimated using generalised additive models, with shaded areas corresponding to 95% confidence intervals. *N* = 101,359.

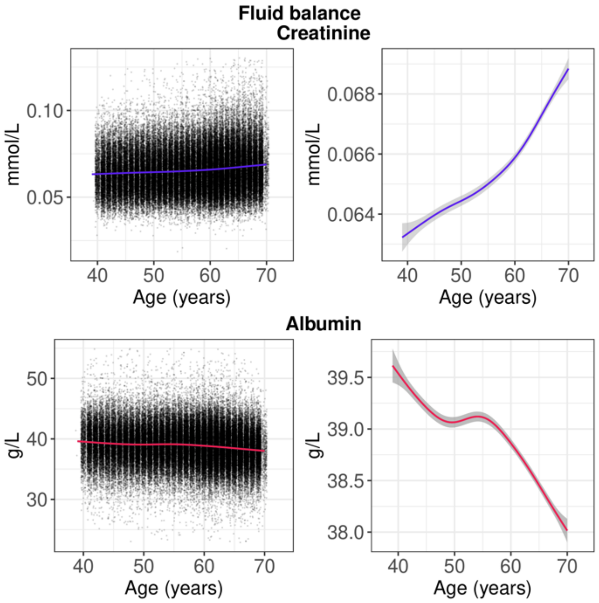

Figure S18. Distribution of metabolites involved in fluid balance by chronological age, showing scatter plots for all observations and smooth curves (note the difference in y-axis scale). The smooth curves were estimated using generalised additive models, with shaded areas corresponding to 95% confidence intervals. *N* = 101,359.

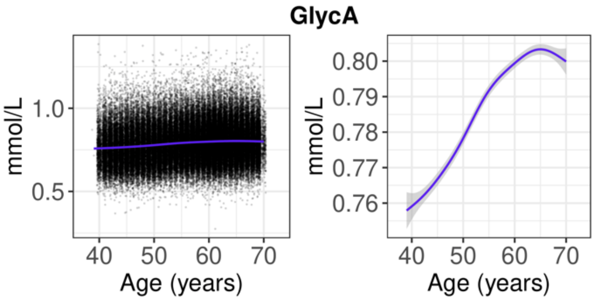

Figure S19. Distribution of glycoprotein acetyl levels by chronological age, showing scatter plots for all observations and smooth curves (note the difference in y-axis scale). The smooth curves were estimated using generalised additive models, with shaded areas corresponding to 95% confidence intervals. GlycA = glycoprotein acetyls. *N* = 101,359.

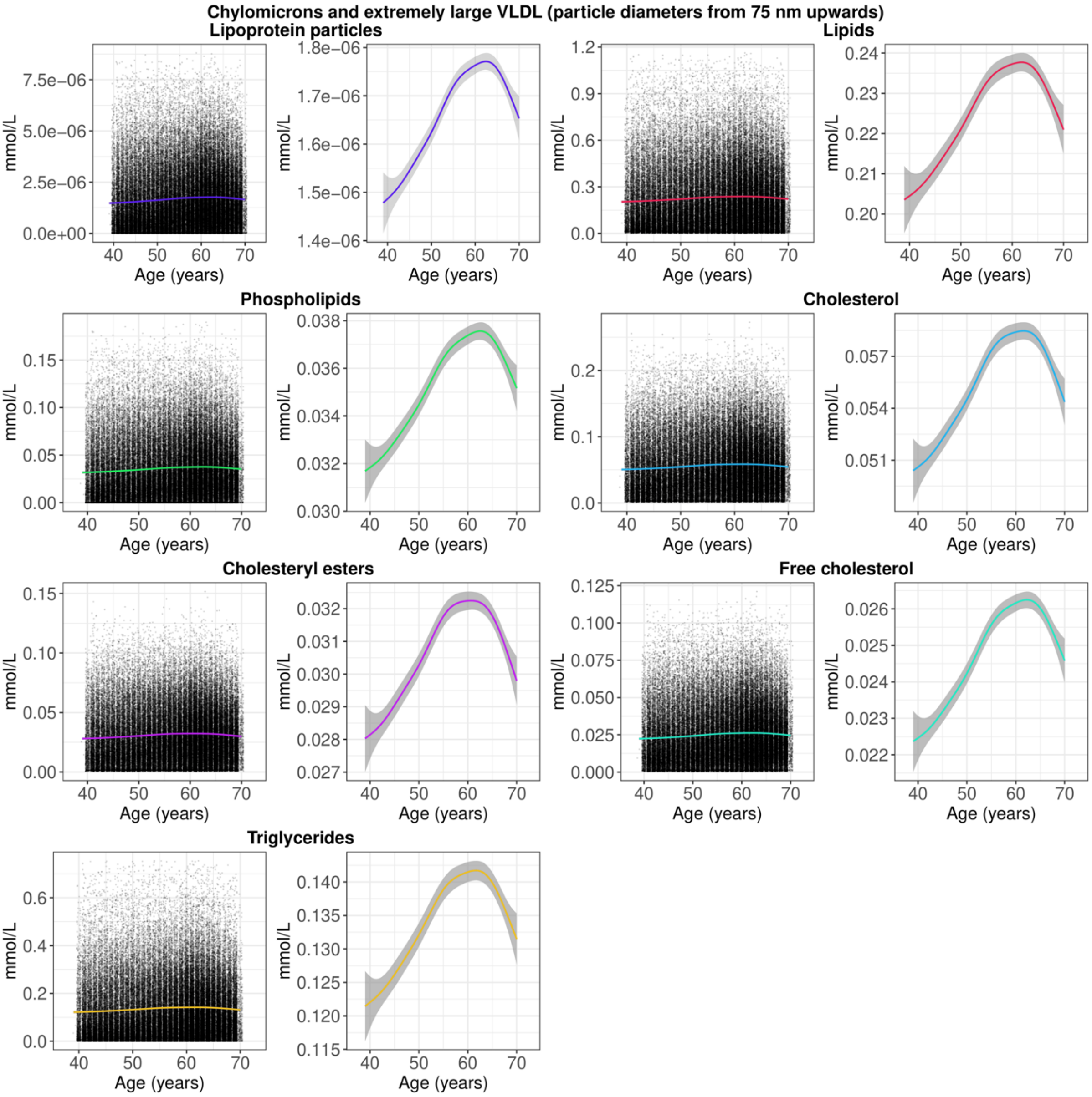

Figure S20. Distribution of chylomicrons and extremely large VLDL (particle diameters from 75 nm upwards) by chronological age, showing scatter plots for all observations and smooth curves (note the difference in y-axis scale). The smooth curves were estimated using generalised additive models, with shaded areas corresponding to 95% confidence intervals. VLDL = very low-density lipoprotein. N = 101,359.

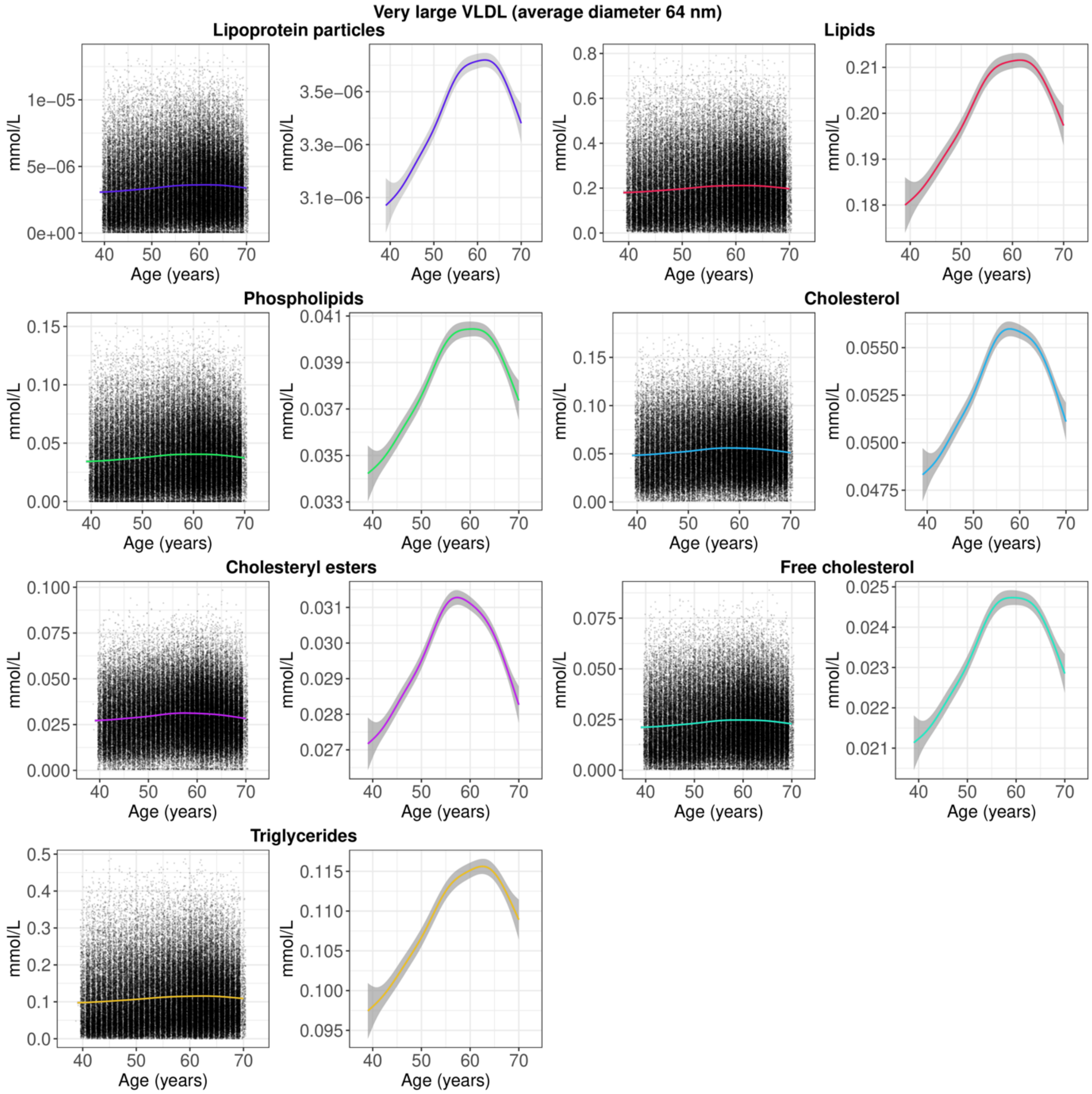

Figure S21. Distribution of very large VLDL (64 nm average diameter) by chronological age, showing scatter plots for all observations and smooth curves (note the difference in y-axis scale). The smooth curves were estimated using generalised additive models, with shaded areas corresponding to 95% confidence intervals. VLDL = very low-density lipoprotein. *N* = 101,359.

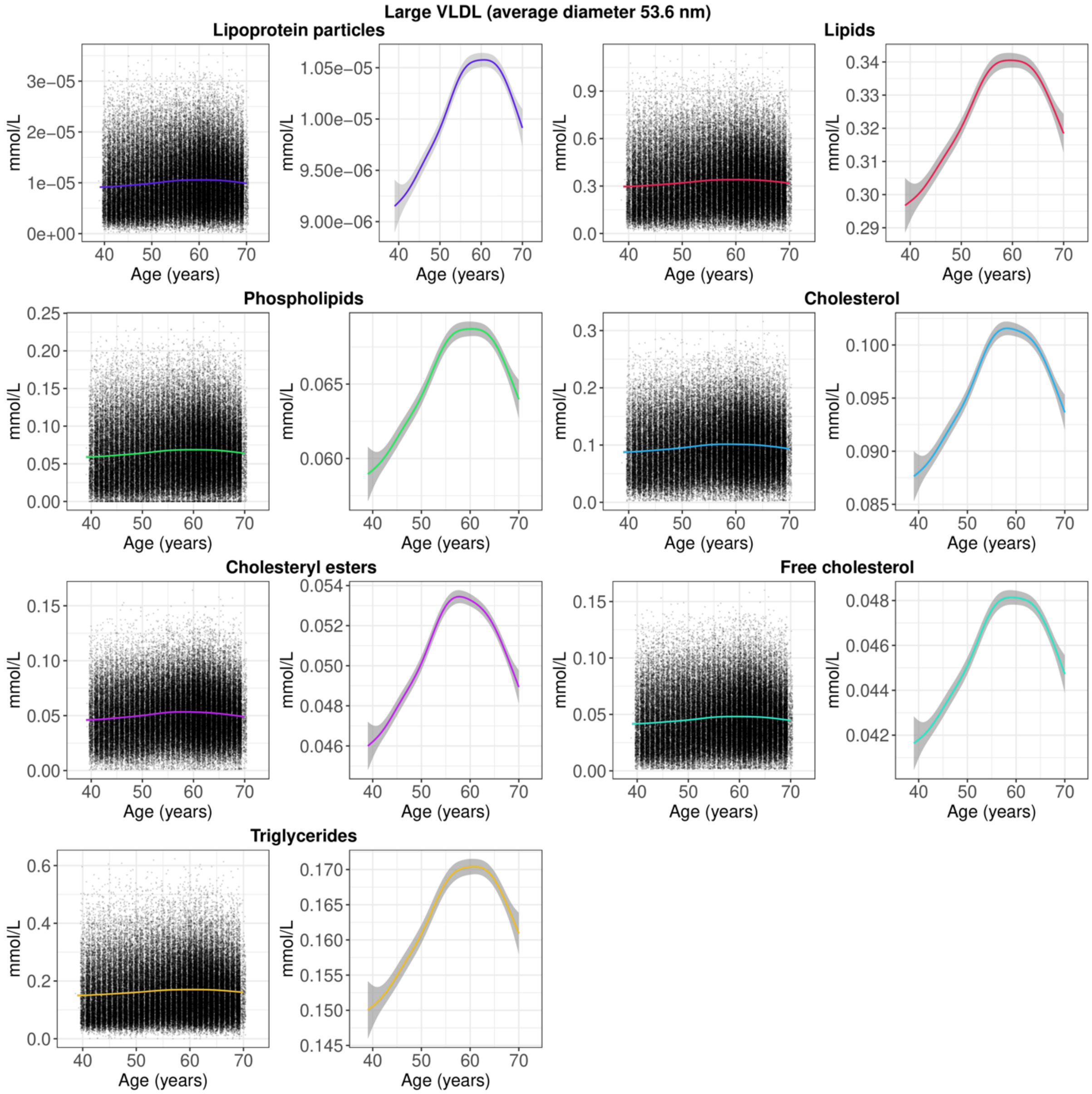

Figure S22. Distribution of large VLDL (average diameter 53.6 nm) by chronological age, showing scatter plots for all observations and smooth curves (note the difference in y-axis scale). The smooth curves were estimated using generalised additive models, with shaded areas corresponding to 95% confidence intervals. VLDL = very low-density lipoprotein. *N* = 101,359.

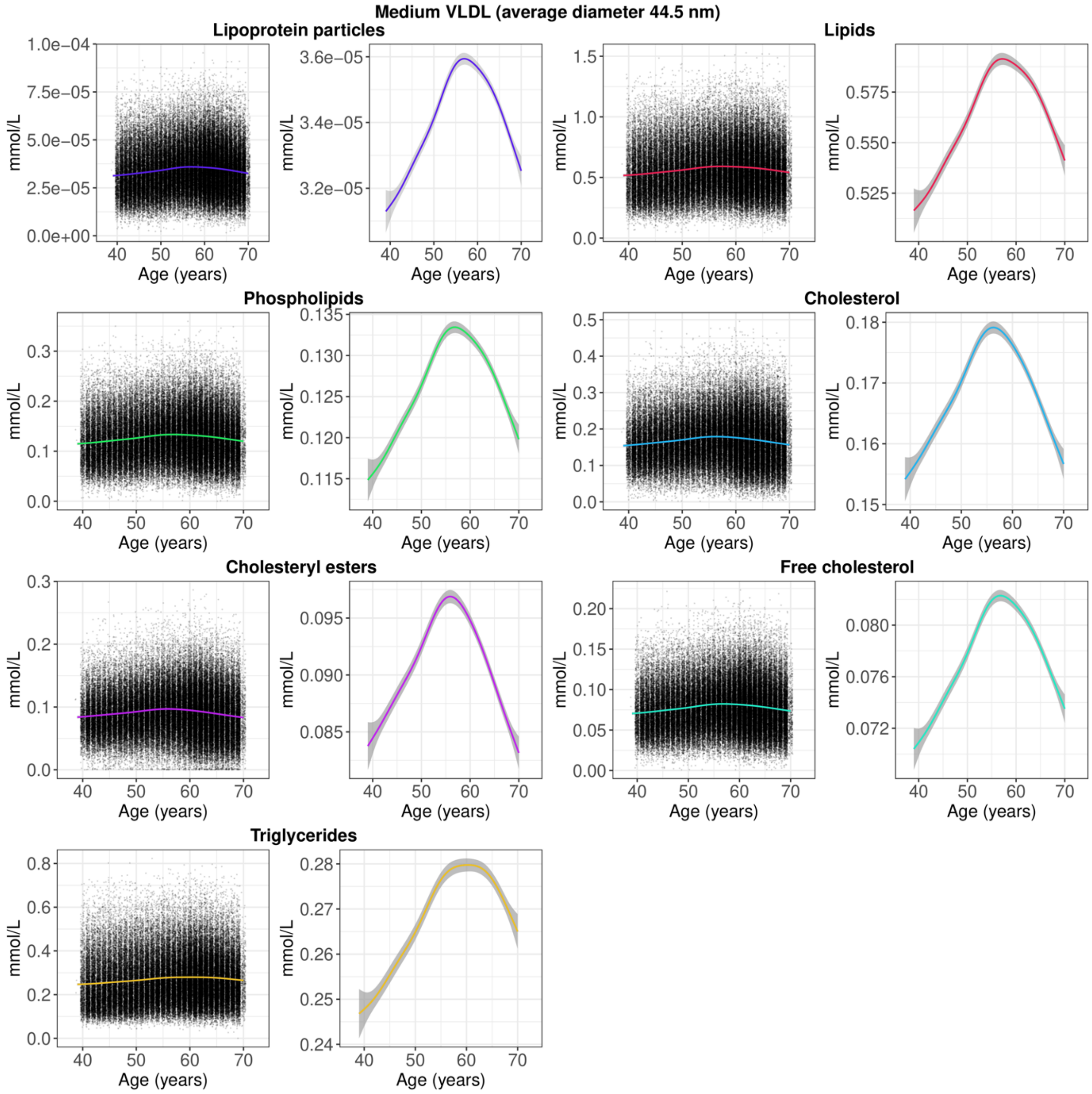

Figure S23. Distribution of medium VLDL (average diameter 44.5 nm) by chronological age, showing scatter plots for all observations and smooth curves (note the difference in y-axis scale). The smooth curves were estimated using generalised additive models, with shaded areas corresponding to 95% confidence intervals. VLDL = very low-density lipoprotein. *N* = 101,359.

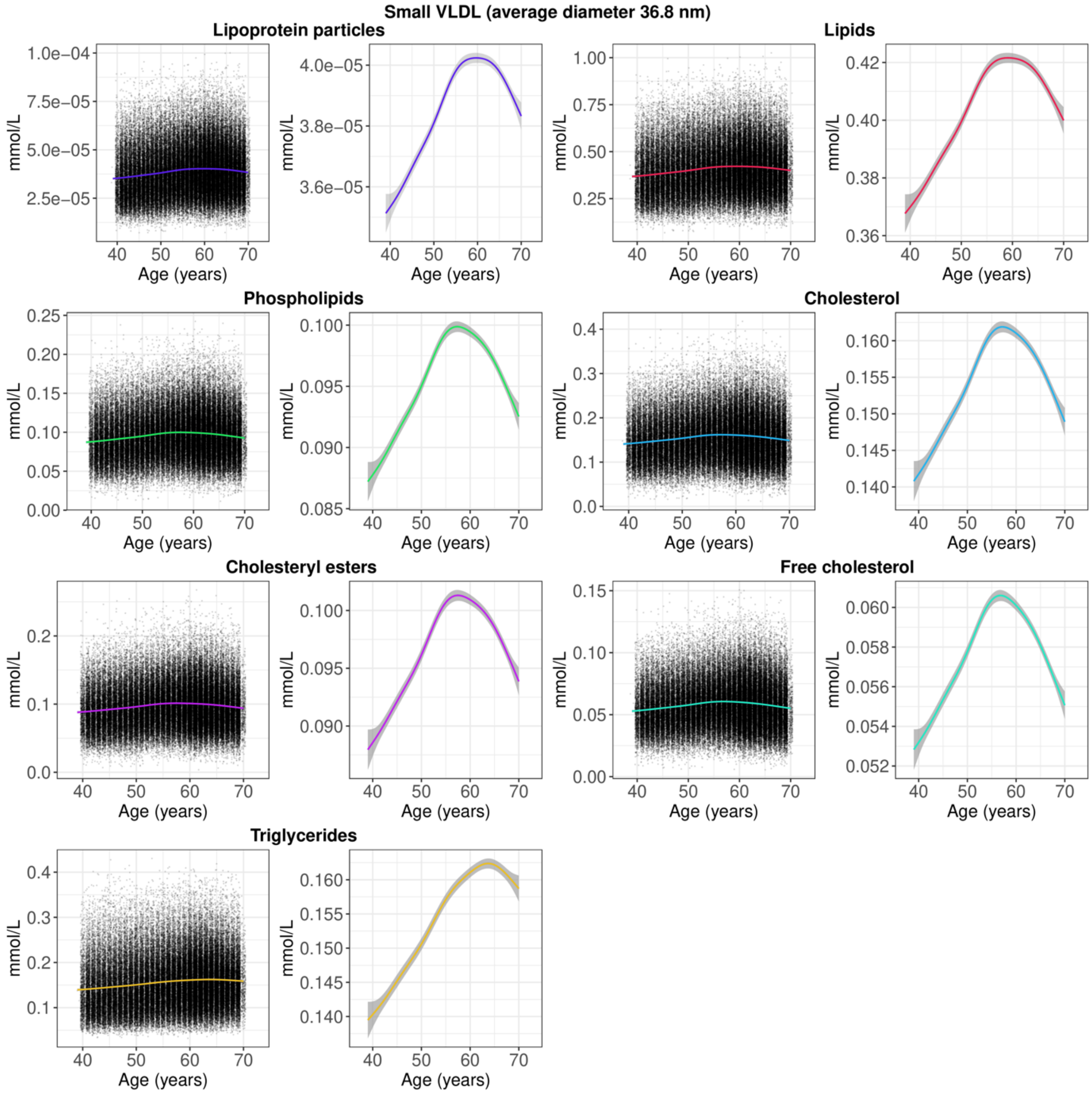

Figure S24. Distribution of small VLDL (average diameter 36.8 nm) by chronological age, showing scatter plots for all observations and smooth curves (note the difference in y-axis scale). The smooth curves were estimated using generalised additive models, with shaded areas corresponding to 95% confidence intervals. VLDL = very low-density lipoprotein. N = 101,359.

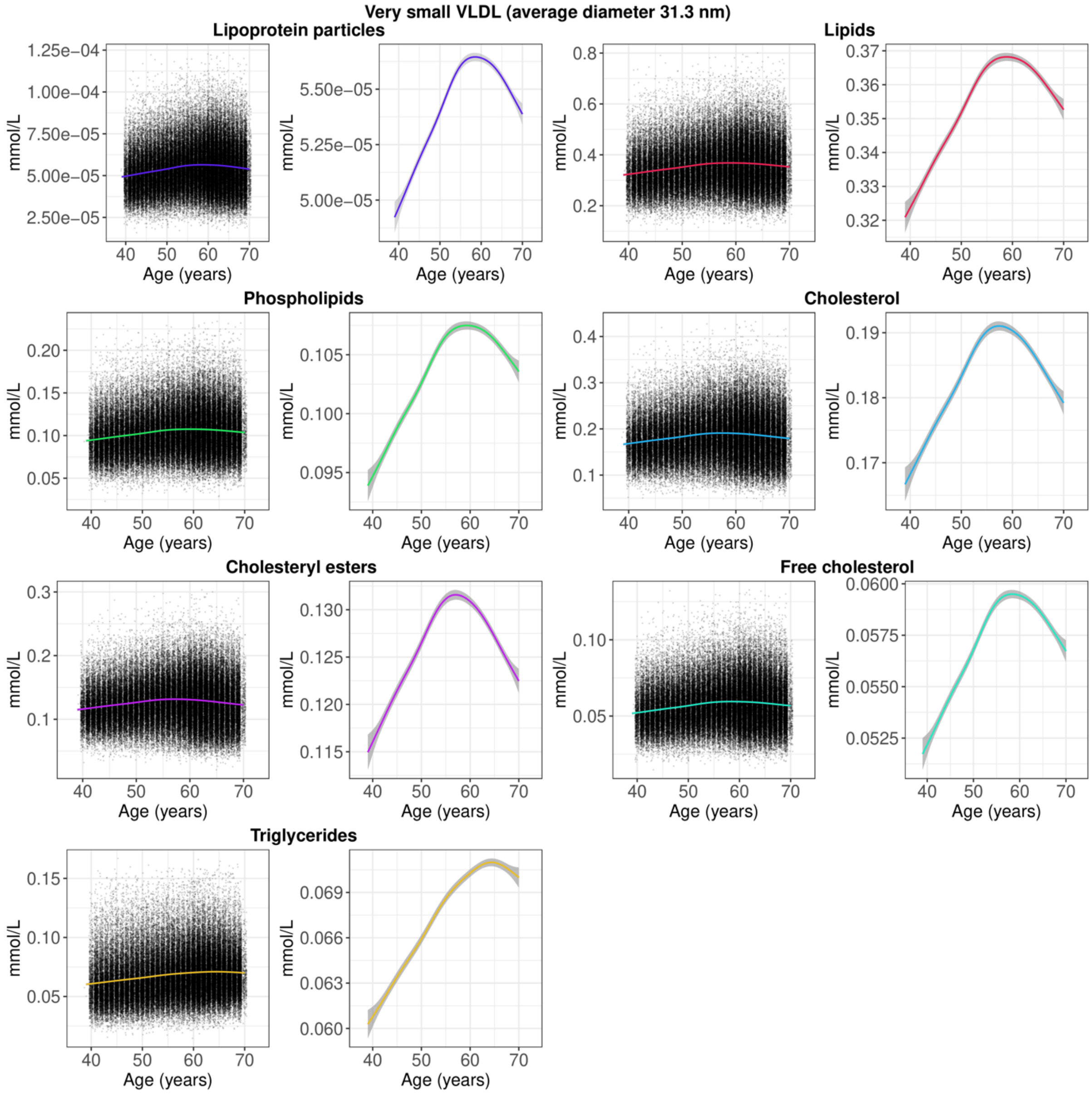

Figure S25. Distribution of very small VLDL (average diameter 31.3 nm) by chronological age, showing scatter plots for all observations and smooth curves (note the difference in y-axis scale). The smooth curves were estimated using generalised additive models, with shaded areas corresponding to 95% confidence intervals. VLDL = very low-density lipoprotein. N = 101,359.

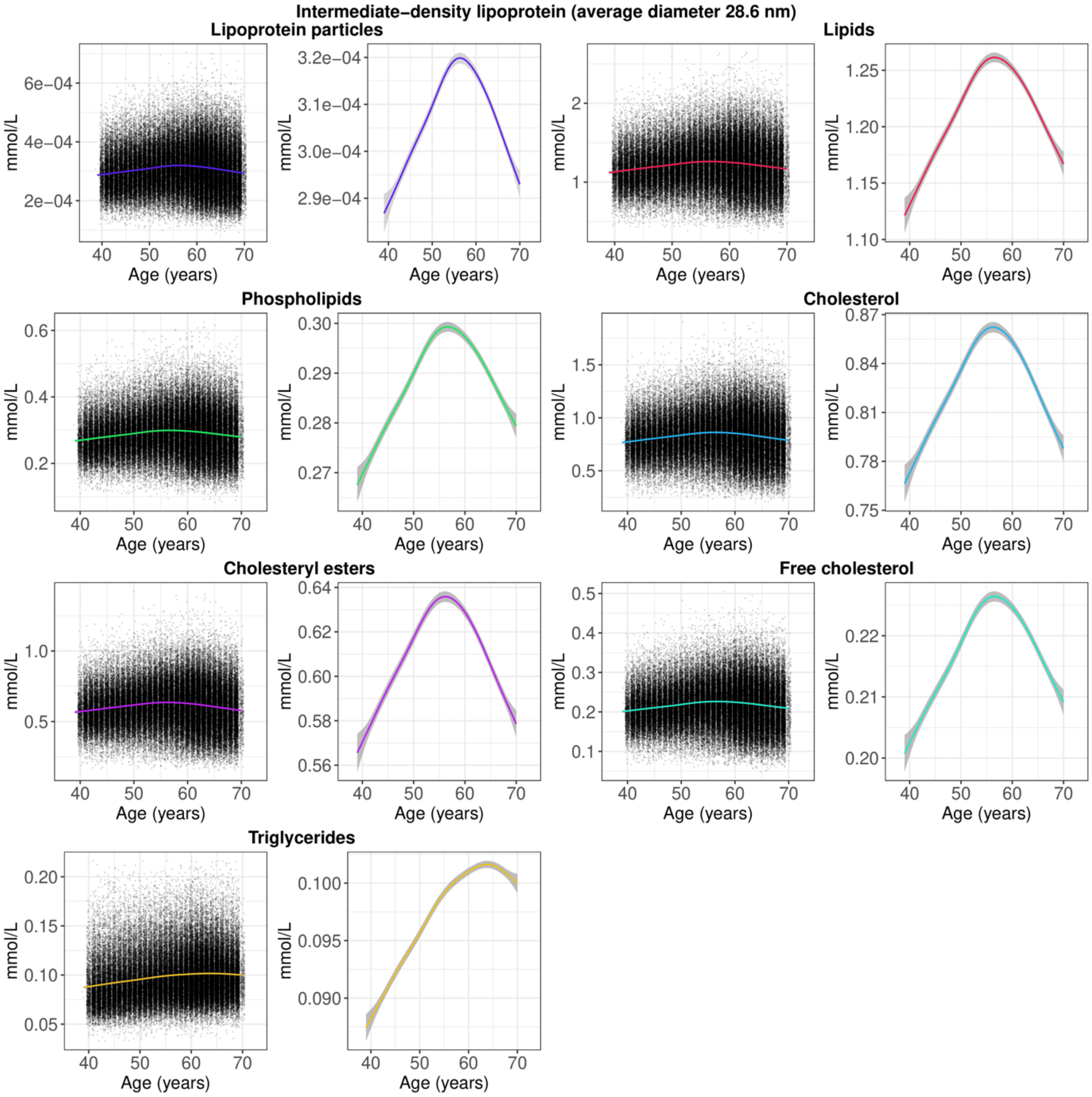

Figure S26. Distribution of intermediate-density lipoprotein (average diameter 28.6 nm) by chronological age, showing scatter plots for all observations and smooth curves (note the difference in y-axis scale). The smooth curves were estimated using generalised additive models, with shaded areas corresponding to 95% confidence intervals. $N = 101{,}359$.

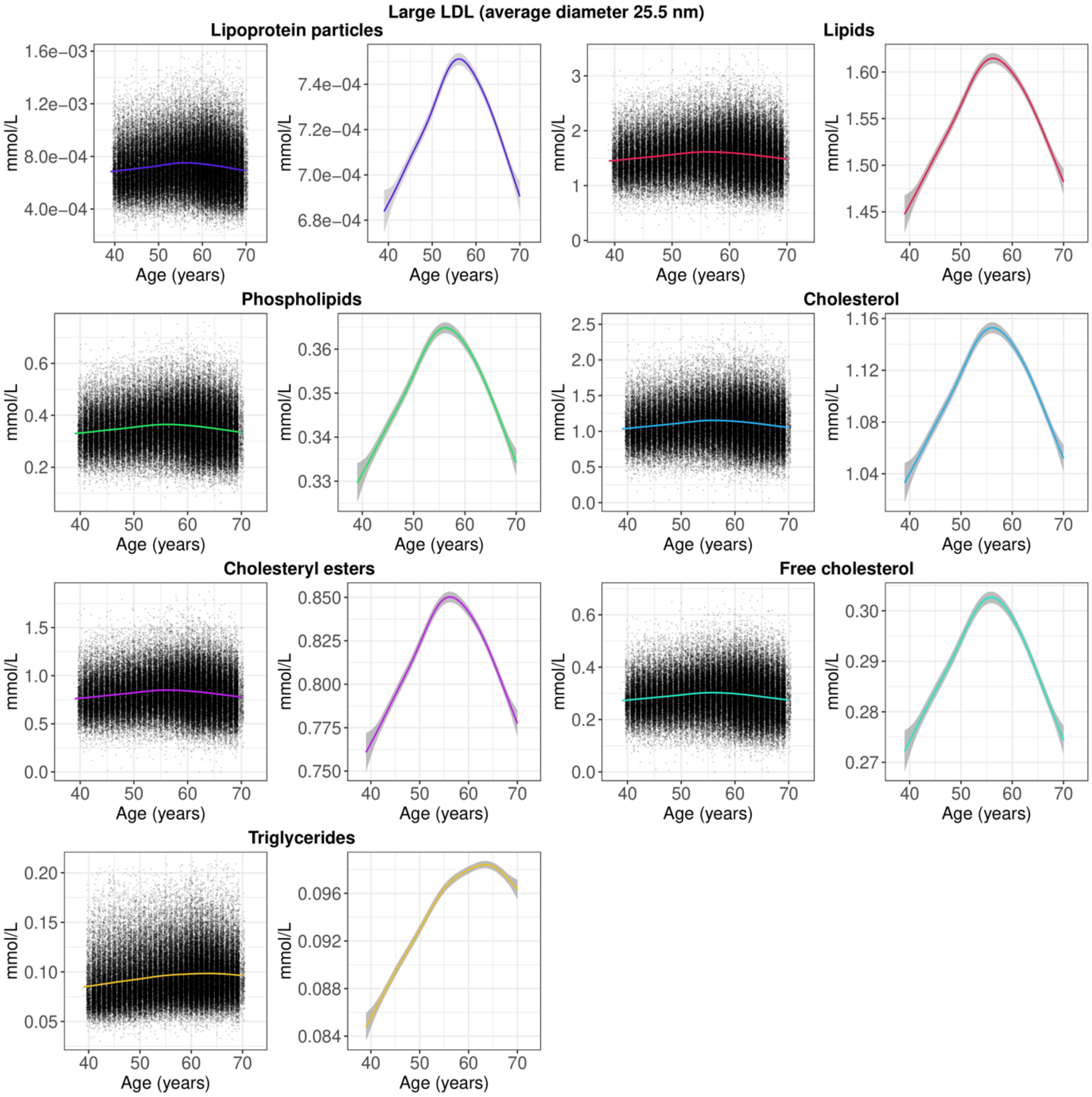

Figure S27. Distribution of large LDL (average diameter 25.5 nm) by chronological age, showing scatter plots for all observations and smooth curves (note the difference in y-axis scale). The smooth curves were estimated using generalised additive models, with shaded areas corresponding to 95% confidence intervals. LDL = low-density lipoprotein. *N* = 101,359.

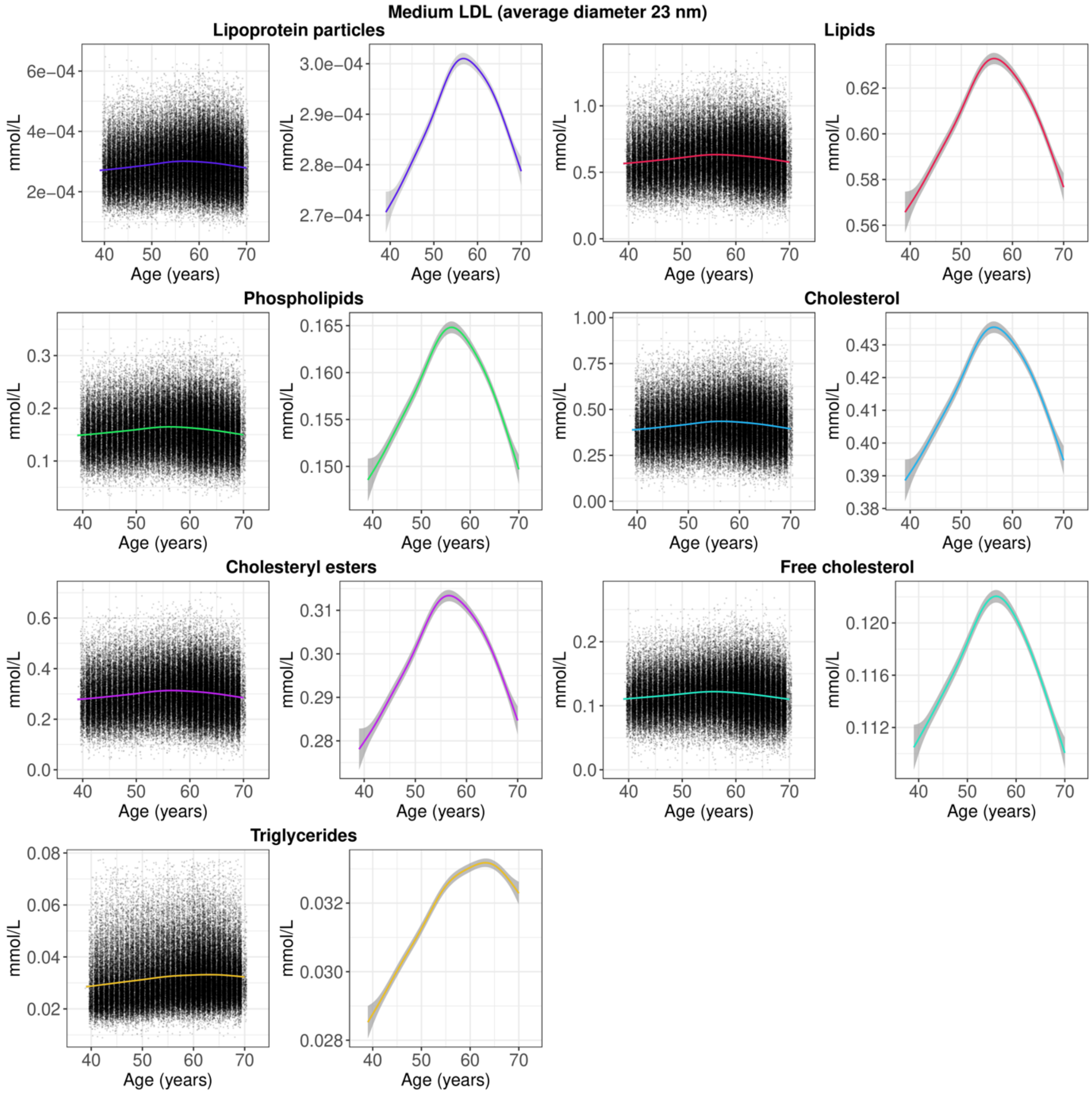

Figure S28. Distribution of medium LDL (average diameter 23 nm) by chronological age, showing scatter plots for all observations and smooth curves (note the difference in y-axis scale). The smooth curves were estimated using generalised additive models, with shaded areas corresponding to 95% confidence intervals. LDL = low-density lipoprotein. *N* = 101,359.

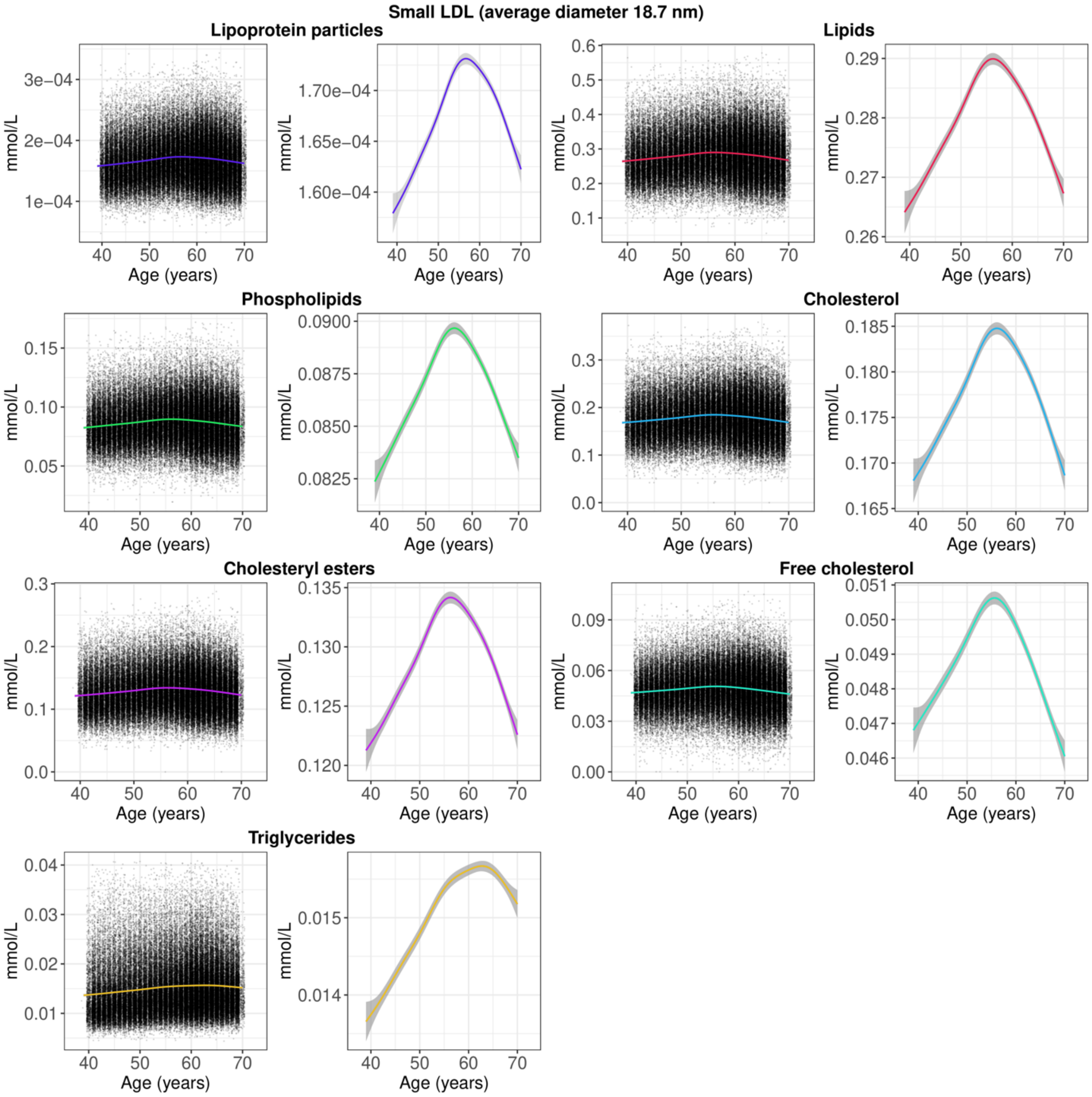

Figure S29. Distribution of small LDL (average diameter 18.7 nm) by chronological age, showing scatter plots for all observations and smooth curves (note the difference in y-axis scale). The smooth curves were estimated using generalised additive models, with shaded areas corresponding to 95% confidence intervals. LDL = low-density lipoprotein. N = 101,359.

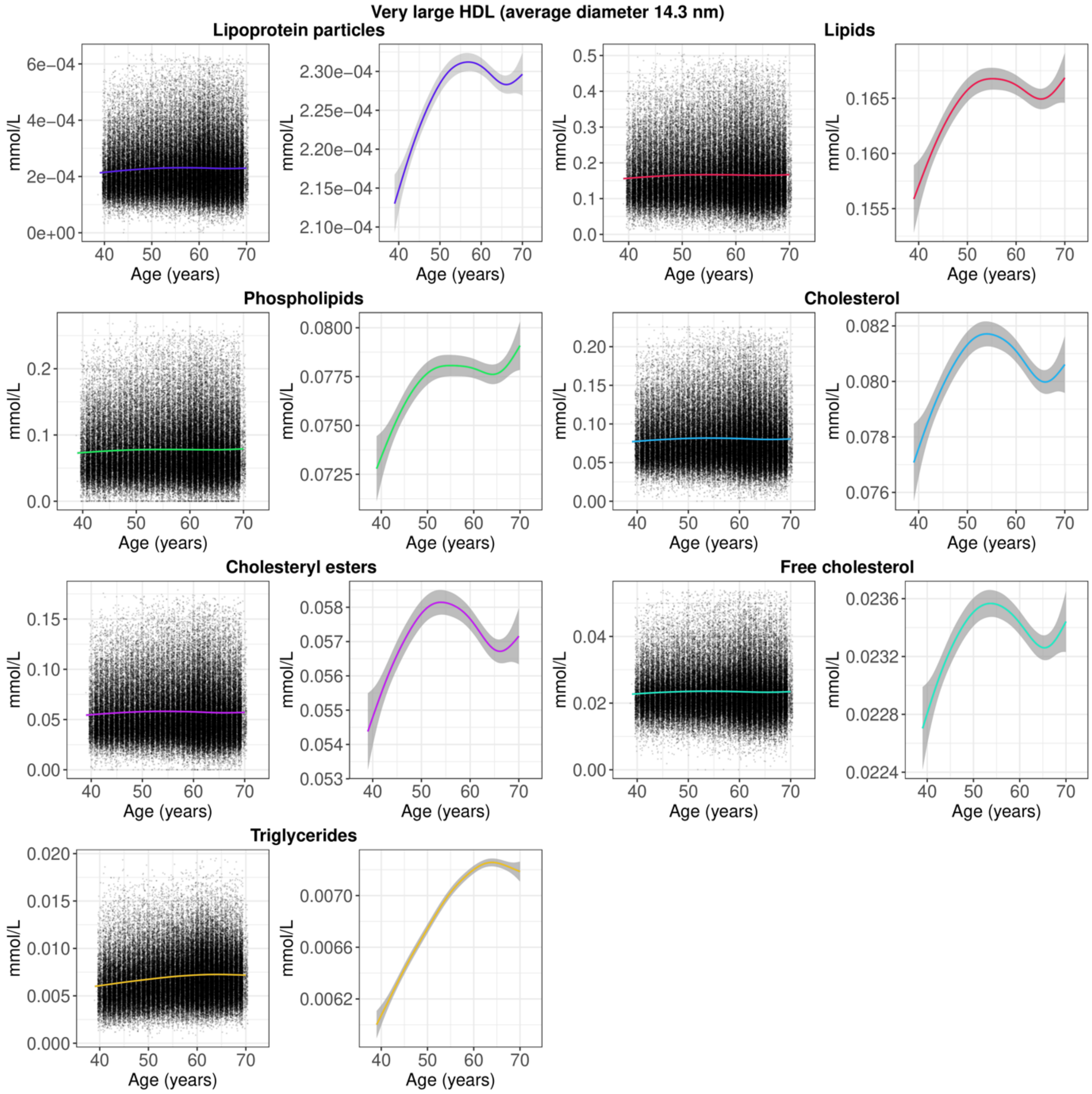

Figure S30. Distribution of very large HDL (average diameter 14.3 nm) by chronological age, showing scatter plots for all observations and smooth curves (note the difference in y-axis scale). The smooth curves were estimated using generalised additive models, with shaded areas corresponding to 95% confidence intervals. HDL = high-density lipoprotein. *N* = 101,359.

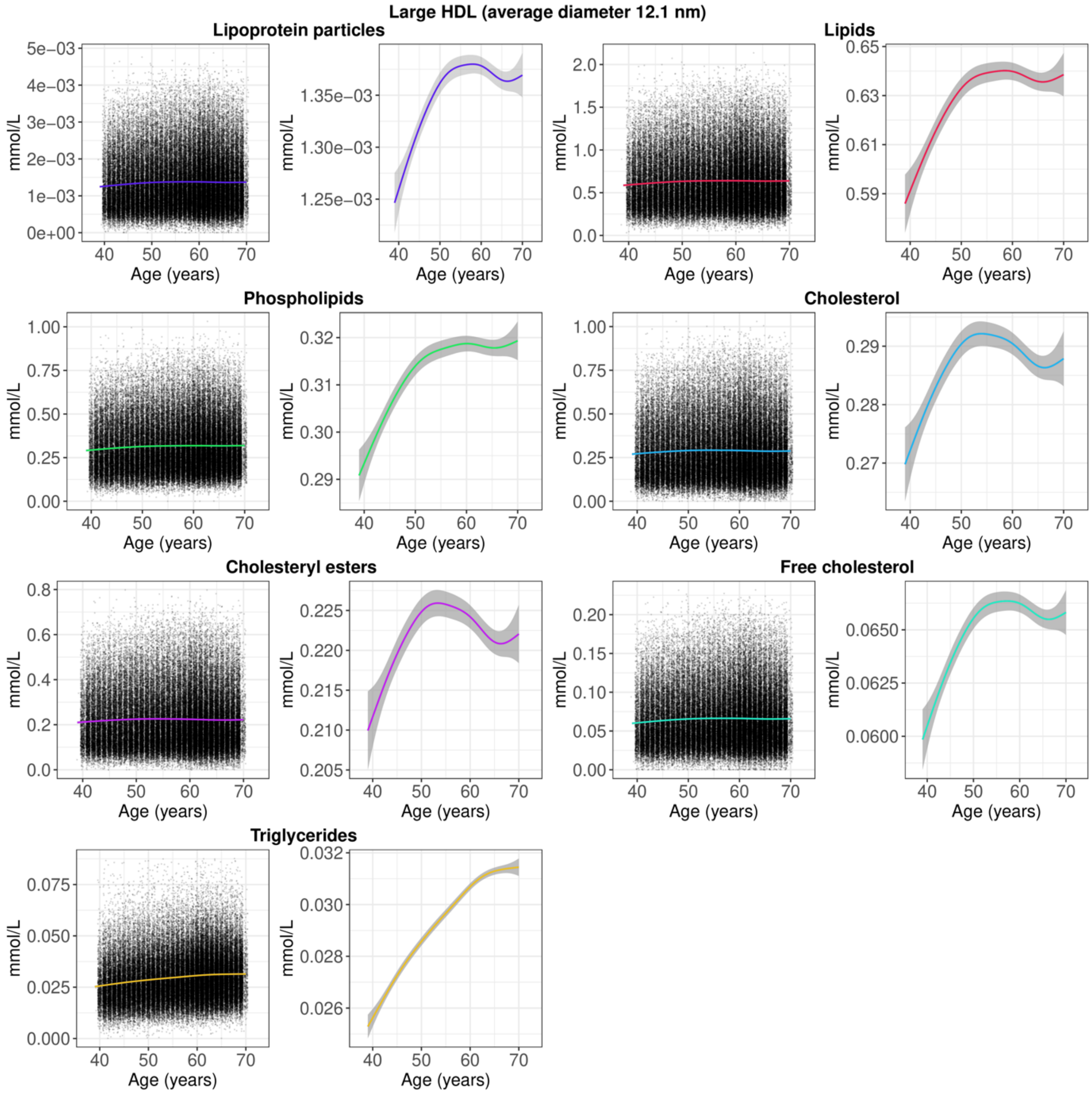

Figure S31. Distribution of large HDL (average diameter 12.1 nm) by chronological age, showing scatter plots for all observations and smooth curves (note the difference in y-axis scale). The smooth curves were estimated using generalised additive models, with shaded areas corresponding to 95% confidence intervals. HDL = high-density lipoprotein. *N* = 101,359.

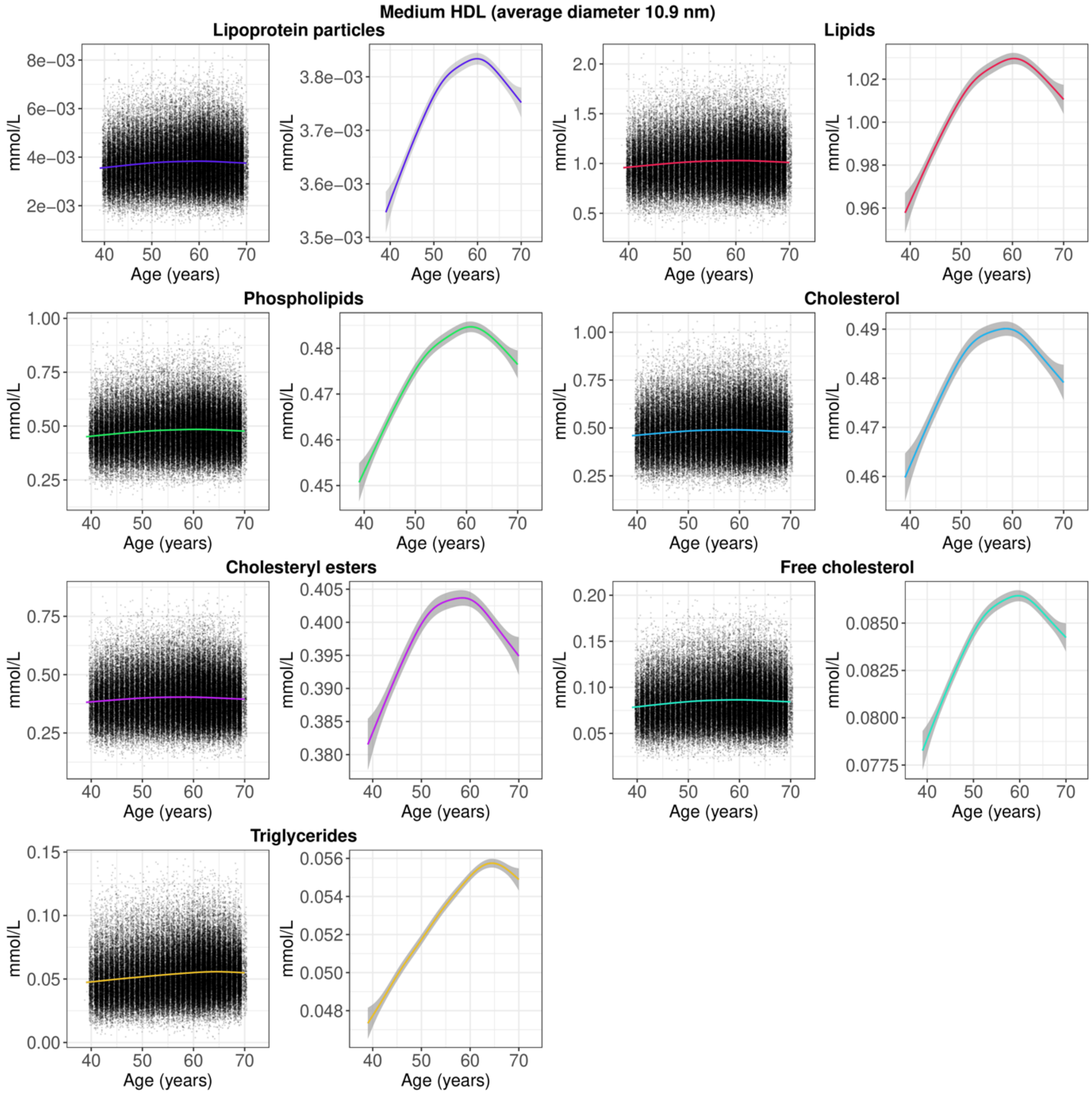

Figure S32. Distribution of medium HDL (average diameter 10.9 nm) by chronological age, showing scatter plots for all observations and smooth curves (note the difference in y-axis scale). The smooth curves were estimated using generalised additive models, with shaded areas corresponding to 95% confidence intervals. HDL = high-density lipoprotein. *N* = 101,359.

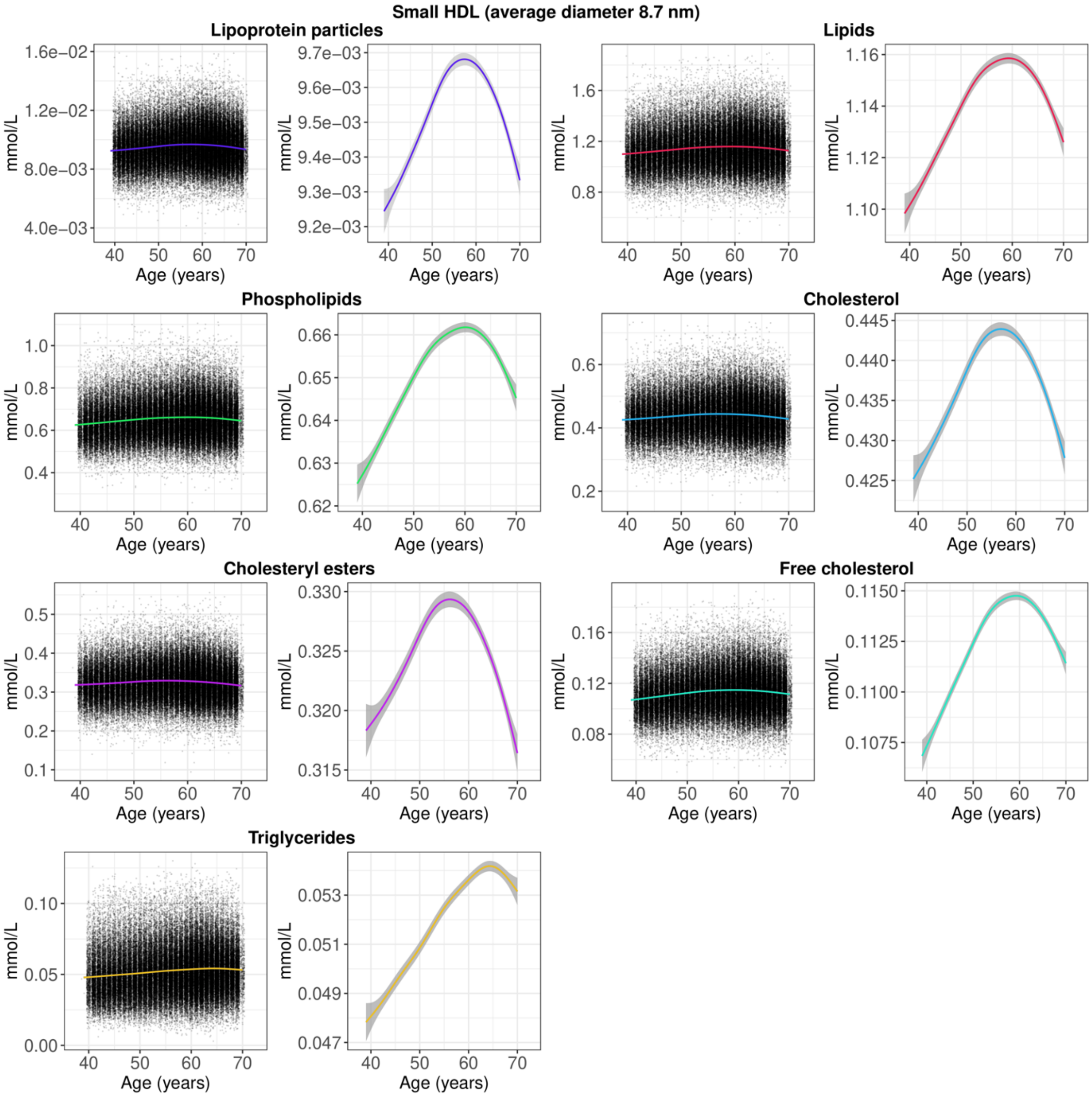

Figure S33. Distribution of small HDL (average diameter 8.7 nm) by chronological age, showing scatter plots for all observations and smooth curves (note the difference in y-axis scale). The smooth curves were estimated using generalised additive models, with shaded areas corresponding to 95% confidence intervals. HDL = high-density lipoprotein. *N* = 101,359.

5. Metabolite-wide associations

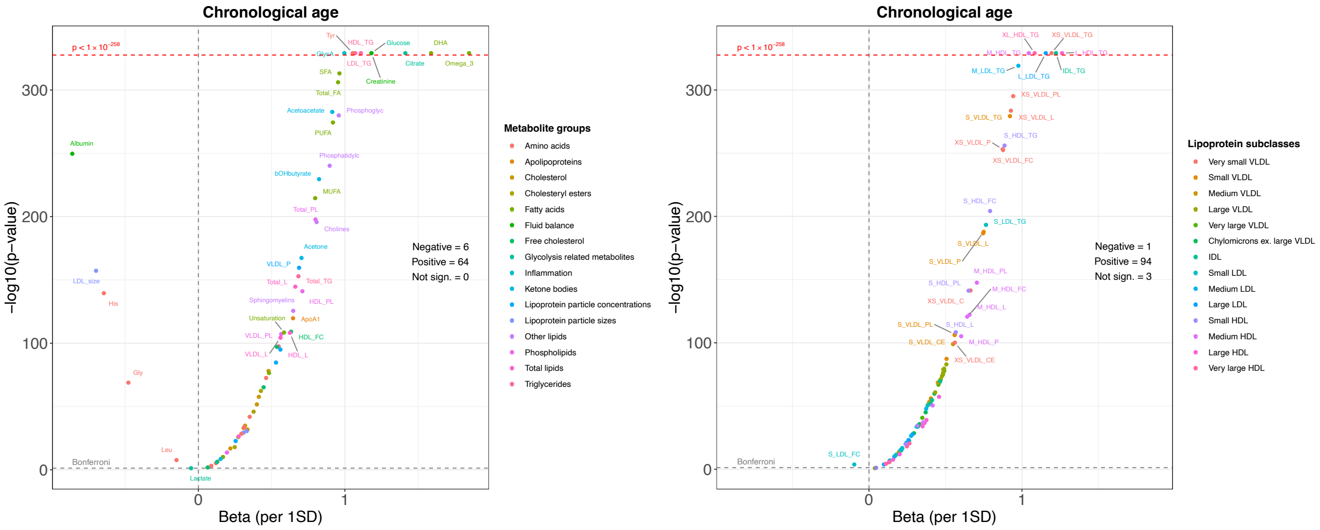

Figure S34. Volcano plots showing associations between chronological age and metabolite levels. Metabolites were standardised to have a mean equal to zero and a standard deviation of one. Associations were estimated using ordinary least squares regression. Models were adjusted for sex. *P*-values were -$\log_{10}$ transformed. *P*-values $< 1 \times 10^{-258}$ shown at the top of the plot could not be precisely estimated. SD = standard deviation; VLDL = very low-density lipoprotein; IDL = intermediate-density lipoprotein; LDL = low-density lipoprotein; HDL = high-density lipoprotein. *N* = 101,359.

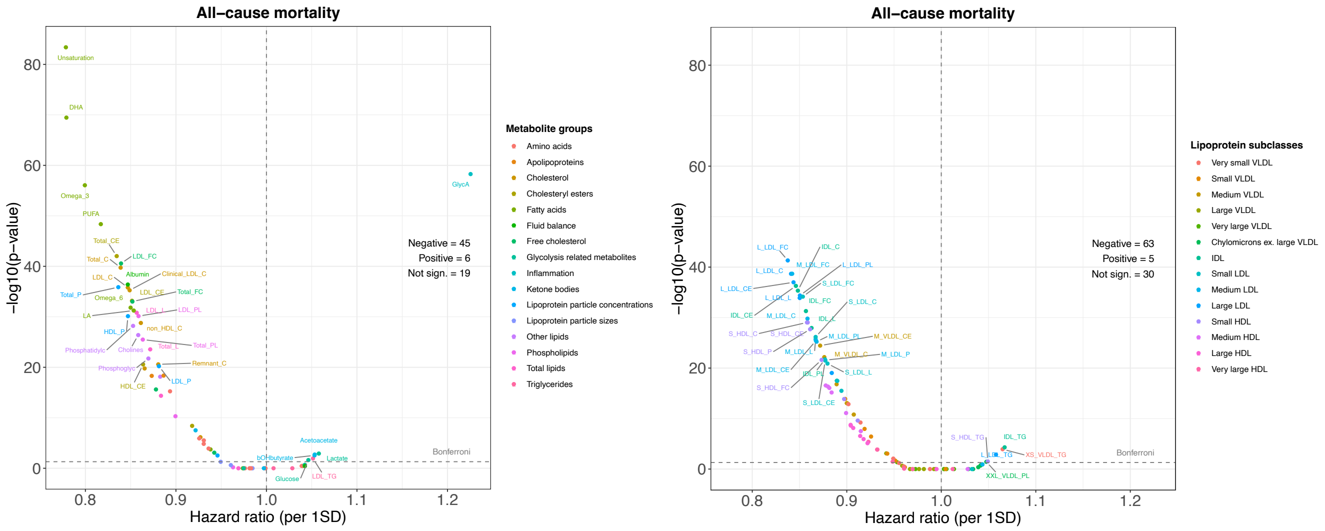

Figure S35. Volcano plots showing associations between metabolite levels and all-cause mortality. Metabolites were standardised to have a mean equal to zero and a standard deviation of one. Hazard ratios were estimated using Cox proportional hazards models adjusted for sex, with age (in years) as the underlying time axis. *P*-values were -$\log_{10}$ transformed. SD = standard deviation; VLDL = very low-density lipoprotein; IDL = intermediate-density lipoprotein; LDL = low-density lipoprotein; HDL = high-density lipoprotein. $N = 101{,}274$.

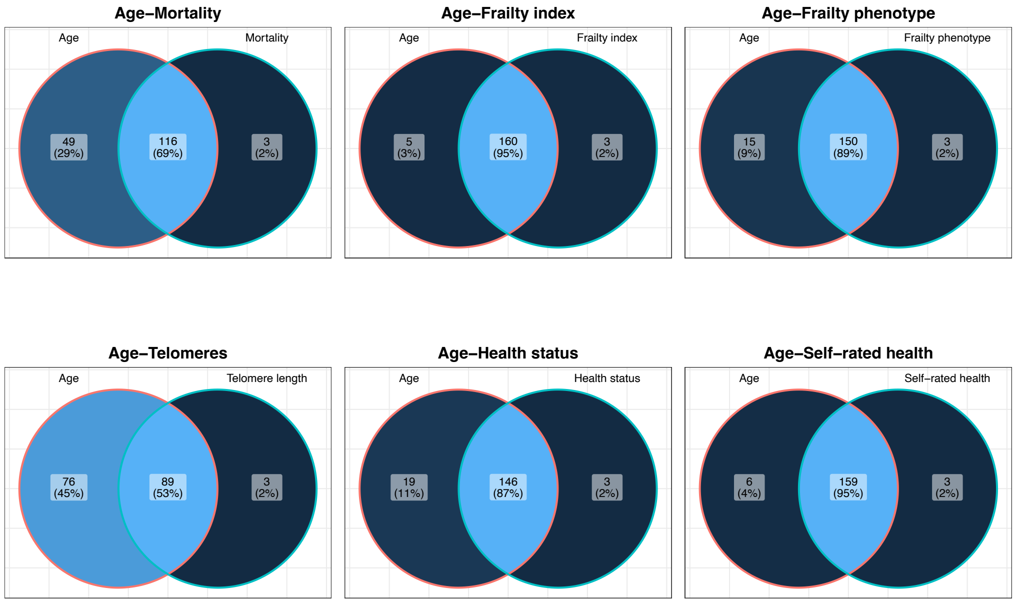

Figure S36. Venn diagrams showing the overlap between metabolites that were statistically significantly ($P < 0.05/168$) associated with chronological age and all-cause mortality or health indicators. N= 101,359 (age); N = 101,274 (mortality); N = 101,029 (frailty index); N = 95,061 (frailty phenotype); N = 97,964 (telomeres); N = 98,820 (health status); N = 100,789 (self-rated health).

6. Predictive model performance

Table S3. Predictive performance for all models with tuned hyperparameters

	MAE			RMSE			*r*			R^2		
Model	Train	Test	Diff	Train	Test	Diff	Train	Test	Diff	Train	Test	Diff
SVM radial	5.05	5.31	-0.268	6.35	6.60	-0.247	0.62	0.59	0.037	0.39	0.35	0.045
SVM polynomial	5.22	5.35	-0.135	6.52	6.63	-0.117	0.60	0.58	0.018	0.36	0.34	0.022
Cubist rules	5.22	5.42	-0.200	6.44	6.68	-0.241	0.61	0.57	0.042	0.38	0.33	0.050
Cubist rules 225k	5.22	5.35	-0.135	6.44	6.60	-0.162	0.61	0.58	0.028	0.37	0.34	0.033
RuleFit ensemble	5.39	5.52	-0.128	6.61	6.78	-0.162	0.58	0.55	0.029	0.34	0.30	0.033
XGBoost	4.08	5.59	-1.513	5.02	6.84	-1.817	0.81	0.54	0.273	0.66	0.29	0.371
BART	5.50	5.62	-0.122	6.70	6.86	-0.154	0.57	0.54	0.030	0.32	0.29	0.033
SVM linear	5.64	5.65	-0.010	6.93	6.94	-0.008	0.53	0.52	0.001	0.28	0.28	0.001
Elastic net	5.69	5.70	-0.005	6.91	6.92	-0.006	0.52	0.52	0.001	0.27	0.27	0.001
LASSO	5.69	5.70	-0.006	6.92	6.92	-0.006	0.52	0.52	0.001	0.27	0.27	0.001
Ridge	5.90	5.91	-0.003	7.11	7.11	-0.003	0.48	0.48	0.001	0.24	0.23	0.001
Random forest	2.42	5.92	-3.503	2.94	7.12	-4.174	0.98	0.49	0.487	0.95	0.24	0.713
Bagging	4.15	5.94	-1.782	5.00	7.15	-2.141	0.86	0.48	0.377	0.73	0.23	0.504
PLSR	6.19	6.19	0.002	7.41	7.41	0.001	0.41	0.41	0.000	0.17	0.17	0.000
Regression tree	5.99	6.26	-0.269	7.25	7.58	-0.335	0.45	0.37	0.083	0.20	0.13	0.068
KNN	0.47	6.30	-5.827	0.57	7.58	-7.007	0.98	0.36	0.616	0.96	0.13	0.830
MARS	6.34	6.34	0.003	7.57	7.57	0.003	0.36	0.36	-0.001	0.13	0.13	-0.001
MARS ensemble	6.36	6.36	-0.003	7.58	7.58	-0.003	0.36	0.36	0.001	0.13	0.13	0.001

Note: MAE = mean absolute error; RMSE = root mean squared error; *r* = correlation coefficient; R^2 = coefficient of determination. Models shown in ascending order by the lowest nested cross-validation MAE across the 10% test sets. See Box 1 for model abbreviations. *N* = 101,359, except for Cubist rules 225k (*N* = 225,212).

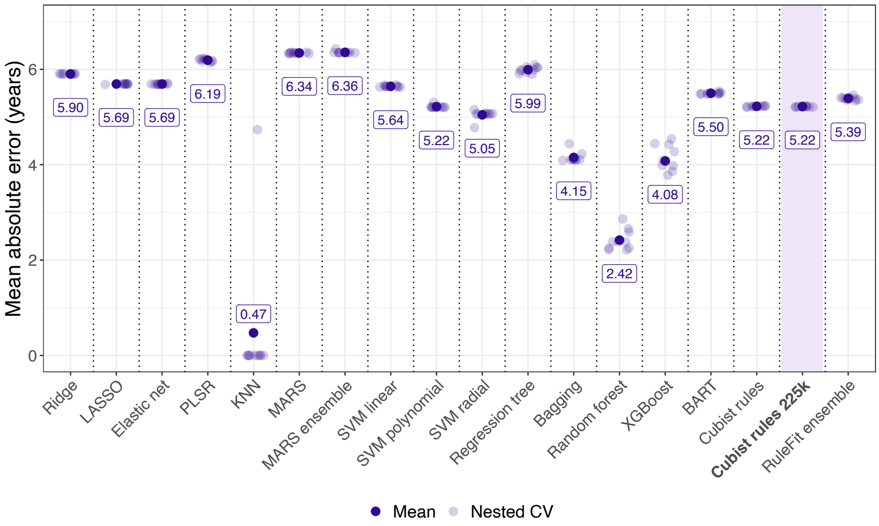

Figure S37. Nested cross-validation mean absolute error (MAE) for all models with tuned hyperparameter values in the 90% training sets. See Box 1 for model abbreviations. N = 101,359, except for Cubist rules 225k (N = 225,212).

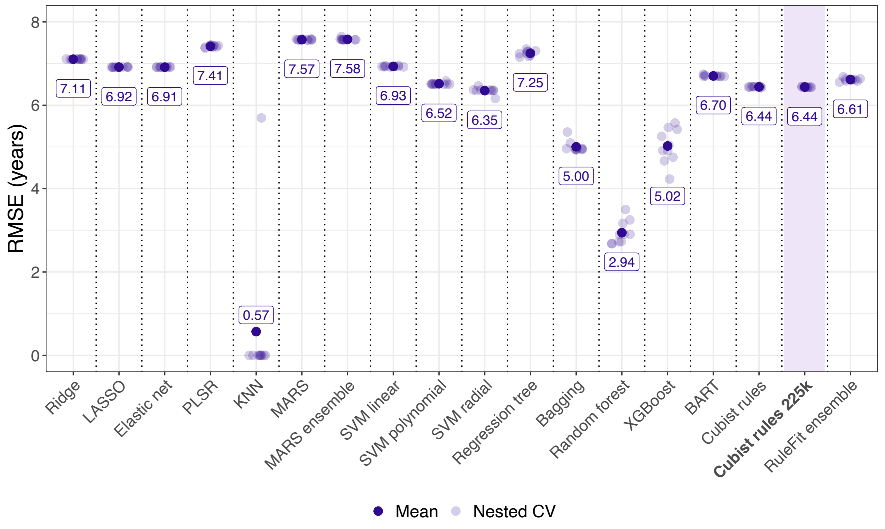

Figure S38. Nested cross-validation root-mean-square error (RMSE) for all models with tuned hyperparameter values in the 90% training sets. See Box 1 for model abbreviations. *N* = 101,359, except for Cubist rules 225k (*N* = 225,212).

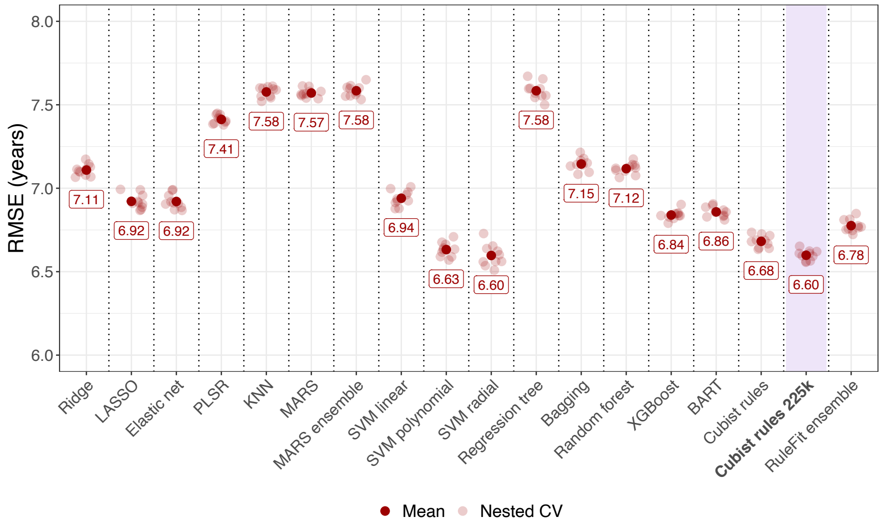

Figure S39. Nested cross-validation root-mean-square error (RMSE) for all models with tuned hyperparameter values in the 10% test sets. See Box 1 for model abbreviations. *N* = 101,359, except for Cubist rules 225k (*N* = 225,212).

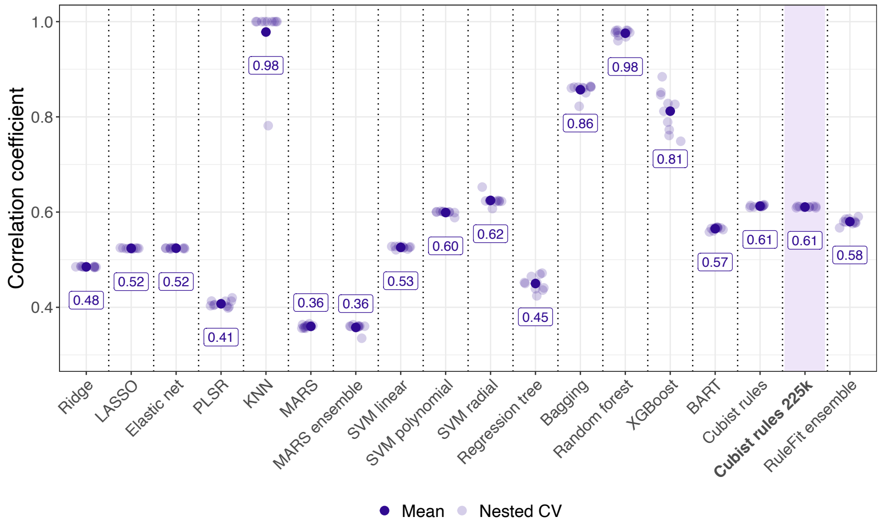

Figure S40. Nested cross-validation Pearson's correlation coefficient (r) for all models with tuned hyperparameter values in the 90% training sets. See Box 1 for model abbreviations. N = 101,359, except for Cubist rules 225k (N = 225,212).

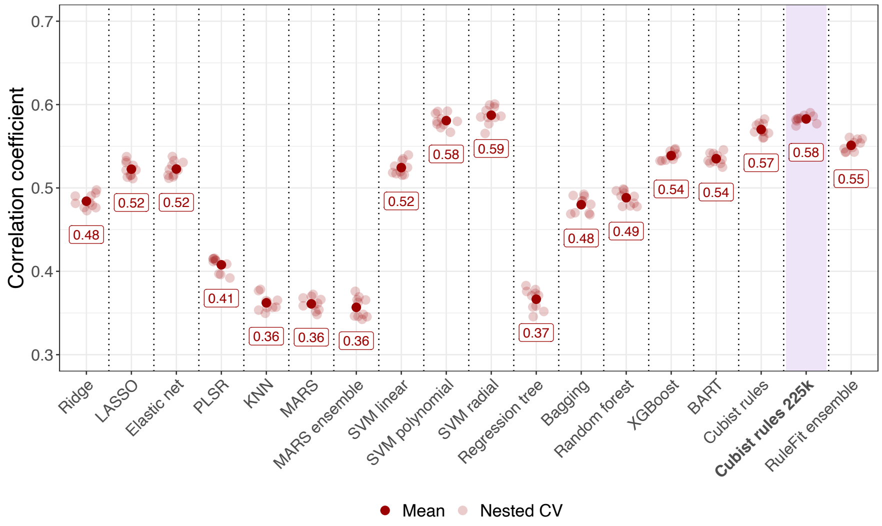

Figure S41. Nested cross-validation Pearson's correlation coefficient (r) for all models with tuned hyperparameter values in the 10% test sets. See Box 1 for model abbreviations. N = 101,359, except for Cubist rules 225k (N = 225,212).

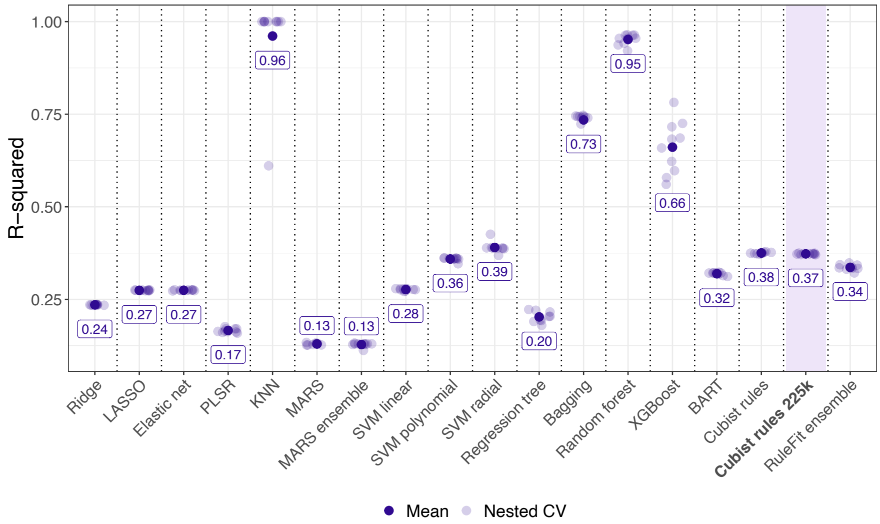

Figure S42. Nested cross-validation coefficient of determination (R^2) for all models with tuned hyperparameter values in the 90% training sets. See Box 1 for model abbreviations. N = 101,359, except for Cubist rules 225k (N = 225,212).

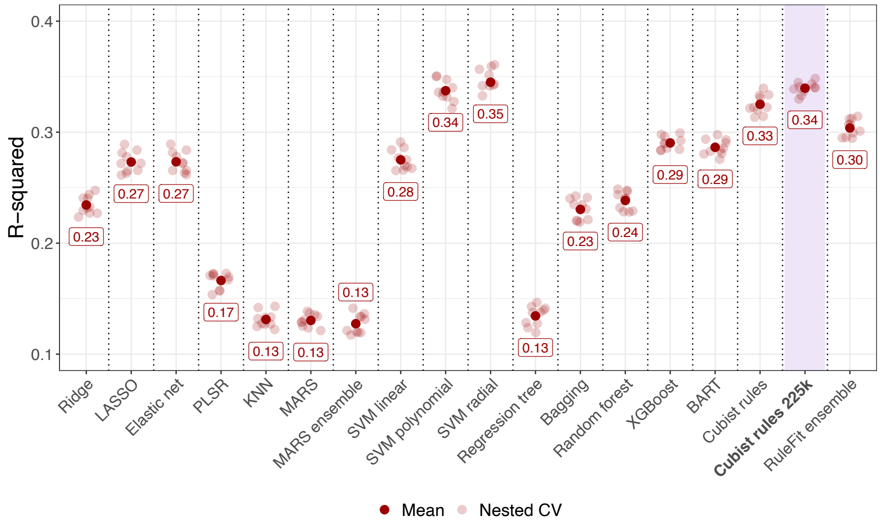

Figure S43. Nested cross-validation coefficient of determination (R^2) for all models with tuned hyperparameter values in the 10% test sets. See Box 1 for model abbreviations. N = 101,359, except for Cubist rules 225k (N = 225,212).

7. Variable importance scores

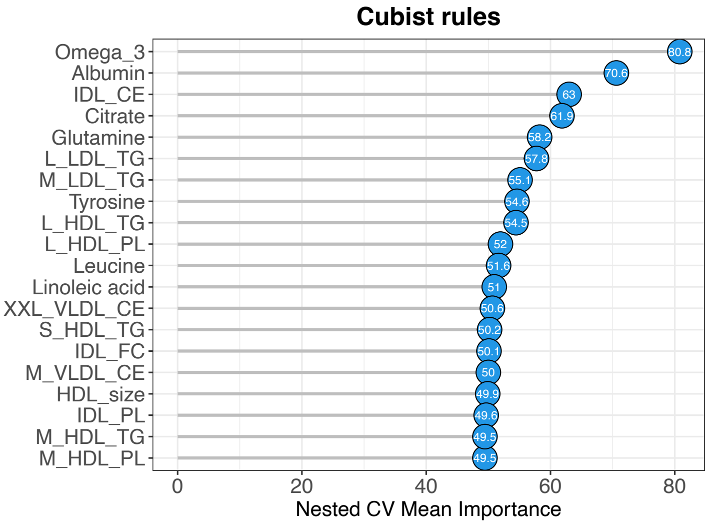

Figure S44. Variable importance plot, showing the top 20 metabolites ranked based on their influence on the Cubist rule-based regression model's predictive accuracy (trained on N = 225,212 participants). Values shown represent averages across the 10 outer loops of the nested cross-validation. IDL = intermediate-density lipoprotein; CE = cholesteryl esters; LDL = low-density lipoprotein; TG = triglycerides; HDL = high-density lipoprotein; PL = phospholipids; VLDL = very low-density lipoprotein; IDL = intermediate-density lipoprotein; FC = free cholesterol; CV = cross-validation.

8. Correlation predicted and chronological age

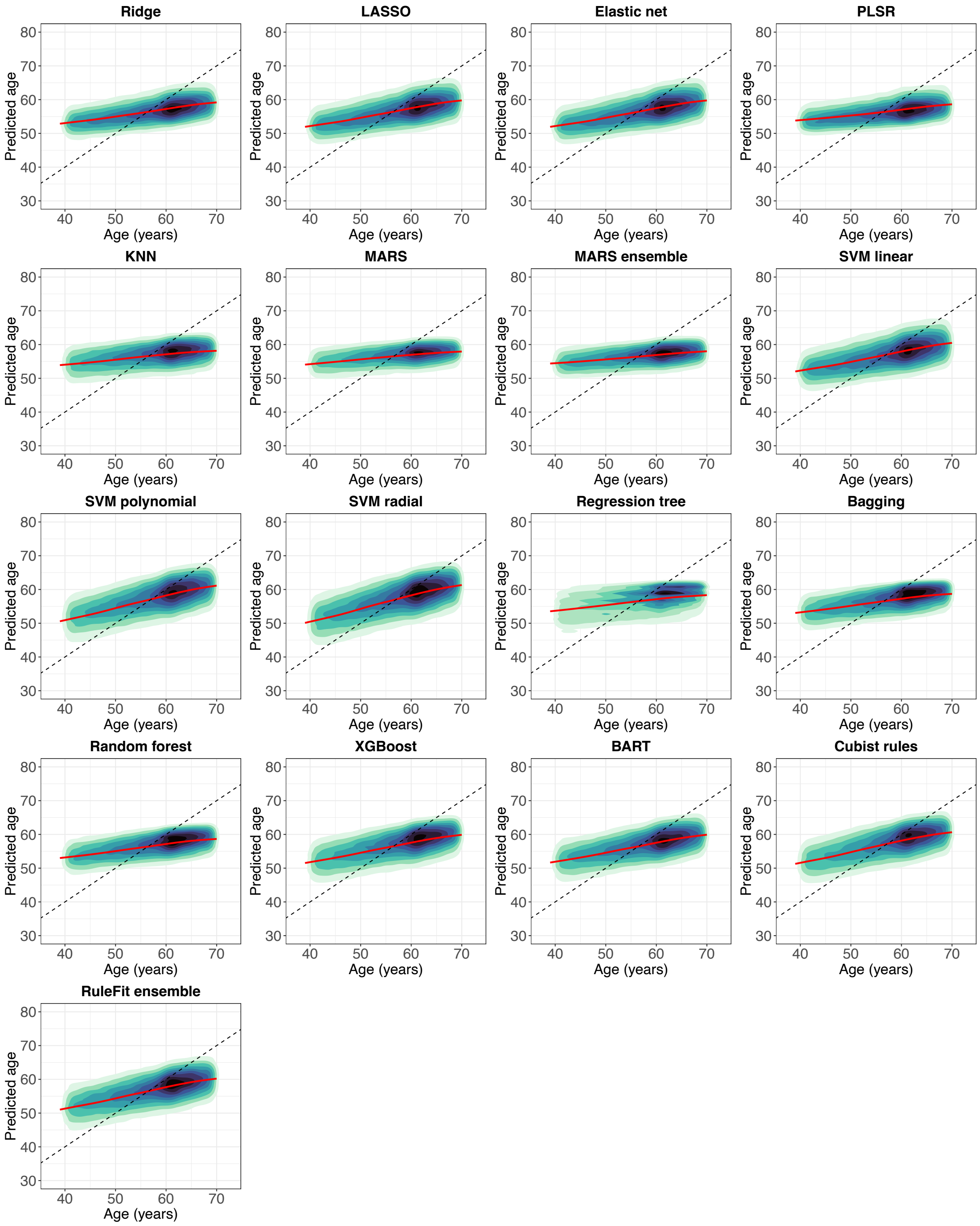

Figure S45. Scatter plots for all models showing the correlation between predicted age and chronological age before applying a statistical correction to the predicted age to remove the age bias (i.e., the systematic overestimation of age in young individuals and underestimation of age in older individuals). See Box 1 for model abbreviations. $N = 101{,}359$.

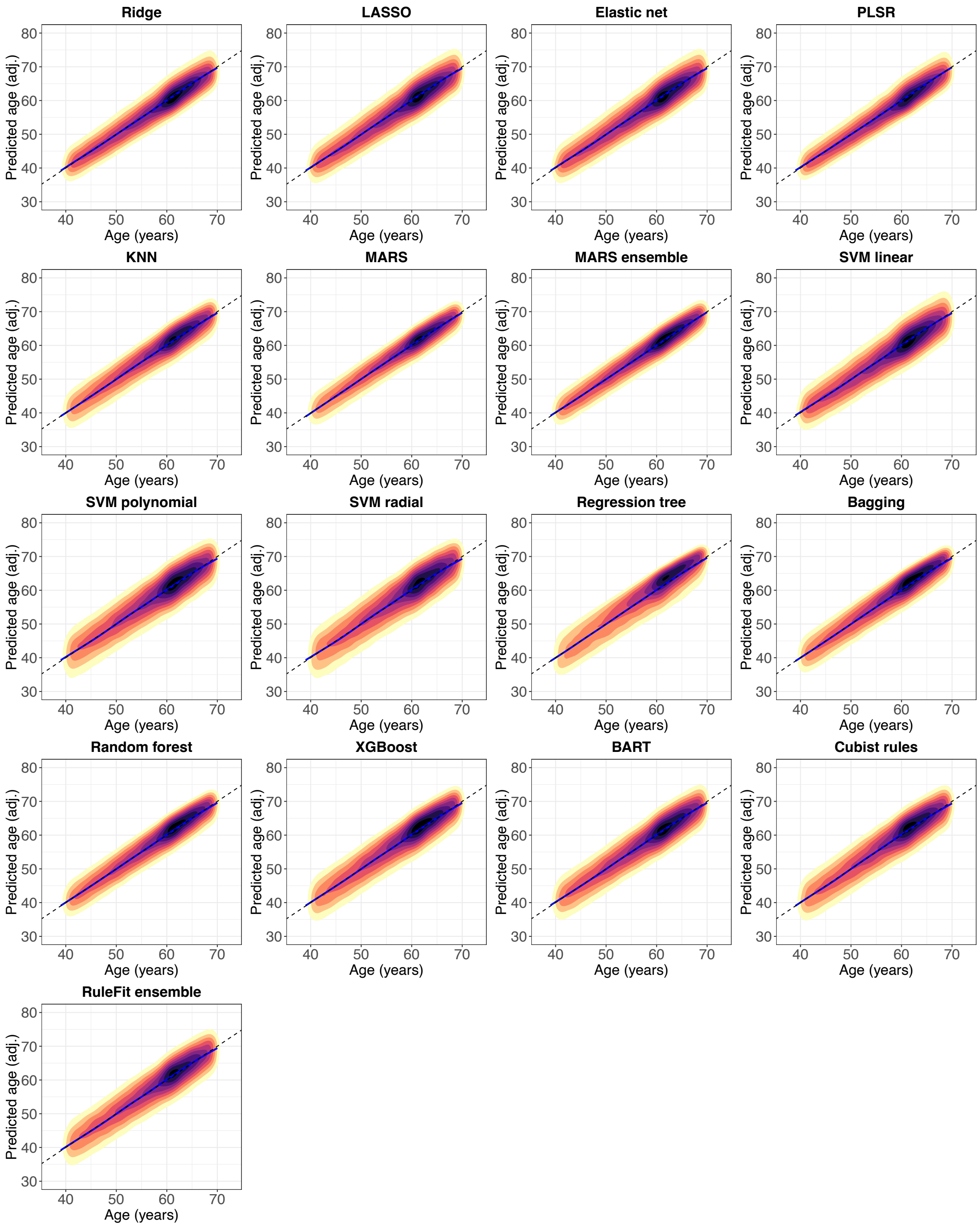

Figure S46. Scatter plots for all models showing the correlation between predicted age and chronological age after applying a statistical correction to the predicted age to remove the age bias (i.e., the systematic overestimation of age in young individuals and underestimation of age in older individuals). See Box 1 for model abbreviations. $N = 101{,}359$.

9. Model extrapolation

Table S4. Model extrapolation beyond chronological age range (39 to 70 years)

	Predicted age				Extrapolation			
	MileAge		MileAge (adj.)		MileAge		MileAge (adj.)	
Model	Min	Max	Min	Max	*n* < Min	*n* > Max	*n* < Min	*n* > Max
Ridge	42.83	77.63	30.78	84.37	0	45	1694	3985
LASSO	37.37	77.76	27.57	82.30	2	111	2072	4533
Elastic net	37.36	77.68	27.78	82.37	2	107	2062	4535
PLSR	43.46	77.62	30.59	84.85	0	43	1397	3870
KNN	43.01	66.42	30.60	76.57	0	0	2240	3217
MARS	38.65	68.23	27.83	76.34	1	0	1921	2561
MARS ensemble	44.30	67.70	30.68	77.50	0	0	1408	2532
SVM linear	38.21	77.51	27.96	84.52	2	250	1887	4136
SVM polynomial	29.17	77.48	20.19	85.15	94	159	3379	4735
SVM radial	31.95	77.51	23.35	84.97	107	149	3570	4812
Regression tree	43.33	65.00	31.10	76.32	0	0	2727	3189
Bagging	45.30	64.63	32.60	74.83	0	0	2101	2683
Random forest	44.80	64.83	32.34	75.11	0	0	1928	2813
XGBoost	38.23	70.45	28.43	79.42	2	3	2777	3876
BART	38.23	74.63	27.26	82.23	2	29	2581	4165
Cubist rules	38.64	74.82	27.63	80.15	2	32	2841	4035
RuleFit ensemble	36.93	75.90	26.28	81.77	24	48	2870	4485

Note: Min = minimum; Max = maximum. See Box 1 for model abbreviations. N = 101,359.

10. Predictive model performance after age-bias correction

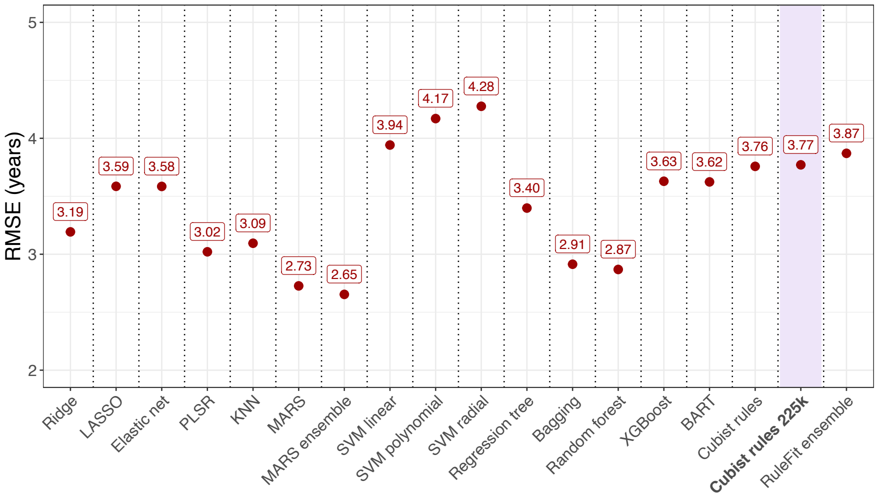

Figure S47. Root-mean-square error (RMSE) for all models with tuned hyperparameter values calculated in the nested cross-validation dataset after applying a statistical correction to the predicted age to remove the age bias (i.e., the systematic overestimation of age in young individuals and underestimation of age in older individuals). See Box 1 for model abbreviations. N = 101,359, except for Cubist rules 225k (N = 225,212).

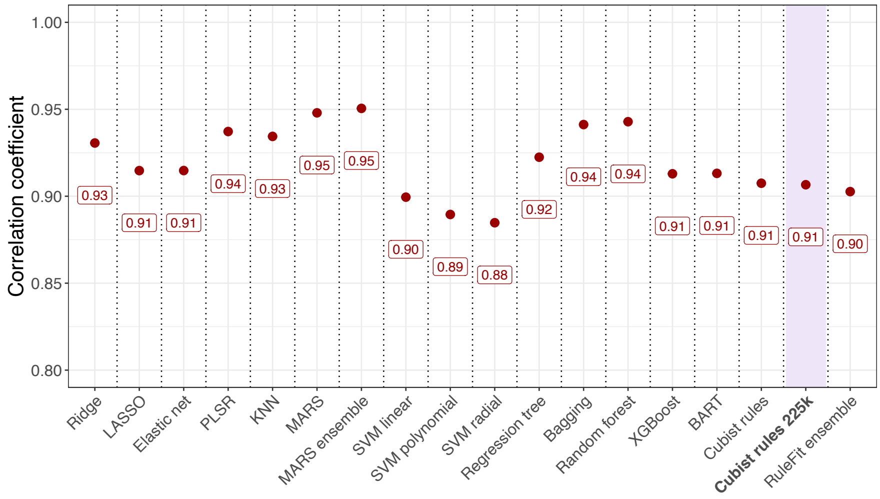

Figure S48. Pearson's correlation coefficient (*r*) for all models with tuned hyperparameter values calculated in the nested cross-validation dataset after applying a statistical correction to the predicted age to remove the age bias (i.e., the systematic overestimation of age in young individuals and underestimation of age in older individuals). See Box 1 for model abbreviations. $N = 101{,}359$, except for Cubist rules 225k ($N = 225{,}212$).

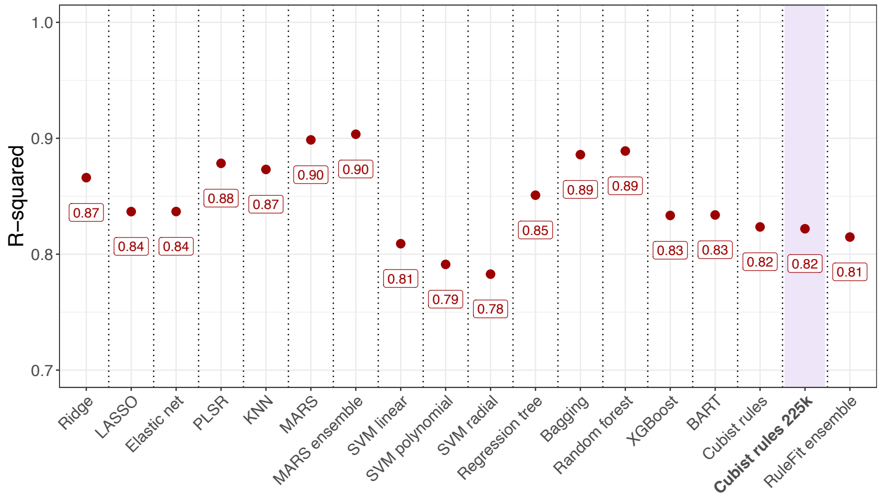

Figure S49. Coefficient of determination (R^2) for all models with tuned hyperparameter values calculated in the nested cross-validation dataset after applying a statistical correction to the predicted age to remove the age bias (i.e., the systematic overestimation of age in young individuals and underestimation of age in older individuals). See Box 1 for model abbreviations. $N = 101{,}359$, except for Cubist rules 225k ($N = 225{,}212$).

11. Descriptive statistics MileAge delta

Table S5. Descriptive statistics age delta before and after age-bias correction

	MileAge delta					MileAge delta (adj.)				
Model	Min	Med	Mean	Max	IQR	Min	Med	Mean	Max	IQR
Ridge	-21.29	-0.62	0.00	30.10	10.74	-12.01	-0.14	0.00	20.20	4.20
LASSO	-24.52	-0.46	0.00	29.71	10.21	-15.68	-0.10	0.00	20.09	4.73
Elastic net	-24.50	-0.46	0.00	29.50	10.21	-15.71	-0.10	0.00	20.01	4.74
PLSR	-20.54	-0.74	0.00	30.08	11.37	-10.41	-0.20	0.00	21.25	3.94
KNN	-21.63	-0.66	0.06	23.58	11.51	-11.79	0.17	0.00	10.90	4.27
MARS	-22.62	-0.85	0.00	24.04	11.68	-16.17	0.16	0.00	11.07	3.63
MARS ensemble	-21.46	-0.88	0.00	25.87	11.75	-11.58	0.09	0.00	11.49	3.62
SVM linear	-24.60	-0.02	0.41	32.11	10.13	-17.03	-0.12	0.00	20.04	5.19
SVM polynomial	-28.12	-0.01	0.37	30.68	9.38	-23.22	0.16	0.00	19.78	5.52
SVM radial	-28.83	-0.03	0.30	27.87	9.17	-22.03	0.17	0.00	18.07	5.73
Regression tree	-26.67	-0.73	0.01	25.00	11.29	-15.35	0.42	0.00	11.27	4.94
Bagging	-23.12	-0.69	0.02	22.78	10.86	-12.24	0.20	0.00	10.35	4.10
Random forest	-23.21	-0.78	-0.07	23.14	10.83	-12.39	0.14	0.00	10.69	4.01
XGBoost	-25.36	-0.51	0.00	25.06	9.89	-15.84	0.19	0.00	14.85	4.95
BART	-24.34	-0.46	0.01	28.67	9.96	-16.23	0.09	0.00	16.90	4.86
Cubist rules	-27.06	-0.01	0.41	27.14	9.55	-18.94	0.14	0.00	16.50	5.10
Cubist rules 225k	-28.80	0.00	0.42	26.53	9.42	-20.85	0.16	0.00	16.21	5.12
RuleFit ensemble	-25.02	-0.41	0.00	26.10	9.68	-18.69	0.12	0.00	16.77	5.19

Note: Min = minimum; Med = median; Max = maximum; IQR = interquartile range. See Box 1 for model abbreviations. N = 101,359, except for Cubist rules 225k (N = 225,212).

12. Pseudo-validation

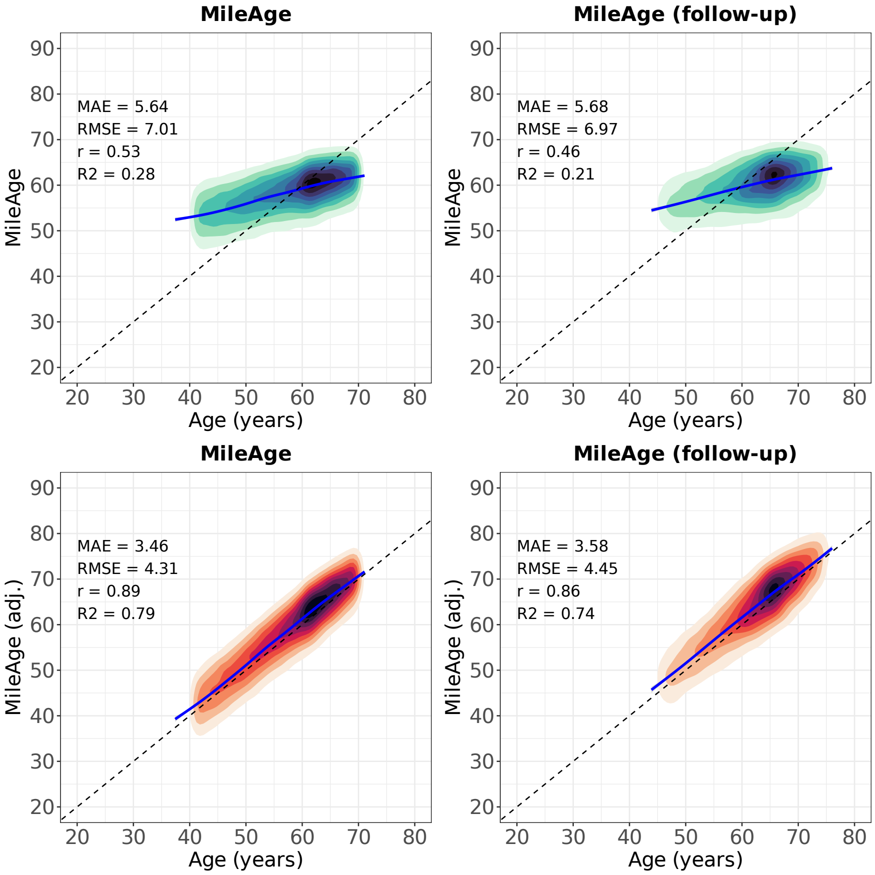

Figure S50. Pseudo-validation of MileAge derived from the Cubist rule-based regression model trained on $N = 101,359$ participants and the age-bias correction: (a) in an additional $N = 129,877$ participants at the baseline assessment; (b) in a subset of $N = 10,618$ participants with repeat assessment data at follow-up. These participants were not included in the original baseline sample that was used for training multiple machine learning models. Scatter plots show the correlation between predicted age and chronological age before and after applying a statistical correction to the predicted age to remove the age bias (i.e., the systematic overestimation of age in young individuals and underestimation of age in older individuals).

13. Sample characteristics health indicators

Table S6. Health indicators by MileAge delta (adj.) for the Cubist rules model

		MileAge delta (adj.)		
	Full sample (*N*=101359)	≤ 1SD below the mean (*n*=16204)	Middle (*n*=69166)	≥ 1SD above the mean (*n*=15989)
Age				
Chronological age	56.44 (8.12)	55.85 (8.33)	56.75 (8.08)	55.69 (8.00)
Predicted age	56.85 (4.57)	50.77 (3.24)	57.04 (3.35)	62.16 (2.80)
Predicted age (adj.)	56.44 (8.95)	49.96 (8.54)	56.84 (8.39)	61.24 (7.93)
MileAge delta (adj.)	0.00 (3.76)	-5.89 (1.79)	0.10 (2.04)	5.55 (1.53)
Health indicators				
Telomere length[1]	0.00 (1.00)	-0.03 (0.99)	0.00 (1.00)	0.01 (1.00)
Frailty index[2]	0.12 (0.07)	0.11 (0.07)	0.12 (0.07)	0.14 (0.08)
Frailty phenotype				
Non-frail	50531 (49.9%)	8344 (51.5%)	34902 (50.5%)	7285 (45.6%)
Pre-frail	41718 (41.2%)	6561 (40.5%)	28195 (40.8%)	6962 (43.5%)
Frail	2812 (2.8%)	340 (2.1%)	1805 (2.6%)	667 (4.2%)
Missing	6298 (6.2%)	959 (5.9%)	4264 (6.2%)	1075 (6.7%)
Long-standing illness				
No	67462 (66.6%)	11502 (71.0%)	46568 (67.3%)	9392 (58.7%)
Yes	31288 (30.9%)	4326 (26.7%)	20794 (30.1%)	6168 (38.6%)
Missing	2609 (2.6%)	376 (2.3%)	1804 (2.6%)	429 (2.7%)
Health status				
Healthy	67275 (66.4%)	11496 (70.9%)	46331 (67.0%)	9448 (59.1%)
Unhealthy	31545 (31.1%)	4287 (26.5%)	21086 (30.5%)	6172 (38.6%)
Missing	2539 (2.5%)	421 (2.6%)	1749 (2.5%)	369 (2.3%)
Self-rated health				
Excellent	16717 (16.5%)	3060 (18.9%)	11493 (16.6%)	2164 (13.5%)
Good	59059 (58.3%)	9662 (59.6%)	40730 (58.9%)	8667 (54.2%)
Fair	20860 (20.6%)	2909 (18.0%)	14001 (20.2%)	3950 (24.7%)
Poor	4153 (4.1%)	482 (3.0%)	2570 (3.7%)	1101 (6.9%)
Missing	570 (0.6%)	91 (0.6%)	372 (0.5%)	107 (0.7%)

Note: SD = standard deviation. MileAge delta (adj.) derived from the Cubist rule-based regression model (trained on N = 101,359 participants). Numbers correspond to mean (standard deviation) or count (percentage). [1]n = 3395 missing. [2]n = 330 missing.

14. Associations between MileAge delta and health indicators

Table S7. Association between frailty index scores and MileAge delta (adj.)

Model	Level	*β*	95% CI		*p*
SVM radial	≤ X̄-1SD		Reference		
	Middle	0.006	0.005	0.007	<0.001
	≥ X̄+1SD	0.020	0.019	0.022	<0.001
SVM polynomial	≤ X̄-1SD		Reference		
	Middle	0.006	0.005	0.007	<0.001
	≥ X̄+1SD	0.021	0.020	0.023	<0.001
Cubist rules	≤ X̄-1SD		Reference		
	Middle	0.006	0.005	0.008	<0.001
	≥ X̄+1SD	0.023	0.021	0.024	<0.001
RuleFit ensemble	≤ X̄-1SD		Reference		
	Middle	0.005	0.003	0.006	<0.001
	≥ X̄+1SD	0.019	0.017	0.021	<0.001
XGBoost	≤ X̄-1SD		Reference		
	Middle	0.007	0.006	0.009	<0.001
	≥ X̄+1SD	0.021	0.019	0.023	<0.001
BART	≤ X̄-1SD		Reference		
	Middle	0.006	0.005	0.008	<0.001
	≥ X̄+1SD	0.020	0.019	0.022	<0.001
SVM linear	≤ X̄-1SD		Reference		
	Middle	0.006	0.004	0.007	<0.001
	≥ X̄+1SD	0.021	0.019	0.023	<0.001
Elastic net	≤ X̄-1SD		Reference		
	Middle	0.005	0.004	0.006	<0.001
	≥ X̄+1SD	0.019	0.017	0.021	<0.001
LASSO	≤ X̄-1SD		Reference		
	Middle	0.005	0.004	0.006	<0.001
	≥ X̄+1SD	0.019	0.018	0.021	<0.001
Ridge	≤ X̄-1SD		Reference		
	Middle	0.006	0.005	0.007	<0.001
	≥ X̄+1SD	0.019	0.018	0.021	<0.001
Random forest	≤ X̄-1SD		Reference		
	Middle	0.010	0.009	0.011	<0.001
	≥ X̄+1SD	0.019	0.018	0.021	<0.001
Bagging	≤ X̄-1SD		Reference		
	Middle	0.009	0.008	0.01	<0.001
	≥ X̄+1SD	0.02	0.019	0.022	<0.001
PLSR	≤ X̄-1SD		Reference		
	Middle	0.008	0.007	0.009	<0.001
	≥ X̄+1SD	0.020	0.018	0.021	<0.001
Regression tree	≤ X̄-1SD		Reference		
	Middle	0.005	0.004	0.006	<0.001
	≥ X̄+1SD	0.017	0.015	0.018	<0.001
KNN	≤ X̄-1SD		Reference		
	Middle	0.008	0.007	0.009	<0.001
	≥ X̄+1SD	0.020	0.018	0.021	<0.001

MARS	≤ $\bar{X}$-1SD		Reference		
	Middle	0.001	0.001	0.003	0.025
	≥ $\bar{X}$+1SD	0.008	0.006	0.01	<0.001
MARS ensemble	≤ $\bar{X}$-1SD		Reference		
	Middle	0.002	0.001	0.003	0.003
	≥ $\bar{X}$+1SD	0.012	0.011	0.014	<0.001

Note: CI = confidence interval; SD = standard deviation. Models were adjusted for chronological age and sex. See Box 1 for model abbreviations. N = 101,029.

Table S8. Association between the frailty phenotype and MileAge delta (adj.)

Model	Level	OR	95% CI		*p*
SVM radial	≤ $\bar{X}$-1SD		Reference		
	Middle	1.01	0.97	1.05	0.608
	≥ $\bar{X}$+1SD	1.20	1.14	1.25	<0.001
SVM polynomial	≤ $\bar{X}$-1SD		Reference		
	Middle	1.03	0.99	1.07	0.122
	≥ $\bar{X}$+1SD	1.25	1.19	1.31	<0.001
Cubist rules	≤ $\bar{X}$-1SD		Reference		
	Middle	1.05	1.01	1.09	0.008
	≥ $\bar{X}$+1SD	1.29	1.23	1.35	<0.001
RuleFit ensemble	≤ $\bar{X}$-1SD		Reference		
	Middle	1.03	1.00	1.07	0.055
	≥ $\bar{X}$+1SD	1.24	1.18	1.29	<0.001
XGBoost	≤ $\bar{X}$-1SD		Reference		
	Middle	1.06	1.02	1.09	0.002
	≥ $\bar{X}$+1SD	1.25	1.19	1.31	<0.001
BART	≤ $\bar{X}$-1SD		Reference		
	Middle	1.06	1.02	1.09	0.003
	≥ $\bar{X}$+1SD	1.28	1.23	1.34	<0.001
SVM linear	≤ $\bar{X}$-1SD		Reference		
	Middle	1.06	1.02	1.11	0.003
	≥ $\bar{X}$+1SD	1.29	1.23	1.36	<0.001
Elastic net	≤ $\bar{X}$-1SD		Reference		
	Middle	1.04	1.00	1.08	0.037
	≥ $\bar{X}$+1SD	1.26	1.21	1.32	<0.001
LASSO	≤ $\bar{X}$-1SD		Reference		
	Middle	1.04	1.00	1.08	0.036
	≥ $\bar{X}$+1SD	1.27	1.21	1.33	<0.001
Ridge	≤ $\bar{X}$-1SD		Reference		
	Middle	1.05	1.02	1.09	0.003
	≥ $\bar{X}$+1SD	1.25	1.20	1.31	<0.001
Random forest	≤ $\bar{X}$-1SD		Reference		
	Middle	1.10	1.07	1.14	<0.001
	≥ $\bar{X}$+1SD	1.20	1.15	1.25	<0.001
Bagging	≤ $\bar{X}$-1SD		Reference		
	Middle	1.10	1.06	1.14	<0.001
	≥ $\bar{X}$+1SD	1.21	1.15	1.26	<0.001
PLSR	≤ $\bar{X}$-1SD		Reference		
	Middle	1.09	1.05	1.13	<0.001
	≥ $\bar{X}$+1SD	1.27	1.21	1.33	<0.001
Regression tree	≤ $\bar{X}$-1SD		Reference		
	Middle	1.04	1.01	1.08	0.014
	≥ $\bar{X}$+1SD	1.20	1.15	1.25	<0.001
KNN	≤ $\bar{X}$-1SD		Reference		
	Middle	1.04	1.00	1.08	0.031
	≥ $\bar{X}$+1SD	1.17	1.12	1.22	<0.001
MARS	≤ $\bar{X}$-1SD		Reference		

	Middle	0.99	0.96	1.03	0.667
	≥ X̄+1SD	1.12	1.07	1.17	<0.001
MARS ensemble	≤ X̄-1SD		Reference		
	Middle	0.97	0.94	1.01	0.122
	≥ X̄+1SD	1.14	1.08	1.19	<0.001

Note: OR = odds ratio; CI = confidence interval; SD = standard deviation. Proportional odds ratios indicate the changes in odds of being frailer associated with the MileAge delta (adj.) level relative to the reference group. Models were adjusted for chronological age and sex. See Box 1 for model abbreviations. *N* = 95,061.

Table S9. Association between telomere length and MileAge delta (adj.)

Model	Level	*β*	95% CI		*p*
SVM radial	≤ X̄-1SD		Reference		
	Middle	0.031	0.013	0.048	<0.001
	≥ X̄+1SD	0.055	0.034	0.077	<0.001
SVM polynomial	≤ X̄-1SD		Reference		
	Middle	0.031	0.013	0.048	<0.001
	≥ X̄+1SD	0.057	0.035	0.079	<0.001
Cubist rules	≤ X̄-1SD		Reference		
	Middle	0.015	-0.002	0.032	0.088
	≥ X̄+1SD	0.052	0.030	0.073	<0.001
RuleFit ensemble	≤ X̄-1SD		Reference		
	Middle	0.021	0.004	0.038	0.013
	≥ X̄+1SD	0.052	0.031	0.074	<0.001
XGBoost	≤ X̄-1SD		Reference		
	Middle	0.024	0.007	0.041	0.006
	≥ X̄+1SD	0.050	0.029	0.072	<0.001
BART	≤ X̄-1SD		Reference		
	Middle	0.021	0.004	0.038	0.015
	≥ X̄+1SD	0.052	0.031	0.074	<0.001
SVM linear	≤ X̄-1SD		Reference		
	Middle	0.014	-0.005	0.033	0.162
	≥ X̄+1SD	0.035	0.011	0.060	0.005
Elastic net	≤ X̄-1SD		Reference		
	Middle	0.022	0.005	0.040	0.011
	≥ X̄+1SD	0.052	0.030	0.074	<0.001
LASSO	≤ X̄-1SD		Reference		
	Middle	0.022	0.005	0.039	0.013
	≥ X̄+1SD	0.052	0.030	0.074	<0.001
Ridge	≤ X̄-1SD		Reference		
	Middle	0.033	0.016	0.050	<0.001
	≥ X̄+1SD	0.052	0.030	0.074	<0.001
Random forest	≤ X̄-1SD		Reference		
	Middle	0.009	-0.008	0.026	0.292
	≥ X̄+1SD	0.020	-0.001	0.042	0.062
Bagging	≤ X̄-1SD		Reference		
	Middle	0.008	-0.009	0.025	0.357
	≥ X̄+1SD	0.020	-0.002	0.041	0.071
PLSR	≤ X̄-1SD		Reference		
	Middle	0.029	0.011	0.046	0.001
	≥ X̄+1SD	0.036	0.013	0.058	0.002
Regression tree	≤ X̄-1SD		Reference		
	Middle	0.001	-0.015	0.018	0.872
	≥ X̄+1SD	0.014	-0.007	0.036	0.177
KNN	≤ X̄-1SD		Reference		
	Middle	0.005	-0.012	0.022	0.563
	≥ X̄+1SD	0.030	0.009	0.051	0.006
MARS	≤ X̄-1SD		Reference		

	Middle	-0.018	-0.035	-0.001	0.041
	≥ X̄+1SD	-0.010	-0.032	0.012	0.369
MARS ensemble	≤ X̄-1SD		Reference		
	Middle	-0.015	-0.033	0.003	0.095
	≥ X̄+1SD	-0.020	-0.043	0.003	0.084

Note: CI = confidence interval; SD = standard deviation. Models were adjusted for chronological age and sex. See Box 1 for model abbreviations. *N* = 97,964

Table S10. Association between long-standing illness and MileAge delta (adj.)

Model	Level	OR	95% CI		*p*
SVM radial	≤ X̄-1SD		Reference		
	Middle	1.14	1.10	1.19	<0.001
	≥ X̄+1SD	1.71	1.63	1.80	<0.001
SVM polynomial	≤ X̄-1SD		Reference		
	Middle	1.13	1.09	1.18	<0.001
	≥ X̄+1SD	1.75	1.66	1.83	<0.001
Cubist rules	≤ X̄-1SD		Reference		
	Middle	1.17	1.12	1.21	<0.001
	≥ X̄+1SD	1.82	1.73	1.91	<0.001
RuleFit ensemble	≤ X̄-1SD		Reference		
	Middle	1.10	1.06	1.15	<0.001
	≥ X̄+1SD	1.65	1.57	1.73	<0.001
XGBoost	≤ X̄-1SD		Reference		
	Middle	1.17	1.13	1.22	<0.001
	≥ X̄+1SD	1.75	1.67	1.84	<0.001
BART	≤ X̄-1SD		Reference		
	Middle	1.17	1.13	1.22	<0.001
	≥ X̄+1SD	1.69	1.61	1.78	<0.001
SVM linear	≤ X̄-1SD		Reference		
	Middle	1.12	1.07	1.17	<0.001
	≥ X̄+1SD	1.62	1.54	1.71	<0.001
Elastic net	≤ X̄-1SD		Reference		
	Middle	1.11	1.07	1.16	<0.001
	≥ X̄+1SD	1.61	1.53	1.69	<0.001
LASSO	≤ X̄-1SD		Reference		
	Middle	1.11	1.07	1.16	<0.001
	≥ X̄+1SD	1.61	1.54	1.69	<0.001
Ridge	≤ X̄-1SD		Reference		
	Middle	1.13	1.09	1.18	<0.001
	≥ X̄+1SD	1.61	1.53	1.69	<0.001
Random forest	≤ X̄-1SD		Reference		
	Middle	1.25	1.21	1.30	<0.001
	≥ X̄+1SD	1.66	1.58	1.74	<0.001
Bagging	≤ X̄-1SD		Reference		
	Middle	1.22	1.17	1.27	<0.001
	≥ X̄+1SD	1.67	1.59	1.75	<0.001
PLSR	≤ X̄-1SD		Reference		
	Middle	1.18	1.13	1.23	<0.001
	≥ X̄+1SD	1.64	1.56	1.72	<0.001
Regression tree	≤ X̄-1SD		Reference		
	Middle	1.09	1.05	1.14	<0.001
	≥ X̄+1SD	1.52	1.45	1.59	<0.001
KNN	≤ X̄-1SD		Reference		
	Middle	1.19	1.14	1.24	<0.001
	≥ X̄+1SD	1.63	1.55	1.71	<0.001
MARS	≤ X̄-1SD		Reference		

	Middle	1.00	0.96	1.04	0.914
	≥ X̄+1SD	1.23	1.17	1.29	<0.001
MARS ensemble	≤ X̄-1SD		Reference		
	Middle	1.05	1.01	1.10	0.013
	≥ X̄+1SD	1.43	1.36	1.51	<0.001

Note: OR = odds ratio; CI = confidence interval; SD = standard deviation. Models were adjusted for chronological age and sex. See Box 1 for model abbreviations. *N* = 98,750.

Table S11. Association between health status and MileAge delta (adj.)

Model	Level	OR	95% CI		*p*
SVM radial	≤ X̄-1SD		Reference		
	Middle	1.17	1.12	1.22	<0.001
	≥ X̄+1SD	1.79	1.70	1.88	<0.001
SVM polynomial	≤ X̄-1SD		Reference		
	Middle	1.14	1.10	1.19	<0.001
	≥ X̄+1SD	1.79	1.71	1.88	<0.001
Cubist rules	≤ X̄-1SD		Reference		
	Middle	1.18	1.14	1.23	<0.001
	≥ X̄+1SD	1.85	1.76	1.94	<0.001
RuleFit ensemble	≤ X̄-1SD		Reference		
	Middle	1.10	1.05	1.14	<0.001
	≥ X̄+1SD	1.70	1.62	1.79	<0.001
XGBoost	≤ X̄-1SD		Reference		
	Middle	1.14	1.10	1.19	<0.001
	≥ X̄+1SD	1.77	1.68	1.85	<0.001
BART	≤ X̄-1SD		Reference		
	Middle	1.17	1.12	1.22	<0.001
	≥ X̄+1SD	1.75	1.67	1.84	<0.001
SVM linear	≤ X̄-1SD		Reference		
	Middle	1.11	1.06	1.16	<0.001
	≥ X̄+1SD	1.59	1.51	1.68	<0.001
Elastic net	≤ X̄-1SD		Reference		
	Middle	1.13	1.09	1.18	<0.001
	≥ X̄+1SD	1.60	1.52	1.68	<0.001
LASSO	≤ X̄-1SD		Reference		
	Middle	1.13	1.08	1.17	<0.001
	≥ X̄+1SD	1.60	1.53	1.68	<0.001
Ridge	≤ X̄-1SD		Reference		
	Middle	1.10	1.06	1.15	<0.001
	≥ X̄+1SD	1.57	1.50	1.65	<0.001
Random forest	≤ X̄-1SD		Reference		
	Middle	1.21	1.16	1.25	<0.001
	≥ X̄+1SD	1.66	1.58	1.74	<0.001
Bagging	≤ X̄-1SD		Reference		
	Middle	1.17	1.12	1.21	<0.001
	≥ X̄+1SD	1.66	1.58	1.74	<0.001
PLSR	≤ X̄-1SD		Reference		
	Middle	1.19	1.15	1.24	<0.001
	≥ X̄+1SD	1.64	1.56	1.73	<0.001
Regression tree	≤ X̄-1SD		Reference		
	Middle	1.06	1.02	1.10	0.002
	≥ X̄+1SD	1.53	1.46	1.60	<0.001
KNN	≤ X̄-1SD		Reference		
	Middle	1.15	1.11	1.19	<0.001
	≥ X̄+1SD	1.66	1.59	1.74	<0.001
MARS	≤ X̄-1SD		Reference		

	Middle	0.97	0.93	1.00	0.073
	≥ $\bar{X}$+1SD	1.11	1.05	1.16	<0.001
MARS ensemble	≤ $\bar{X}$-1SD		Reference		
	Middle	1.04	1.00	1.08	0.052
	≥ $\bar{X}$+1SD	1.40	1.33	1.47	<0.001

Note: OR = odds ratio; CI = confidence interval; SD = standard deviation. Models were adjusted for chronological age and sex. See Box 1 for model abbreviations. *N* = 98,820.

Table S12. Association between self-rated health and MileAge delta (adj.)

Model	Level	OR	95% CI		*p*
SVM radial	≤ X̄-1SD		Reference		
	Middle	1.16	1.12	1.20	<0.001
	≥ X̄+1SD	1.62	1.55	1.69	<0.001
SVM polynomial	≤ X̄-1SD		Reference		
	Middle	1.18	1.14	1.22	<0.001
	≥ X̄+1SD	1.67	1.60	1.74	<0.001
Cubist rules	≤ X̄-1SD		Reference		
	Middle	1.18	1.14	1.22	<0.001
	≥ X̄+1SD	1.72	1.65	1.80	<0.001
RuleFit ensemble	≤ X̄-1SD		Reference		
	Middle	1.15	1.11	1.19	<0.001
	≥ X̄+1SD	1.59	1.52	1.66	<0.001
XGBoost	≤ X̄-1SD		Reference		
	Middle	1.22	1.18	1.26	<0.001
	≥ X̄+1SD	1.66	1.59	1.73	<0.001
BART	≤ X̄-1SD		Reference		
	Middle	1.21	1.17	1.25	<0.001
	≥ X̄+1SD	1.65	1.58	1.72	<0.001
SVM linear	≤ X̄-1SD		Reference		
	Middle	1.19	1.14	1.23	<0.001
	≥ X̄+1SD	1.69	1.61	1.78	<0.001
Elastic net	≤ X̄-1SD		Reference		
	Middle	1.15	1.11	1.19	<0.001
	≥ X̄+1SD	1.61	1.54	1.68	<0.001
LASSO	≤ X̄-1SD		Reference		
	Middle	1.14	1.11	1.18	<0.001
	≥ X̄+1SD	1.61	1.54	1.68	<0.001
Ridge	≤ X̄-1SD		Reference		
	Middle	1.18	1.14	1.23	<0.001
	≥ X̄+1SD	1.64	1.57	1.72	<0.001
Random forest	≤ X̄-1SD		Reference		
	Middle	1.32	1.28	1.36	<0.001
	≥ X̄+1SD	1.61	1.54	1.68	<0.001
Bagging	≤ X̄-1SD		Reference		
	Middle	1.30	1.26	1.35	<0.001
	≥ X̄+1SD	1.66	1.59	1.73	<0.001
PLSR	≤ X̄-1SD		Reference		
	Middle	1.25	1.21	1.30	<0.001
	≥ X̄+1SD	1.71	1.63	1.78	<0.001
Regression tree	≤ X̄-1SD		Reference		
	Middle	1.19	1.15	1.23	<0.001
	≥ X̄+1SD	1.53	1.47	1.60	<0.001
KNN	≤ X̄-1SD		Reference		
	Middle	1.22	1.18	1.26	<0.001
	≥ X̄+1SD	1.60	1.53	1.67	<0.001
MARS	≤ X̄-1SD		Reference		

	Middle	1.08	1.05	1.12	<0.001
	≥ X̄+1SD	1.34	1.29	1.40	<0.001
MARS ensemble	≤ X̄-1SD			Reference	
	Middle	1.05	1.01	1.08	0.014
	≥ X̄+1SD	1.35	1.29	1.41	<0.001

Note: OR = odds ratio; CI = confidence interval; SD = standard deviation. Proportional odds ratios indicate the changes in odds of reporting worse self-rated health associated with the MileAge delta (adj.) level relative to the reference group. Models were adjusted for chronological age and sex. See Box 1 for model abbreviations. N = 100,789.

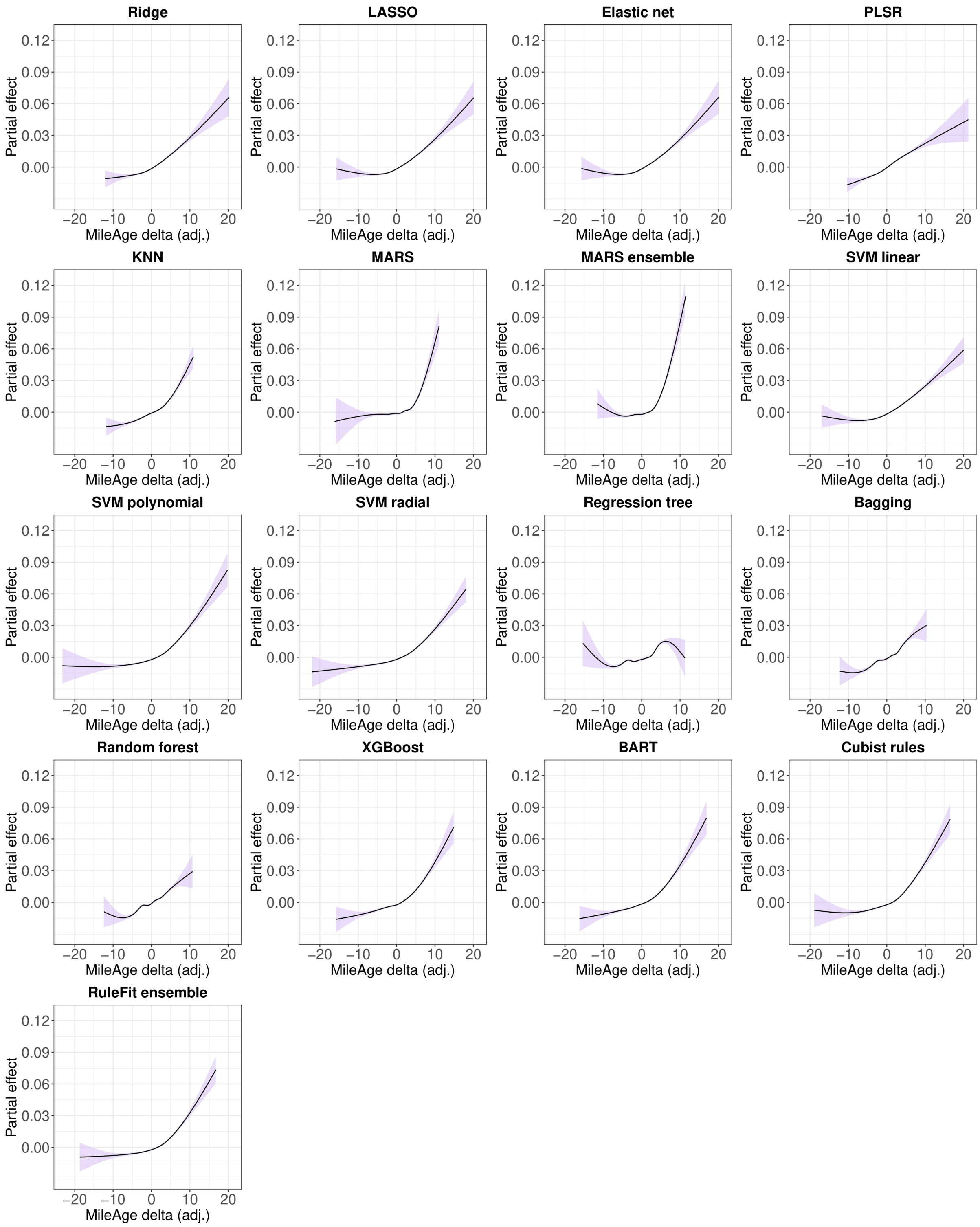

Figure S51. Partial effect plots of generalised additive models of the association between the frailty index and MileAge delta (adj.) for all models. Models were adjusted for chronological age and sex. The shaded areas correspond to 95% confidence intervals. See Box 1 for model abbreviations. *N* = 101,029.

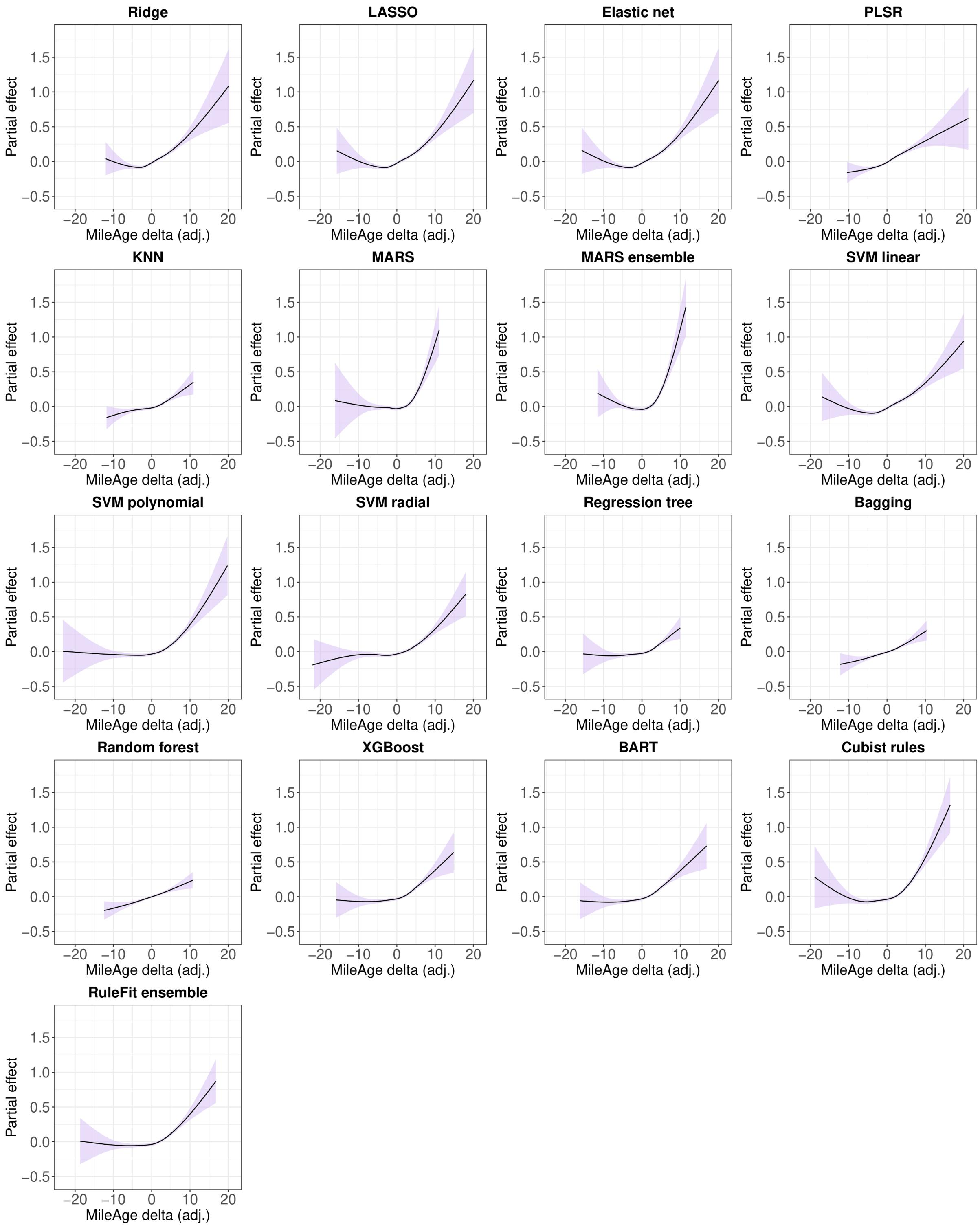

Figure S52. Partial effect plots of generalised additive models of the association between the frailty phenotype and MileAge delta (adj.) for all models. Models were adjusted for chronological age and sex. The shaded areas correspond to 95% confidence intervals. See Box 1 for model abbreviations. $N = 95{,}061$.

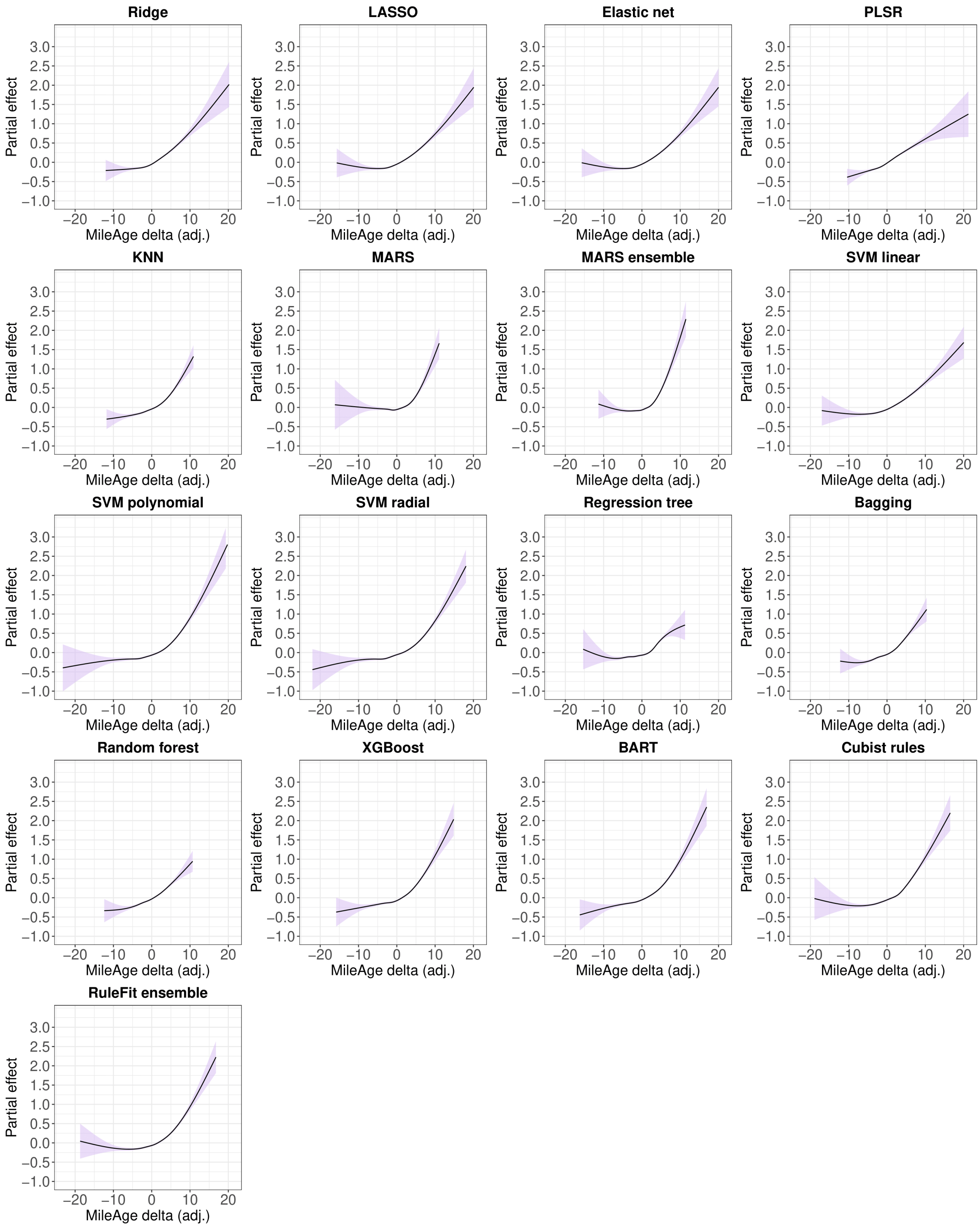

Figure S53. Partial effect plots of generalised additive models of the association between having a long-standing illness and MileAge delta (adj.) for all models. Models were adjusted for chronological age and sex. The shaded areas correspond to 95% confidence intervals. See Box 1 for model abbreviations. $N = 98{,}750$.

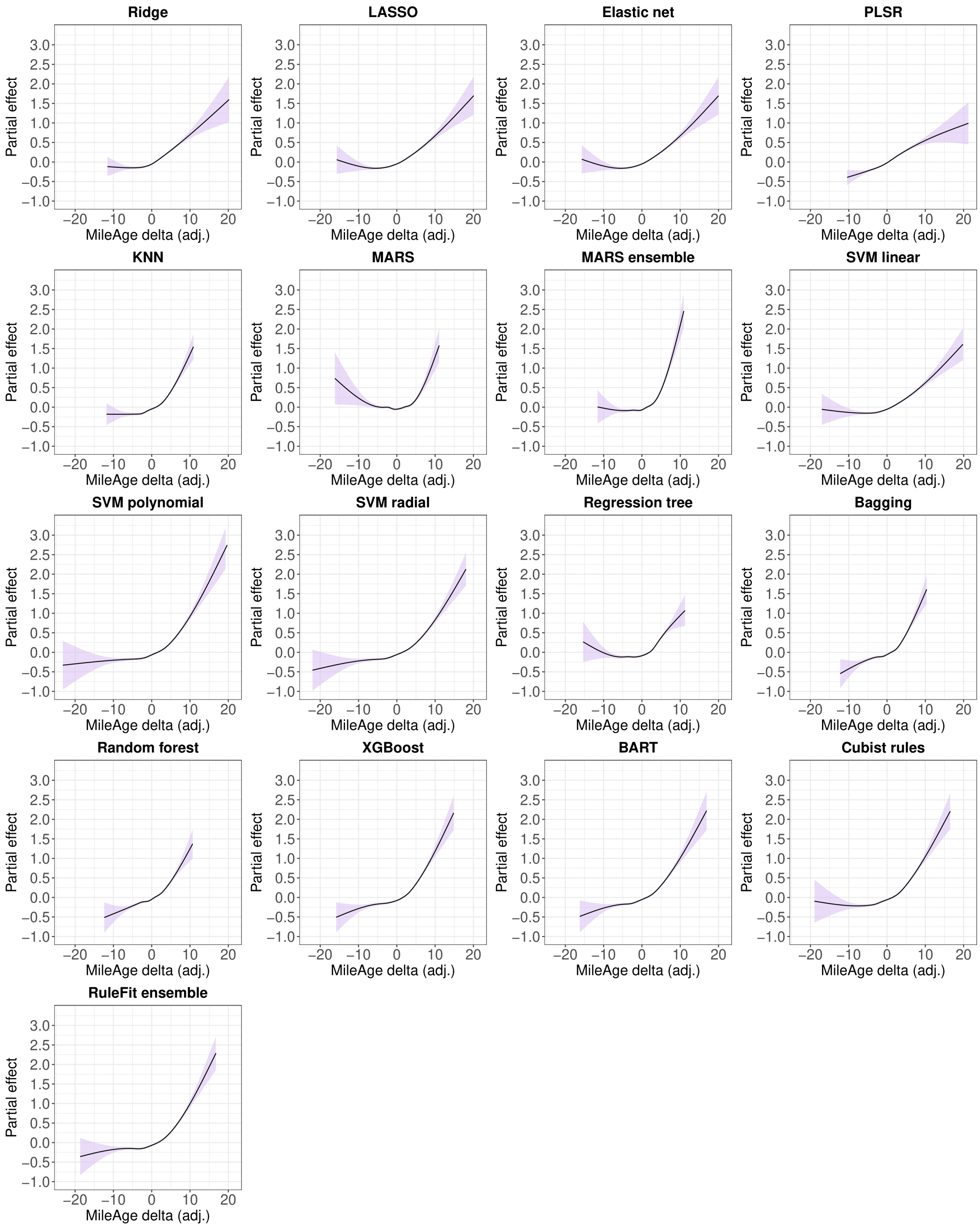

Figure S54. Partial effect plots of generalised additive models of the association between health status and MileAge delta (adj.) for all models. Models were adjusted for chronological age and sex. The shaded areas correspond to 95% confidence intervals. See Box 1 for model abbreviations. $N = 98{,}820$.

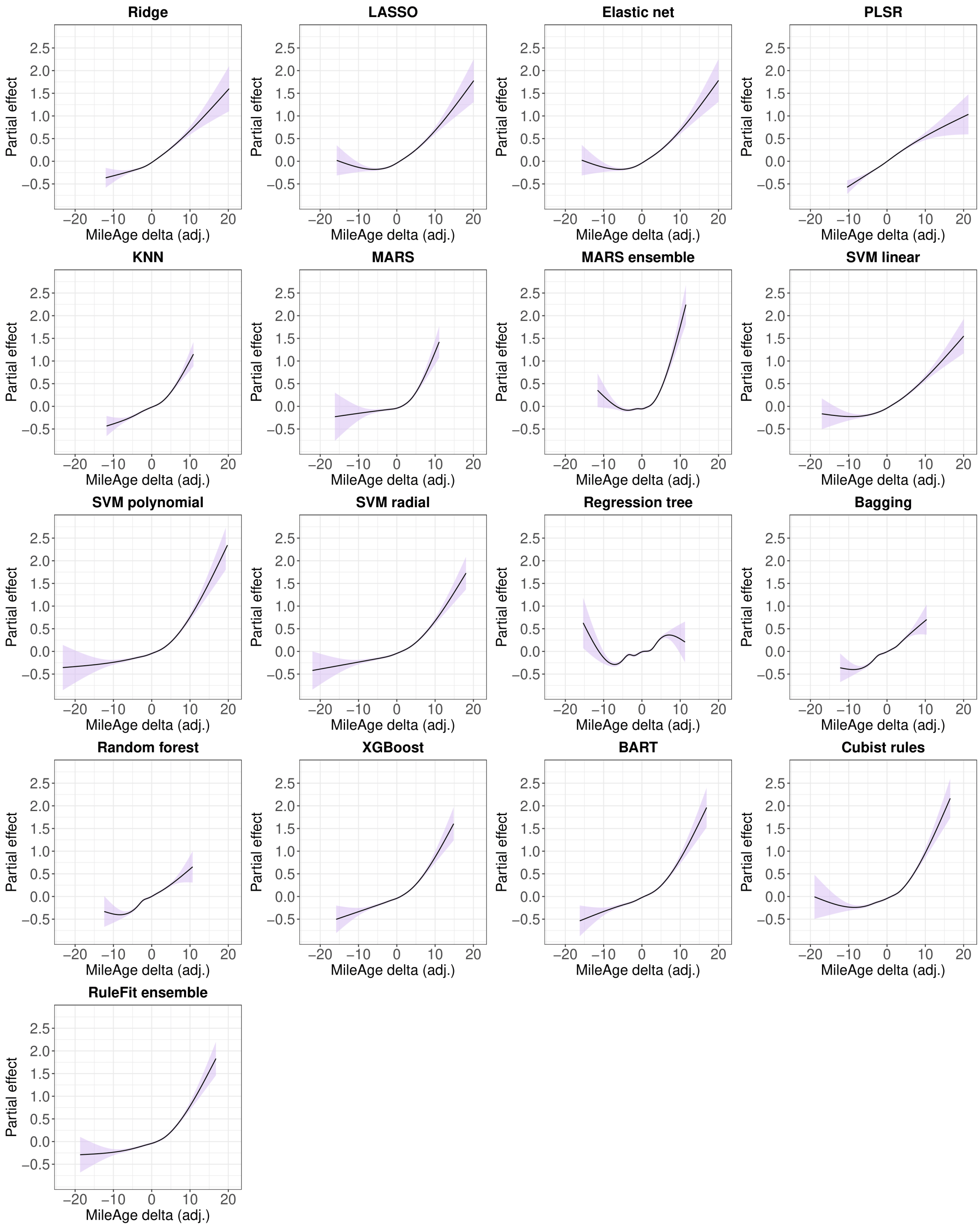

Figure S55. Partial effect plots of generalised additive models of the association between self-rated health and MileAge delta (adj.) for all models. Models were adjusted for chronological age and sex. The shaded areas correspond to 95% confidence intervals. See Box 1 for model abbreviations. $N = 100{,}789$.

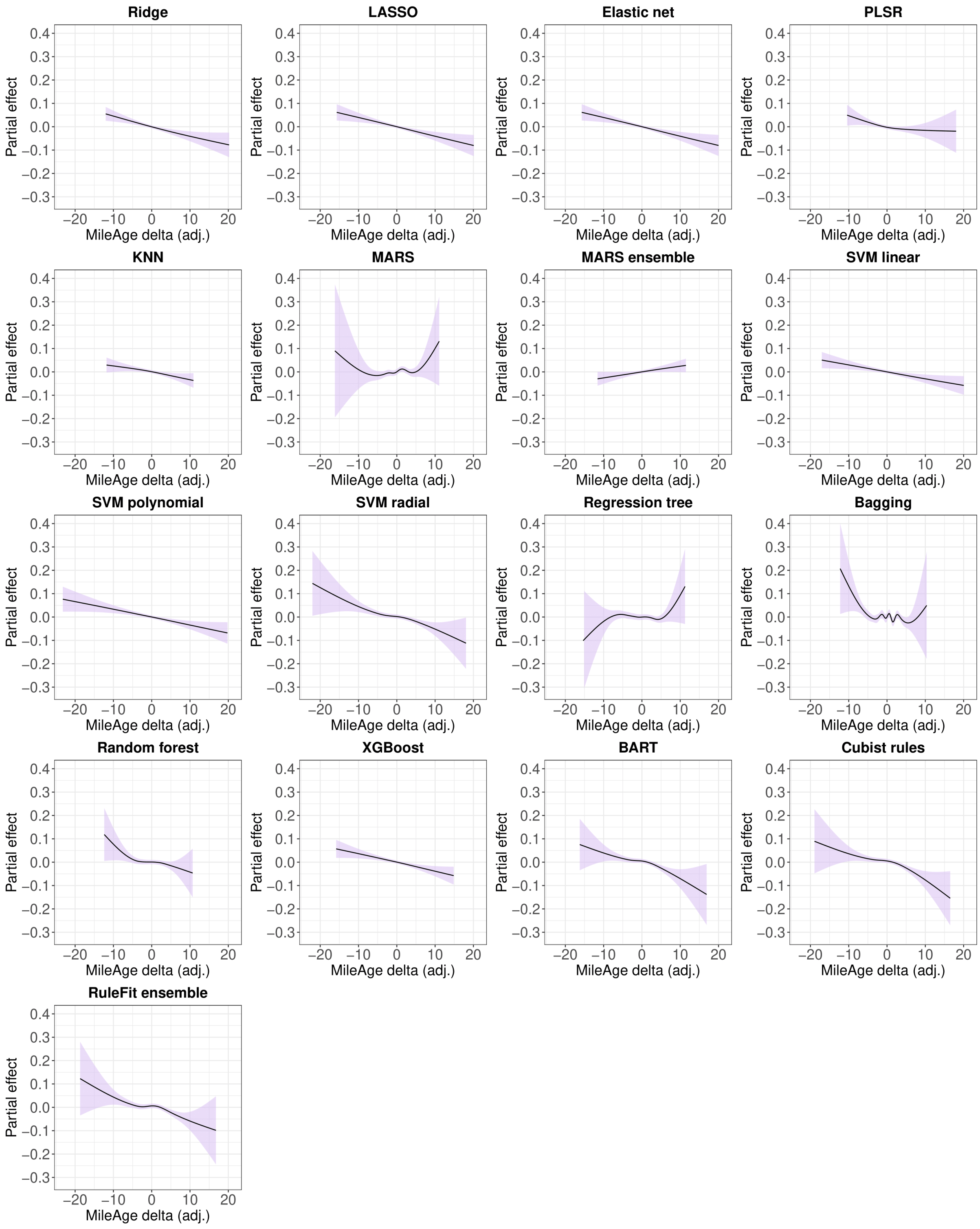

Figure S56. Partial effect plots of generalised additive models of the association between telomere length and MileAge delta (adj.) for all models. Models were adjusted for chronological age and sex. The shaded areas correspond to 95% confidence intervals. See Box 1 for model abbreviations. $N = 98{,}750$.

14. Age adjustment and time axis in the prospective analysis

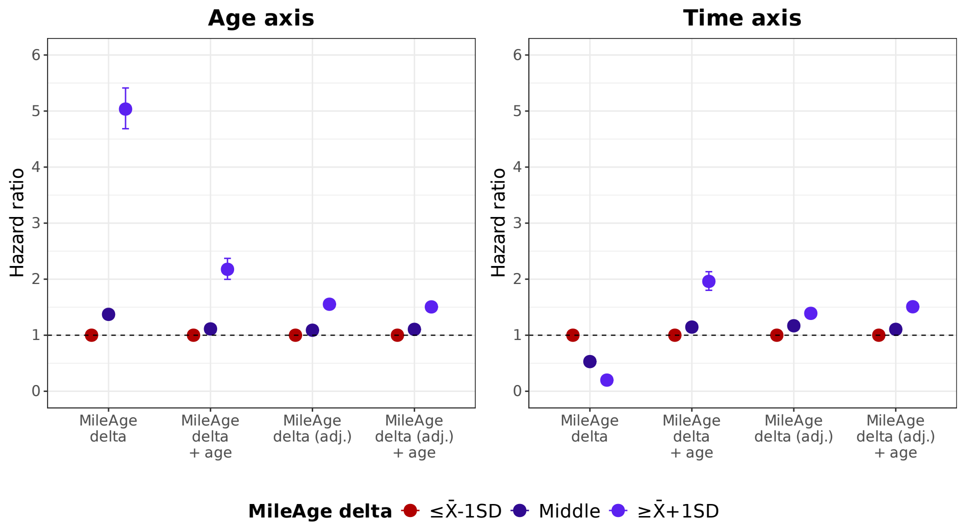

Figure S57. Hazard ratios (HR) and 95% confidence intervals from Cox proportional hazards models for all-cause mortality for unadjusted and age bias adjusted MileAge and MileAge delta (trained on $N = 225{,}212$ participants) with and without adjustment for chronological age as covariate, using age (in years) and time (in days) since the baseline assessment as the underlying time axis. All analyses were adjusted for sex. SD = standard deviation.

Table S13. All-cause mortality by age adjustment and time axis

Predictor	Level	N_{total}	N_{died}	HR	95% CI		*p*
Age (in years)							
MileAge delta	≤ $\bar{X}$-1SD	36691	5465		Reference		
	Middle	149333	11751	1.37	1.33	1.42	<0.001
	≥ $\bar{X}$+1SD	39188	1216	5.04	4.68	5.41	<0.001
MileAge delta + age	≤ $\bar{X}$-1SD	36691	5465		Reference		
	Middle	149333	11751	1.11	1.07	1.15	<0.001
	≥ $\bar{X}$+1SD	39188	1216	2.18	2.00	2.37	<0.001
MileAge delta (adj.)	≤ $\bar{X}$-1SD	36240	2590		Reference		
	Middle	153392	12495	1.09	1.04	1.14	<0.001
	≥ $\bar{X}$+1SD	35580	3347	1.55	1.47	1.64	<0.001
MileAge delta (adj.) + age	≤ $\bar{X}$-1SD	36240	2590		Reference		
	Middle	153392	12495	1.10	1.06	1.15	<0.001
	≥ $\bar{X}$+1SD	35580	3347	1.51	1.43	1.59	<0.001
Time since baseline assessment							
MileAge delta	≤ $\bar{X}$-1SD	36691	5465		Reference		
	Middle	149333	11751	0.53	0.51	0.55	<0.001
	≥ $\bar{X}$+1SD	39188	1216	0.20	0.19	0.21	<0.001
MileAge delta + age	≤ $\bar{X}$-1SD	36691	5465		Reference		
	Middle	149333	11751	1.15	1.10	1.19	<0.001
	≥ $\bar{X}$+1SD	39188	1216	1.96	1.80	2.13	<0.001
MileAge delta (adj.)	≤ $\bar{X}$-1SD	36240	2590		Reference		
	Middle	153392	12495	1.17	1.12	1.22	<0.001
	≥ $\bar{X}$+1SD	35580	3347	1.39	1.32	1.46	<0.001
MileAge delta (adj.) + age	≤ $\bar{X}$-1SD	36240	2590		Reference		
	Middle	153392	12495	1.10	1.06	1.15	<0.001
	≥ $\bar{X}$+1SD	35580	3347	1.51	1.43	1.59	<0.001

Note: HR = hazard ratio; CI = confidence interval; SD = standard deviation. MileAge delta derived from Cubist rule-based regression model (trained on N = 225,212 participants). All models were adjusted for sex.

16. Mortality by MileAge delta

Table S14. All-cause mortality by MileAge delta (adj.)

Model	Level	N_{total}	N_{died}	HR	95% CI		*p*
SVM radial	≤ X̄-1SD	15973	1161		Reference		
	Middle	69614	5565	1.07	1.01	1.15	0.026
	≥ X̄+1SD	15772	1472	1.43	1.32	1.54	<0.001
SVM polynomial	≤ X̄-1SD	15762	1143		Reference		
	Middle	69891	5539	1.08	1.01	1.15	0.017
	≥ X̄+1SD	15706	1516	1.50	1.38	1.62	<0.001
Cubist rules	≤ X̄-1SD	16204	1160		Reference		
	Middle	69166	5525	1.10	1.04	1.18	0.003
	≥ X̄+1SD	15989	1513	1.52	1.41	1.64	<0.001
RuleFit ensemble	≤ X̄-1SD	16116	1161		Reference		
	Middle	69394	5549	1.13	1.06	1.20	<0.001
	≥ X̄+1SD	15849	1488	1.45	1.34	1.57	<0.001
XGBoost	≤ X̄-1SD	16496	1178		Reference		
	Middle	68827	5550	1.10	1.03	1.17	0.005
	≥ X̄+1SD	16036	1470	1.44	1.33	1.56	<0.001
BART	≤ X̄-1SD	16174	1187		Reference		
	Middle	69287	5492	1.09	1.03	1.17	0.005
	≥ X̄+1SD	15898	1519	1.42	1.32	1.54	<0.001
SVM linear	≤ X̄-1SD	12579	949		Reference		
	Middle	56082	4308	1.08	1.01	1.16	0.030
	≥ X̄+1SD	12431	1269	1.50	1.38	1.63	<0.001
Elastic net	≤ X̄-1SD	15614	1197		Reference		
	Middle	70068	5432	1.08	1.02	1.15	0.012
	≥ X̄+1SD	15677	1569	1.46	1.36	1.58	<0.001
LASSO	≤ X̄-1SD	15593	1199		Reference		
	Middle	70114	5415	1.08	1.01	1.15	0.023
	≥ X̄+1SD	15652	1584	1.48	1.37	1.59	<0.001
Ridge	≤ X̄-1SD	15554	1147		Reference		
	Middle	70334	5478	1.14	1.07	1.22	<0.001
	≥ X̄+1SD	15471	1573	1.54	1.42	1.66	<0.001
Random forest	≤ X̄-1SD	16785	1246		Reference		
	Middle	68089	5661	1.14	1.07	1.21	<0.001
	≥ X̄+1SD	16485	1291	1.24	1.15	1.34	<0.001
Bagging	≤ X̄-1SD	17017	1237		Reference		
	Middle	67816	5688	1.14	1.07	1.21	<0.001
	≥ X̄+1SD	16526	1273	1.26	1.17	1.37	<0.001
PLSR	≤ X̄-1SD	15278	1091		Reference		
	Middle	70592	5583	1.25	1.17	1.33	<0.001
	≥ X̄+1SD	15489	1524	1.58	1.46	1.71	<0.001
Regression tree	≤ X̄-1SD	17885	1319		Reference		
	Middle	67060	5646	1.06	1.00	1.13	0.059
	≥ X̄+1SD	16414	1233	1.24	1.14	1.34	<0.001
KNN	≤ X̄-1SD	16669	1209		Reference		
	Middle	68220	5585	1.12	1.05	1.19	<0.001
	≥ X̄+1SD	16470	1404	1.31	1.21	1.42	<0.001

MARS	≤ $\bar{X}$-1SD	15962	1353	Reference			
	Middle	69618	5615	0.94	0.89	1.00	0.054
	≥ $\bar{X}$+1SD	15779	1230	1.12	1.03	1.21	0.006
MARS ensemble	≤ $\bar{X}$-1SD	14718	1301	Reference			
	Middle	62021	4894	0.94	0.88	1.00	0.050
	≥ $\bar{X}$+1SD	14486	1196	1.10	1.02	1.19	0.017

Note: HR = hazard ratio; CI = confidence interval; SD = standard deviation. Models were adjusted for chronological age and sex. Age (in years) was used as the underlying time axis. See Box 1 for model abbreviations. *N* = 101,274.

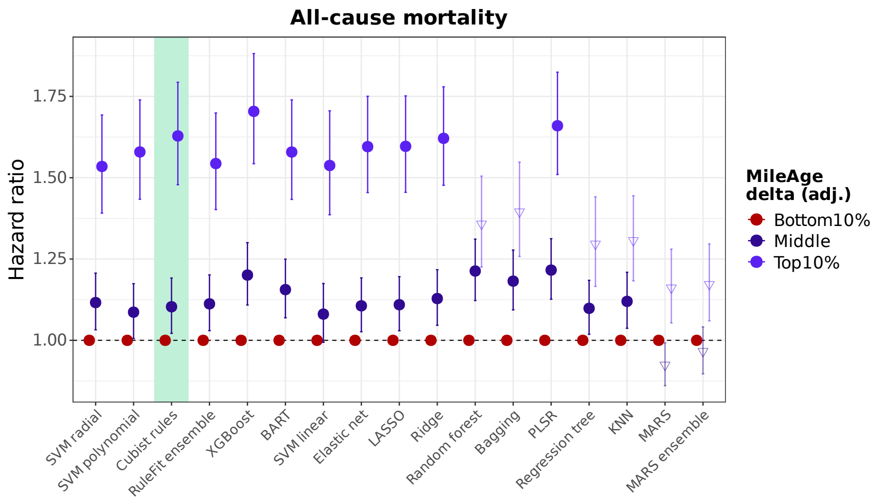

Figure S58. Hazard ratios (HR) and 95% confidence intervals from Cox proportional hazards models for all models. Models were adjusted for chronological age and sex. Age (in years) was used as the underlying time axis. Reference group: individuals with a MileAge delta (adj.) in the bottom 10% of the distribution. Downward triangles indicate hazard ratios smaller than the hazard ratio for the Cubist rule-based regression model (at the same level), based on a Wald p-value <0.05. See Box 1 for model abbreviations. $N = 101{,}274$.

Table S15. All-cause mortality by MileAge delta (adj.) – distribution tails

Model	Level	N_{total}	N_{died}	HR	95% CI		*p*
SVM radial	Bottom 10%	10136	712		Reference		
	Middle	81087	6540	1.12	1.03	1.21	0.006
	Top 10%	10136	946	1.53	1.39	1.69	<0.001
SVM polynomial	Bottom 10%	10136	722		Reference		
	Middle	81087	6468	1.09	1.01	1.17	0.036
	Top 10%	10136	1008	1.58	1.43	1.74	<0.001
Cubist rules	Bottom 10%	10136	730		Reference		
	Middle	81087	6484	1.10	1.02	1.19	0.012
	Top 10%	10136	984	1.63	1.48	1.79	<0.001
RuleFit ensemble	Bottom 10%	10136	733		Reference		
	Middle	81087	6465	1.11	1.03	1.20	0.007
	Top 10%	10136	1000	1.54	1.40	1.70	<0.001
XGBoost	Bottom 10%	10136	677		Reference		
	Middle	81087	6562	1.20	1.11	1.30	<0.001
	Top 10%	10136	959	1.70	1.54	1.88	<0.001
BART	Bottom 10%	10136	711		Reference		
	Middle	81087	6476	1.16	1.07	1.25	<0.001
	Top 10%	10136	1011	1.58	1.43	1.74	<0.001
SVM linear	Bottom 10%	8110	626		Reference		
	Middle	64872	5042	1.08	0.99	1.17	0.069
	Top 10%	8110	858	1.54	1.39	1.71	<0.001
Elastic net	Bottom 10%	10136	782		Reference		
	Middle	81087	6325	1.11	1.03	1.19	0.008
	Top 10%	10136	1091	1.60	1.45	1.75	<0.001
LASSO	Bottom 10%	10136	781		Reference		
	Middle	81087	6330	1.11	1.03	1.20	0.006
	Top 10%	10136	1087	1.60	1.46	1.75	<0.001
Ridge	Bottom 10%	10136	761		Reference		
	Middle	81087	6340	1.13	1.05	1.22	0.002
	Top 10%	10136	1097	1.62	1.48	1.78	<0.001
Random forest	Bottom 10%	10136	716		Reference		
	Middle	81087	6709	1.21	1.12	1.31	<0.001
	Top 10%	10136	773	1.36	1.23	1.51	<0.001
Bagging	Bottom 10%	10136	714		Reference		
	Middle	81087	6740	1.18	1.09	1.28	<0.001
	Top 10%	10136	744	1.40	1.26	1.55	<0.001
PLSR	Bottom 10%	10136	746		Reference		
	Middle	81087	6391	1.22	1.13	1.31	<0.001
	Top 10%	10136	1061	1.66	1.51	1.82	<0.001
Regression tree	Bottom 10%	10136	768		Reference		
	Middle	81085	6776	1.10	1.02	1.18	0.015
	Top 10%	10138	654	1.30	1.17	1.44	<0.001
KNN	Bottom 10%	10136	730		Reference		
	Middle	81087	6633	1.12	1.04	1.21	0.004
	Top 10%	10136	835	1.31	1.18	1.44	<0.001
MARS	Bottom 10%	10136	873		Reference		

	Middle	81087	6528	0.92	0.86	0.99	0.030
	Top 10%	10136	797	1.16	1.05	1.28	0.003
MARS ensemble	Bottom 10%	9123	804		Reference		
	Middle	72979	5825	0.97	0.90	1.04	0.369
	Top 10%	9123	762	1.17	1.06	1.30	0.002

Note: HR = hazard ratio; CI = confidence interval; SD = standard deviation. Models were adjusted for chronological age and sex. Age (in years) was used as the underlying time axis. See Box 1 for model abbreviations. *N* = 101,274.

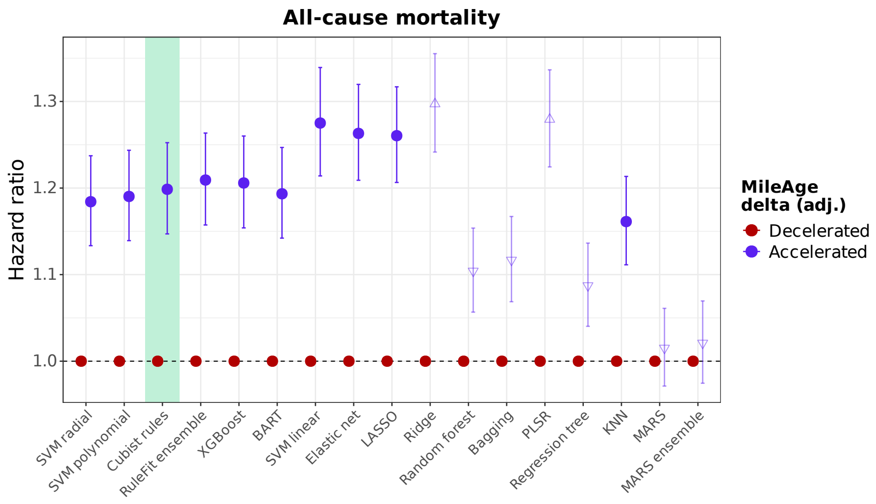

Figure S59. Hazard ratios (HR) and 95% confidence intervals from Cox proportional hazards models for all models. Models were adjusted for chronological age and sex. Age (in years) was used as the underlying time axis. Reference group: individuals with a negative MileAge delta (adj.), i.e., individuals with a younger predicted than true chronological age. Upward triangles indicate hazard ratios larger than the hazard ratio for the Cubist rule-based regression model (at the same level), while downward triangles indicate smaller hazard ratios, based on a Wald p-value <0.05. See Box 1 for model abbreviations. N = 101,274.

Table S16. All-cause mortality by MileAge delta (adj.) – direction

Model	Level	N total	N died	HR	95% CI		*p*
SVM radial	Decelerated	48972	3709		Reference		
	Accelerated	52387	4489	1.18	1.13	1.24	<0.001
SVM polynomial	Decelerated	49118	3718		Reference		
	Accelerated	52241	4480	1.19	1.14	1.24	<0.001
Cubist rules	Decelerated	49242	3704		Reference		
	Accelerated	52117	4494	1.20	1.15	1.25	<0.001
RuleFit ensemble	Decelerated	49343	3705		Reference		
	Accelerated	52016	4493	1.21	1.16	1.26	<0.001
XGBoost	Decelerated	48623	3623		Reference		
	Accelerated	52736	4575	1.21	1.15	1.26	<0.001
BART	Decelerated	49738	3765		Reference		
	Accelerated	51621	4433	1.19	1.14	1.25	<0.001
SVM linear	Decelerated	41541	3025		Reference		
	Accelerated	39551	3501	1.28	1.21	1.34	<0.001
Elastic net	Decelerated	51816	3830		Reference		
	Accelerated	49543	4368	1.26	1.21	1.32	<0.001
LASSO	Decelerated	51821	3833		Reference		
	Accelerated	49538	4365	1.26	1.21	1.32	<0.001
Ridge	Decelerated	52407	3821		Reference		
	Accelerated	48952	4377	1.30	1.24	1.36	<0.001
Random forest	Decelerated	48858	3831		Reference		
	Accelerated	52501	4367	1.10	1.06	1.15	<0.001
Bagging	Decelerated	47986	3726		Reference		
	Accelerated	53373	4472	1.12	1.07	1.17	<0.001
PLSR	Decelerated	53397	3930		Reference		
	Accelerated	47962	4268	1.28	1.22	1.34	<0.001
Regression tree	Decelerated	46221	3550		Reference		
	Accelerated	55138	4648	1.09	1.04	1.14	<0.001
KNN	Decelerated	48566	3729		Reference		
	Accelerated	52793	4469	1.16	1.11	1.21	<0.001
MARS	Decelerated	48232	4063		Reference		
	Accelerated	53127	4135	1.02	0.97	1.06	0.504
MARS ensemble	Decelerated	44397	3695		Reference		
	Accelerated	46828	3696	1.02	0.97	1.07	0.382

Note: HR = hazard ratio; CI = confidence interval; SD = standard deviation. Models were adjusted for chronological age and sex. Age (in years) was used as the underlying time axis. See Box 1 for model abbreviations. *N* = 101,274.

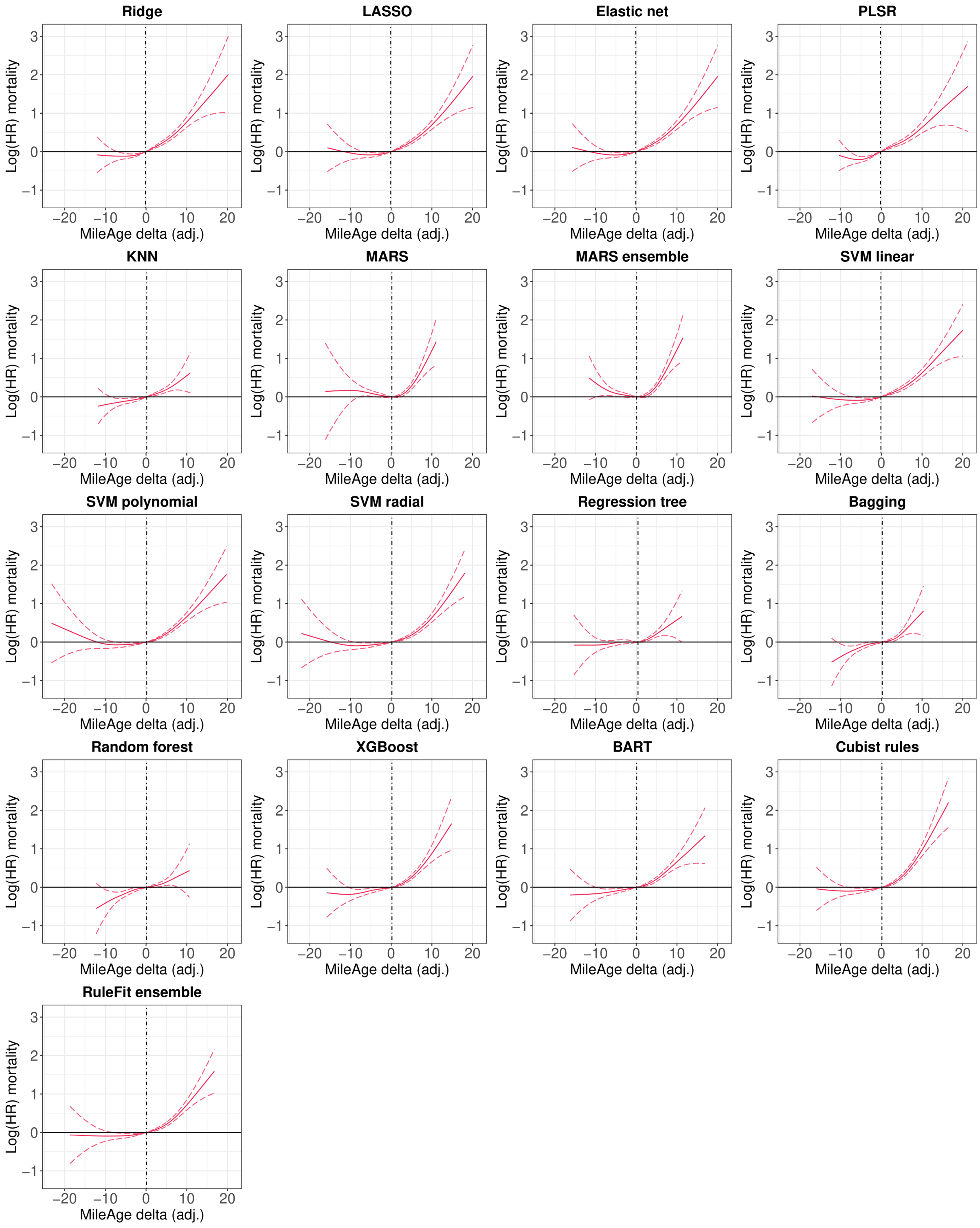

Figure S60. Log(HRs) and 95% confidence intervals from Cox proportional hazards models for all-cause mortality. Models were adjusted for chronological age and sex. Age (in years) was used as the underlying time axis. A penalised spline function with four degrees of freedom was used. Vertical lines indicate the median of the distribution which represents the reference for interpreting the estimates shown. HR = hazard ratio. See Box 1 for model abbreviations. *N* = 101,274.

17. MileAge delta stratified by sex

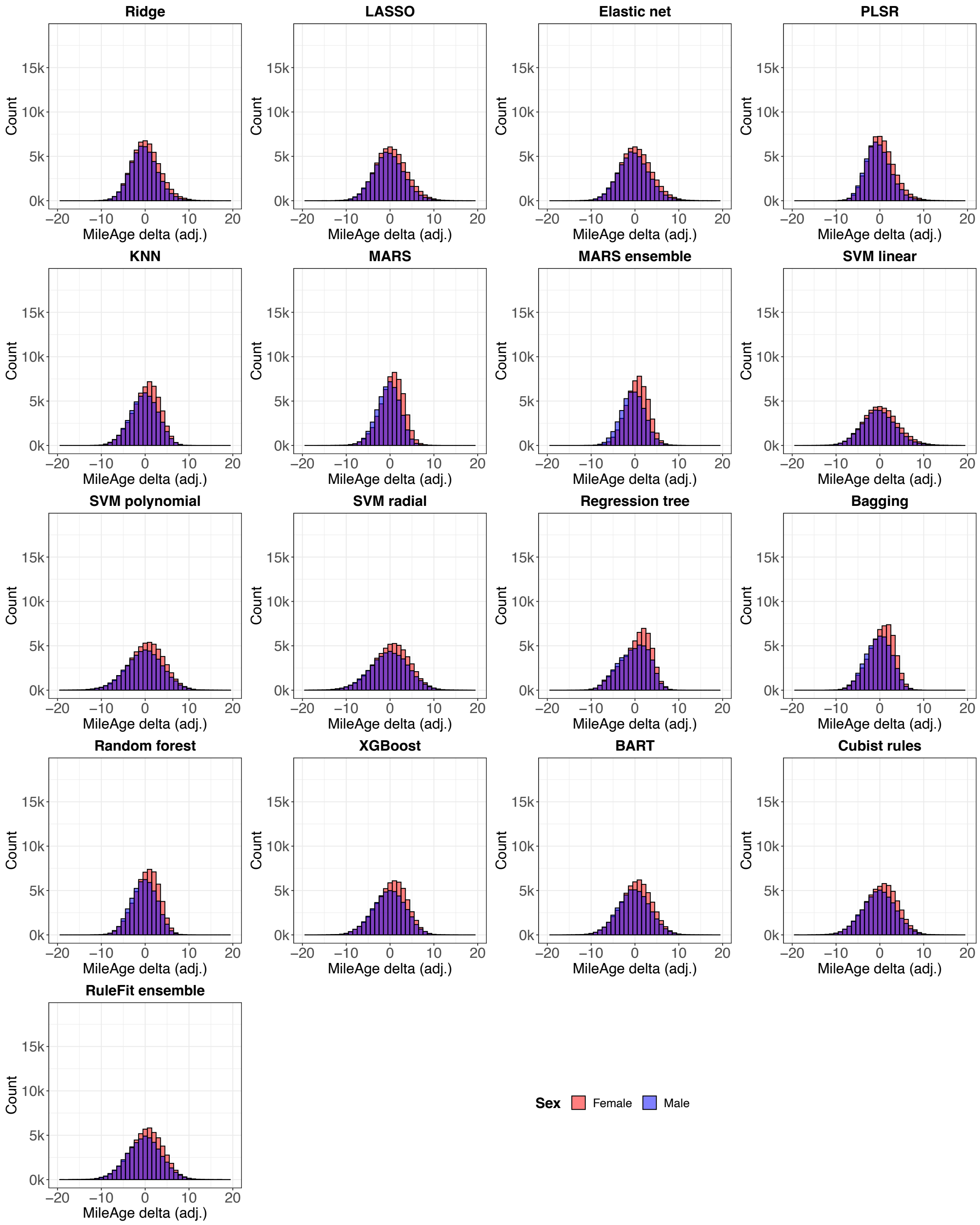

Figure S61. Histograms showing the distribution of MileAge delta (adj.) for all models, stratified by sex. See Box 1 for model abbreviations. $N = 101{,}359$.

18. Mortality by MileAge delta stratified

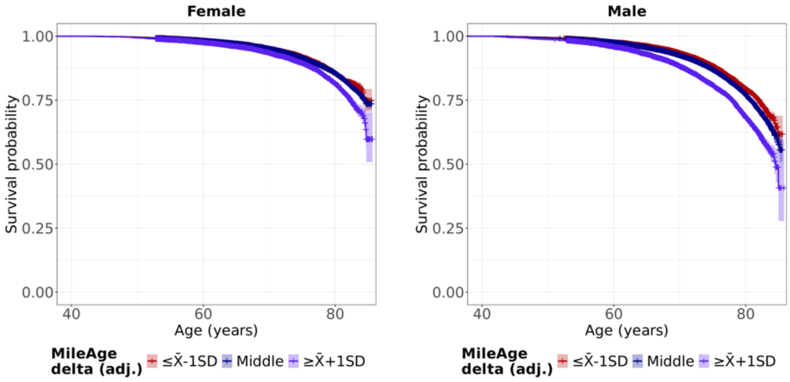

Figure S62. Kaplan-Meier survival probabilities for all-cause mortality by MileAge delta (adj.) derived from the Cubist rule-based regression model (trained on $N = 225{,}212$ participants) stratified by sex. Log-rank test p-values < 0.001. Age (in years) was used as the underlying time axis. Reference group: individuals with a MileAge delta (adj.) smaller than one standard deviation below the mean. SD = standard deviation. $N = 224{,}996$.

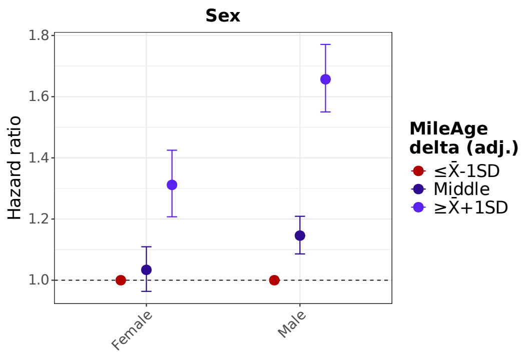

Figure S63. Hazard ratios (HR) and 95% confidence intervals from Cox proportional hazards models derived from the Cubist rule-based regression model (trained on N = 225,212 participants) stratified by sex. Models were adjusted for chronological age. Age (in years) was used as the underlying time axis. Reference group: individuals with a MileAge delta (adj.) smaller than one standard deviation below the mean. SD = standard deviation. N = 224,996.

Table S17. All-cause mortality by MileAge delta (adj.) stratified

Sex	Level	N_{total}	N_{died}	HR	95% CI		p
Female	≤ X̄-1SD	18330	924		Reference		
	Middle	82499	5249	1.03	0.96	1.11	0.354
	≥ X̄+1SD	20702	1474	1.31	1.21	1.42	<0.001
Male	≤ X̄-1SD	17910	1666		Reference		
	Middle	70893	7246	1.15	1.09	1.21	<0.001
	≥ X̄+1SD	14878	1873	1.66	1.55	1.77	<0.001
Self-rated health							
Poor	≤ X̄-1SD	1079	208		Reference		
	Middle	5700	1193	1.12	0.97	1.30	0.133
	≥ X̄+1SD	2431	637	1.56	1.33	1.83	<0.001
Fair	≤ X̄-1SD	6412	660		Reference		
	Middle	31129	3591	1.08	1.00	1.18	0.061
	≥ X̄+1SD	8572	1121	1.41	1.28	1.55	<0.001
Good	≤ X̄-1SD	21767	1415		Reference		
	Middle	90458	6334	1.04	0.98	1.10	0.226
	≥ X̄+1SD	19372	1333	1.21	1.12	1.31	<0.001
Excellent	≤ X̄-1SD	6772	285		Reference		
	Middle	25289	1263	1.13	0.99	1.28	0.067
	≥ X̄+1SD	4945	211	1.17	0.97	1.40	0.096
Age group							
37-49 years	≤ X̄-1SD	8782	174		Reference		
	Middle	30219	609	0.97	0.82	1.15	0.748
	≥ X̄+1SD	8157	223	1.35	1.11	1.65	0.003
50-59 years	≤ X̄-1SD	10667	427		Reference		
	Middle	43689	2026	1.18	1.07	1.32	0.002
	≥ X̄+1SD	11406	756	1.79	1.59	2.02	<0.001
60-71 years	≤ X̄-1SD	14302	1858		Reference		
	Middle	69187	9343	1.12	1.06	1.17	<0.001
	≥ X̄+1SD	13517	2196	1.48	1.39	1.58	<0.001

Note: HR = hazard ratio; CI = confidence interval; SD = standard deviation. MileAge delta (adj.) derived from Cubist rule-based regression model (trained on $N = 225,212$ participants). Models were adjusted for chronological age (sex stratified), sex (age group stratified) and age and sex (self-rated health stratified). Age (in years) was used as the underlying time axis. $N = 224,996$, except for self-rated health ($N = 209,554$).

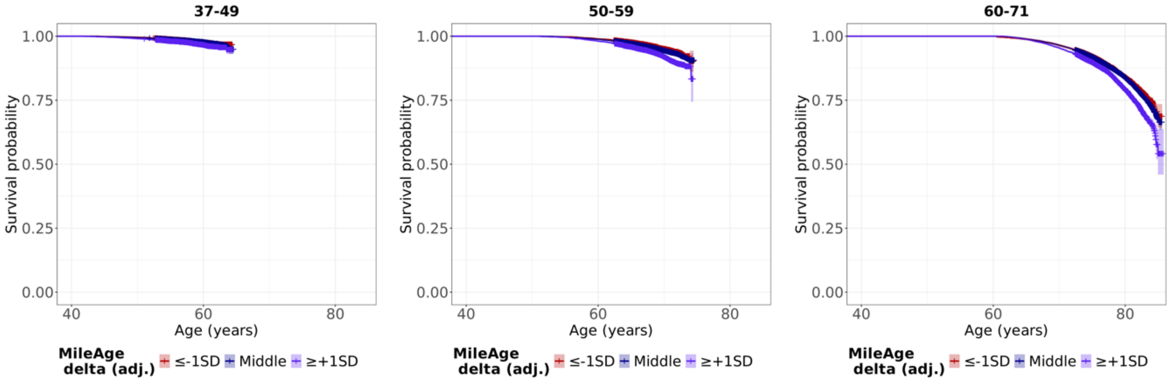

Figure S64. Kaplan-Meier survival probabilities for all-cause mortality by MileAge delta (adj.) derived from the Cubist rule-based regression model (trained on $N = 225{,}212$ participants) stratified by chronological age group. Log-rank test p-values < 0.001. Age (in years) was used as the underlying time axis. Reference group: individuals with a MileAge delta (adj.) smaller than one standard deviation below the mean. SD = standard deviation. $N = 224{,}996$.

19. Mortality by MileAge delta and other non-metabolomic ageing markers

Table S18. All-cause mortality by MileAge delta (adj.) compared with non-metabolomic ageing markers

Predictor	Level	N_{total}	N_{died}	HR	95% CI		*p*
MileAge delta (adj.)	≤ $\bar{X}$-1SD	36240	2590		Reference		
	Middle	153392	12495	1.10	1.06	1.15	<0.001
	≥ $\bar{X}$+1SD	35580	3347	1.51	1.43	1.59	<0.001
Frailty index	≤ $\bar{X}$-1SD	32451	1492		Reference		
	Middle	157913	11698	1.44	1.37	1.52	<0.001
	≥ $\bar{X}$+1SD	34130	5170	2.78	2.62	2.95	<0.001
Grip strength	≤ $\bar{X}$-1SD	41134	2844		Reference		
	Middle	146820	11942	1.35	1.30	1.41	<0.001
	≥ $\bar{X}$+1SD	36377	3545	1.89	1.78	2.01	<0.001
Telomere length	≤ $\bar{X}$-1SD	32369	1920		Reference		
	Middle	152240	12012	1.03	0.98	1.08	0.188
	≥ $\bar{X}$+1SD	32627	3856	1.26	1.19	1.33	<0.001

Note: HR = hazard ratio; CI = confidence interval; SD = standard deviation. MileAge delta (adj.) derived from Cubist rule-based regression model (trained on N = 225,212 participants). Models were adjusted for chronological age and sex. Age (in years) was used as the underlying time axis. N = 224,996 (MileAge); N = 224,278 (frailty index); N = 224,117 (grip strength); N = 217,030 (telomere length).

Table S19. C-index for all-cause mortality

Model	C-index	95% CI	
Base model (age + sex)	0.717	0.714	0.721
+ Telomere length	0.717	0.714	0.721
+ MileAge delta (adj.)	0.720	0.716	0.723
+ Grip strength	0.722	0.719	0.726
+ Frailty index	0.736	0.732	0.739

Note: CI = confidence interval. MileAge (adj.) derived from Cubist rule-based regression model (trained on 225,212 participants). Age (in years) was used as the underlying time axis. N = 224,996 (base model and MileAge); N = 224,278 (frailty index); N = 224,117 (grip strength); N = 217,030 (telomere length).

20. Comparison with other metabolomic scores

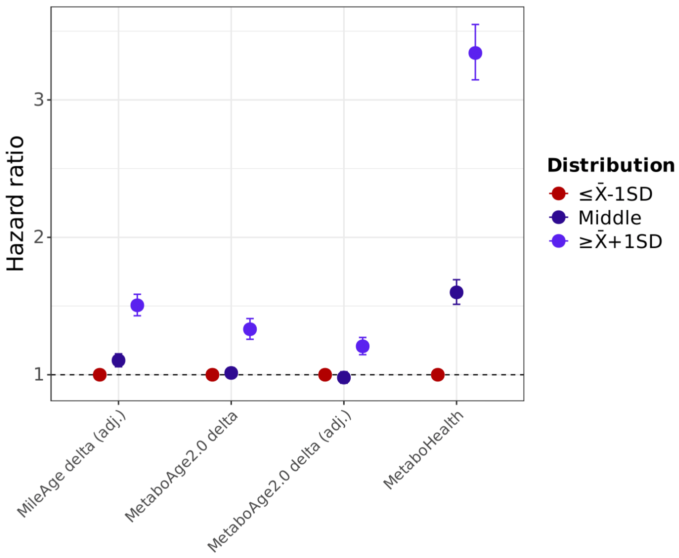

Figure S65. Hazard ratios (HR) and 95% confidence intervals from Cox proportional hazards models for all-cause mortality by MileAge delta (adj.) derived from the Cubist rule-based regression model (trained on $N = 225{,}212$ participants), MetaboAge 2.0 delta, MetaboAge 2.0 delta (adj.) and MetaboHealth. Models were adjusted for chronological age and sex. Age (in years) was used as the underlying time axis. Reference group: individuals with a score smaller than one standard deviation below the mean. $N = 224{,}996$.

Table S20. All-cause mortality by MileAge delta (adj.) compared with other metabolomic scores

Predictor	Level	N_{total}	N_{died}	HR	95% CI		*p*
MileAge delta (adj.)	≤-1SD	36240	2590	Reference			
	Middle	153392	12495	1.10	1.06	1.15	<0.001
	≥+1SD	35580	3347	1.51	1.43	1.59	<0.001
MetaboAge2.0	≤-1SD	34686	3850	Reference			
	Middle	155741	12451	1.01	0.98	1.05	0.495
	≥+1SD	34785	2131	1.33	1.26	1.41	<0.001
MetaboAge2.0 (adj.)	≤-1SD	34492	2592	Reference			
	Middle	156367	12480	0.98	0.94	1.02	0.340
	≥+1SD	34353	3360	1.21	1.15	1.27	<0.001
MetaboHealth	≤-1SD	33611	1418	Reference			
	Middle	157902	11755	1.60	1.51	1.69	<0.001
	≥+1SD	33699	5259	3.34	3.15	3.55	<0.001

Note: HR = hazard ratio; CI = confidence interval; SD = standard deviation. MileAge delta (adj.) derived from Cubist rule-based regression model (trained on $N = 225{,}212$ participants). Models were adjusted for chronological age and sex. Age (in years) was used as the underlying time axis. $N = 224{,}996$.

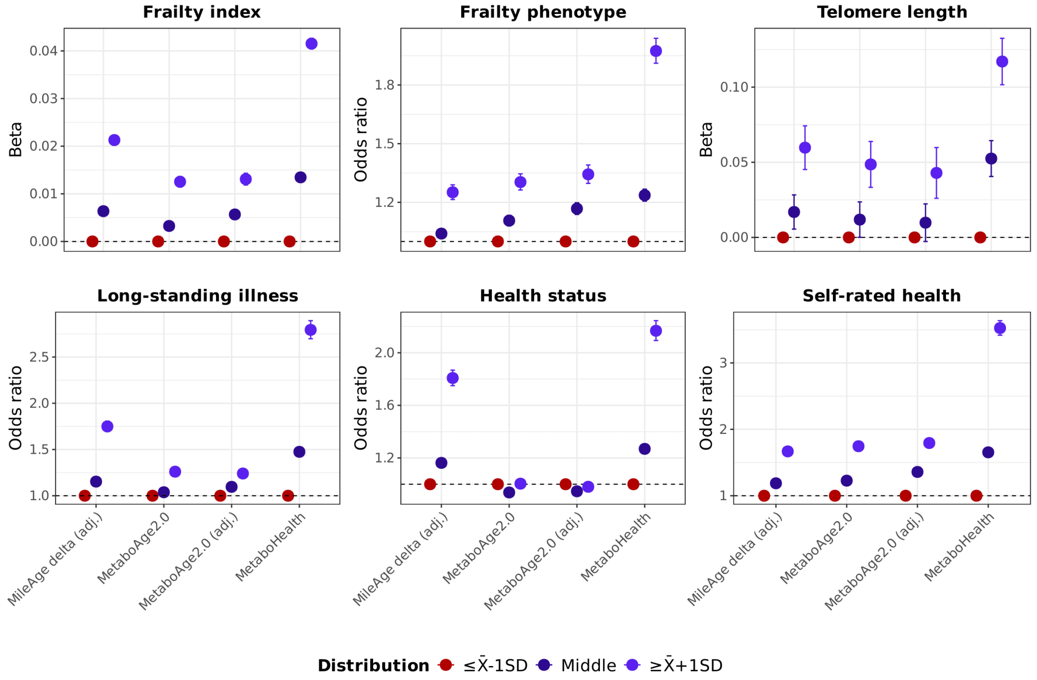

Figure S66. Associations between MileAge delta (adj.) derived from the Cubist rule-based regression model (trained on $N = 225{,}212$ participants) and health indicators, compared to MetaboAge 2.0 delta, MetaboAge 2.0 delta (adj.) and MetaboHealth. Models were adjusted for chronological age and sex. Reference group: individuals with a score smaller than one standard deviation below the mean. $N = 224{,}278$ (frailty index); $N = 211{,}192$ (frailty phenotype); $N = 217{,}030$ (telomere length); $N = 219{,}278$ (long-standing illness); $N = 219{,}046$ (health status); $N = 223{,}711$ (self-rated health).

Table S21. Associations between MileAge delta (adj.) and health indicators compared with other metabolomic scores

**Frailty index**	Level	*β*	95% CI		*p*
MileAge delta (adj.)	≤-1SD		Reference		
	Middle	0.006	0.006	0.007	<0.001
	≥+1SD	0.021	0.020	0.022	<0.001
MetaboAge 2.0 delta	≤-1SD		Reference		
	Middle	0.003	0.002	0.004	<0.001
	≥+1SD	0.013	0.011	0.014	<0.001
MetaboAge 2.0 delta (adj.)	≤-1SD		Reference		
	Middle	0.006	0.005	0.007	<0.001
	≥+1SD	0.013	0.012	0.014	<0.001
MetaboHealth	≤-1SD		Reference		
	Middle	0.013	0.013	0.014	<0.001
	≥+1SD	0.042	0.040	0.043	<0.001
**Frailty phenotype**		OR	95% CI		*p*
MileAge delta (adj.)	≤-1SD		Reference		
	Middle	1.04	1.02	1.06	<0.001
	≥+1SD	1.25	1.21	1.29	<0.001
MetaboAge 2.0 delta	≤-1SD		Reference		
	Middle	1.11	1.08	1.13	<0.001
	≥+1SD	1.30	1.26	1.35	<0.001
MetaboAge 2.0 delta (adj.)	≤-1SD		Reference		
	Middle	1.17	1.14	1.20	<0.001
	≥+1SD	1.34	1.30	1.39	<0.001
MetaboHealth	≤-1SD		Reference		
	Middle	1.24	1.21	1.27	<0.001
	≥+1SD	1.97	1.91	2.04	<0.001
**Telomere length**		*β*	95% CI		*p*
MileAge delta (adj.)	≤-1SD		Reference		
	Middle	0.017	0.006	0.028	0.004
	≥+1SD	0.060	0.045	0.074	<0.001
MetaboAge 2.0 delta	≤-1SD		Reference		
	Middle	0.012	0.000	0.024	0.048
	≥+1SD	0.049	0.033	0.064	<0.001
MetaboAge 2.0 delta (adj.)	≤-1SD		Reference		
	Middle	0.010	-0.003	0.022	0.124
	≥+1SD	0.043	0.026	0.060	<0.001
MetaboHealth	≤-1SD		Reference		
	Middle	0.052	0.041	0.064	<0.001
	≥+1SD	0.117	0.102	0.132	<0.001
**Long-standing illness**		OR	95% CI		*p*
MileAge delta (adj.)	≤-1SD		Reference		
	Middle	1.15	1.12	1.18	<0.001
	≥+1SD	1.75	1.69	1.81	<0.001
MetaboAge 2.0 delta	≤-1SD		Reference		
	Middle	1.04	1.01	1.07	0.007
	≥+1SD	1.26	1.22	1.30	<0.001

MetaboAge 2.0 delta (adj.)	≤-1SD	Reference			
	Middle	1.10	1.06	1.13	<0.001
	≥+1SD	1.24	1.19	1.29	<0.001
MetaboHealth	≤-1SD	Reference			
	Middle	1.47	1.43	1.52	<0.001
	≥+1SD	2.79	2.70	2.89	<0.001
**Health status**		OR	95% CI		*p*
MileAge delta (adj.)	≤-1SD	Reference			
	Middle	1.16	1.13	1.19	<0.001
	≥+1SD	1.81	1.75	1.87	<0.001
MetaboAge 2.0 delta	≤-1SD	Reference			
	Middle	0.94	0.91	0.96	<0.001
	≥+1SD	1.00	0.97	1.04	0.808
MetaboAge 2.0 delta (adj.)	≤-1SD	Reference			
	Middle	0.95	0.92	0.97	<0.001
	≥+1SD	0.98	0.94	1.02	0.313
MetaboHealth	≤-1SD	Reference			
	Middle	1.27	1.23	1.31	<0.001
	≥+1SD	2.17	2.09	2.24	<0.001
**Self-rated health**		OR	95% CI		*p*
MileAge delta (adj.)	≤-1SD	Reference			
	Middle	1.19	1.16	1.21	<0.001
	≥+1SD	1.67	1.62	1.72	<0.001
MetaboAge 2.0 delta	≤-1SD	Reference			
	Middle	1.23	1.20	1.26	<0.001
	≥+1SD	1.75	1.69	1.80	<0.001
MetaboAge 2.0 delta (adj.)	≤-1SD	Reference			
	Middle	1.36	1.32	1.39	<0.001
	≥+1SD	1.79	1.73	1.85	<0.001
MetaboHealth	≤-1SD	Reference			
	Middle	1.65	1.61	1.69	<0.001
	≥+1SD	3.53	3.42	3.64	<0.001

Note: CI = confidence interval; SD = standard deviation. MileAge delta (adj.) derived from Cubist rule-based regression model (trained on 225,212 participants). Models were adjusted for chronological age and sex. N = 224,278 (frailty index); N = 211,192 (frailty phenotype); N = 217,030 (telomere length); N = 219,278 (long-standing illness); N = 219,046 (health status); N = 223,711 (self-rated health).

21. Additional data files

Data S1. Associations between chronological age and metabolite levels. Metabolites were standardised to have a mean equal to zero and a standard deviation of one. Associations were estimated using ordinary least squares regression. Models were adjusted for sex. *P*-values were $-\log_{10}$ transformed. $N = 101{,}359$.

Data S2. Associations between metabolite levels and all-cause mortality. Metabolites were standardised to have a mean equal to zero and a standard deviation of one. Hazard ratios were estimated using Cox proportional hazards models adjusted for sex, with age (in years) as the underlying time axis. *P*-values were $-\log_{10}$ transformed. $N = 101{,}274$.
